# Spatial and single-cell transcriptomics reveal a HIF-1α/NF-κB-driven hypoxia-induced senescence axis in BPH epithelium

**DOI:** 10.7150/ijbs.130242

**Published:** 2026-06-10

**Authors:** Zheng Li, Shuai Hu, Zhifu Liu, Muqiu Zhang, Shengbin Chen, Wei Yu, Mingxia Ding, Yu Fan, Senmao Li, Jie Jin

**Affiliations:** 1 Department of Urology, Peking University First Hospital, Institute of Urology, Peking University, Beijing Key Laboratory of Urogenital Diseases (Male), Molecular Diagnosis and Treatment Center, National Research Center for Genitourinary Oncology, Beijing 100034, China.; 2 Department of Urology, Hunan Provincial People's Hospital, The First Affiliated Hospital of Hunan Normal University, Changsha, China.; 3 Department of Urology, The First People's Hospital of Yunnan Province, Kunming, 650032, Yunnan, China.; 4 The Affiliated Hospital of Kunming University of Science and Technology, Kunming 650032, Yunnan, China.; 5 Department of Urology, The Second Affiliated Hospital of Kunming Medical University, No. 347 Dianmian Street, Wuhua District, Kunming, Yunnan, China.

**Keywords:** benign prostatic hyperplasia, hypoxia, epithelial senescence, HIF-1α, NF-κB, SASP, epithelial-stromal crosstalk, single-cell RNA sequencing, spatial transcriptomics

## Abstract

Benign prostatic hyperplasia (BPH) is an age-related prostate disorder with incompletely defined mechanisms. We integrated laser capture microdissection RNA sequencing, public single-cell RNA sequencing, public spatial transcriptomics, human tissue validation, mouse-model analyses, and *in vitro* perturbation assays to characterize hypoxia-associated epithelial senescence in BPH. SA-β-gal-positive cells were predominantly epithelial. RNA sequencing of SA-β-gal-positive epithelial cells revealed enrichment of hypoxia/HIF-1, NF-κB, cell-cycle arrest, and senescence-associated programs, with increased *HIF1A*, senescence-marker, and SASP-associated gene expression. Single-cell and spatial analyses showed coordinated activation of hypoxia-response, NF-κB-related, and senescence-associated programs in luminal epithelial cells or luminal-dominant spots. In 52 human BPH specimens, epithelial HIF-1α staining was modestly but significantly associated with p21, p27, and Rb. *In vitro*, 1% O₂ exposure or *HIF1A* overexpression induced senescence-associated phenotypes in BPH-1 and RWPE-1 cells, whereas HIF-1α inhibition or *HIF1A* knockdown attenuated these effects. NF-κB inhibition or *RELA* knockdown partially reversed HIF-1α-associated senescence phenotypes. Conditioned media from hypoxia-treated epithelial cells promoted stromal-cell proliferation and SASP-associated cytokine secretion in a partly *HIF1A*-dependent manner. In a testosterone propionate-induced BPH-like mouse model, HIF-1α or NF-κB inhibition attenuated prostatic hyperplasia, epithelial remodeling, and senescence-marker expression. These findings link hypoxia-associated HIF-1α/NF-κB signaling to epithelial senescence and epithelial-stromal crosstalk in BPH.

## Introduction

Benign prostatic hyperplasia (BPH) is a prevalent condition that affects around 20% of men aged 40-50 years, with incidence rising to over 80% in men aged 70 years and older, significantly impacting their quality of life 1-3. Histopathologically, BPH is characterized by an increase in epithelial and stromal cells in the periurethral area of the prostate, leading to a progressive worsening of lower urinary tract symptoms (LUTS) over time 4-6. Several factors have been implicated in the underlying pathogenesis of BPH, including cellular senescence, inflammation, and hormonal alterations 7,8. Cellular senescence, which accumulates with age and other cellular stressors 9,10, has been detected in nearly all human BPH prostate samples, and specific senescence-associated secretory phenotypes (SASPs), including proinflammatory cytokines such as interleukin-1 alpha (IL-1α) and interleukin-8 (IL-8), have been implicated in the progression of BPH 11-13.

Hypoxia-related tissue stress has been proposed as a relevant feature of the prostatic microenvironment and may contribute to pathological remodeling in prostate diseases, including BPH 14. Clinical Doppler ultrasound studies have reported altered vascular resistance in men with BPH/LUTS, supporting a potential link between prostatic vascular dysfunction and local hypoxia-related stress 15. Hypoxia has been shown to induce a range of cellular responses—including oxidative stress, metabolic shifts, and alterations in gene expression—that may either promote or delay cellular senescence depending on the cellular context, duration, and severity of oxygen deprivation 15-18. For example, mild or transient hypoxia may delay senescence in certain stem-like or stromal cells, while sustained or severe hypoxia can drive cells into irreversible cell-cycle arrest. These context-dependent outcomes suggest a complex and tissue-specific interplay between hypoxia and senescence that remains incompletely understood.

A key mediator of hypoxic signaling, hypoxia-inducible factor-1α (HIF-1α), has been reported to be increased in human BPH or hyperplastic prostate tissues, and experimental studies suggest that HIF-1α may contribute to prostate hyperplasia under hypoxic or inflammatory conditions 19-23. Although previous studies have identified context-dependent links between hypoxia/HIF-related signaling and cellular senescence in different disease models, HIF-1α may exert either pro-senescent or anti-senescent effects depending on the cellular and pathological context 24-26,34-42,46. For instance, in endometrial cancer, HIF-1α promotes senescence by upregulating cell-cycle inhibitors such as p21 and enhancing pro-inflammatory signaling, thereby limiting uncontrolled tumor cell proliferation. In contrast, in renal cell carcinoma, HIF-1α contributes to the maintenance of metabolic reprogramming and cancer cell stemness, delaying senescence and potentially aiding immune evasion. In chronic obstructive pulmonary disease (COPD) 24-26, sustained hypoxia leads to elevated HIF-1α expression in airway epithelial cells, which promotes cellular senescence and SASP production, exacerbating chronic inflammation and disease progression.

Recent advances in single-cell RNA sequencing (scRNA-seq) and spatial transcriptomics have enabled high-resolution characterization of cell states across tissues. In this study, analysis of publicly available human scRNA-seq datasets (GSE226237 and GSE183676), human spatial transcriptomic data (GSE278936), and a mouse BPH scRNA-seq dataset (GSE212770) suggested that senescence-associated transcriptional features in BPH are preferentially enriched in the epithelial compartment, particularly among luminal epithelial cells or Luminal-dominant spatial spots, and are accompanied by elevated hypoxia/HIF-1α pathway activity. Complementary reanalysis of LCM-based transcriptomic data from three BPH patients showed increased expression of genes associated with hypoxia signaling, cell-cycle arrest, senescence-associated chromatin remodeling, and canonical senescence markers in SA-β-gal-positive epithelial cells.

Collectively, these findings support a model in which hypoxia-associated HIF-1α/NF-κB signaling is linked to epithelial cellular senescence in BPH, particularly in the luminal epithelial compartment. This study refines the molecular characterization of SA-β-gal-positive epithelial cells in BPH and suggests that hypoxia-associated signaling may participate in disease progression through epithelial-stromal communication.

## Materials and Methods

### Patients

A total of 52 patients who underwent transurethral resection of the prostate (TURP) for benign prostatic hyperplasia (BPH) at Peking University First Hospital between July 2017 and October 2019 were included in this study. None of the patients had received medical treatment prior to surgery. Resected specimens were histologically confirmed as BPH by two experienced pathologists. Patients with urinary tract infection, prior catheterization, previous prostate-related surgery, prostatitis, or prostate cancer were excluded.

The study involving human tissue samples was approved by the Ethics Committee of Peking University First Hospital (Approval No. 2022-215). All procedures were conducted in accordance with institutional and national ethical guidelines. Written informed consent was obtained from all participants before sample collection.

### Senescence-associated β-galactosidase staining

Fresh-frozen prostate tissues from BPH patients were cryosectioned at a thickness of 8-10 μm and mounted onto standard glass slides. Senescence-associated β-galactosidase (SA-β-gal) staining was performed using a commercially available kit (C0602, Beyotime Biotechnology, China) according to the manufacturer's instructions. Briefly, tissue sections were fixed at room temperature for 10-15 min using the fixative solution provided in the kit, gently washed, and incubated overnight at 37 °C in X-gal staining buffer (pH 6.0) under non-CO₂ conditions. Senescent cells were visualized as blue deposits in the cytoplasm. Bright-field microscopy was used to capture images of the stained sections.

For *in vitro* analysis, BPH-1 and RWPE-1 cells were seeded into 35-mm culture dishes at a density of 600,000 cells per dish and treated under normoxic or hypoxic conditions (1% O₂) at 37 °C, with or without the HIF-1α inhibitor YC-1 (10 μM; S7958, Selleck, USA) or the NF-κB inhibitor PDTC (50 μM; S3633, Selleck, USA). Overexpression of *HIF1A* and knockdown of *HIF1A* or *RELA* were achieved by plasmid or siRNA transfection, as indicated. After 36 h of treatment, cells were subjected to senescence detection using a Cell Senescence β-Galactosidase Staining Kit (C0602, Beyotime Biotechnology, China), followed by incubation at 37 °C for 20 h. Stained cells were imaged using a bright-field microscope equipped with a 100× objective lens 27.

### Laser capture microdissection

Cryosections were prepared using a cryostat (CM1950, Leica, Germany) at -20 °C under RNase-free conditions. Work surfaces were pretreated with RNase Zap and 70% ethanol, followed by UV sterilization for 30 min. To ensure sufficient material for downstream RNA extraction and sequencing, appropriate regions of interest were identified for each biological replicate. Epithelial cells were captured using Arcturus PEN membrane RNase-free slides (Excilone, France). Captured cells were immediately collected into RNase-free tubes and stored at -20 °C until further processing. Target structures were visualized and microdissected under a light microscope using the XT® Arcturus Technologies Microdissection System (Excilone, France).

### RNA sequencing

SA-β-gal-positive and SA-β-gal-negative epithelial cells were isolated from freshly frozen prostate tissues obtained from three BPH patients who underwent TURP without prior medical treatment. Laser capture microdissection (LCM) was performed on adjacent serial sections to selectively collect target epithelial cells. Total RNA was extracted and subjected to transcriptome sequencing. Library preparation, quality control, and sequencing were conducted by the sequencing core. cDNA libraries were constructed using the SmartSeq HT Kit (Takara Bio) and the Illumina Nextera XT Kit (Illumina Inc.), followed by 2 × 75-bp paired-end sequencing on an Illumina NextSeq 500 platform using a 150-cycle run, with a target sequencing depth of approximately 40 million reads per sample.

After adapter trimming and quality filtering, clean reads were aligned to the human reference genome GRCh38/hg38. The RNA-seq data derived from LCM-captured SA-β-gal-positive and SA-β-gal-negative epithelial cells have been deposited in the Gene Expression Omnibus (GEO) database under accession number GSE297404. In the present study, this dataset was reanalyzed to address a distinct biological question focused on hypoxia-associated HIF-1α/NF-κB-linked epithelial senescence and related signaling pathways.

**Figure 1:**
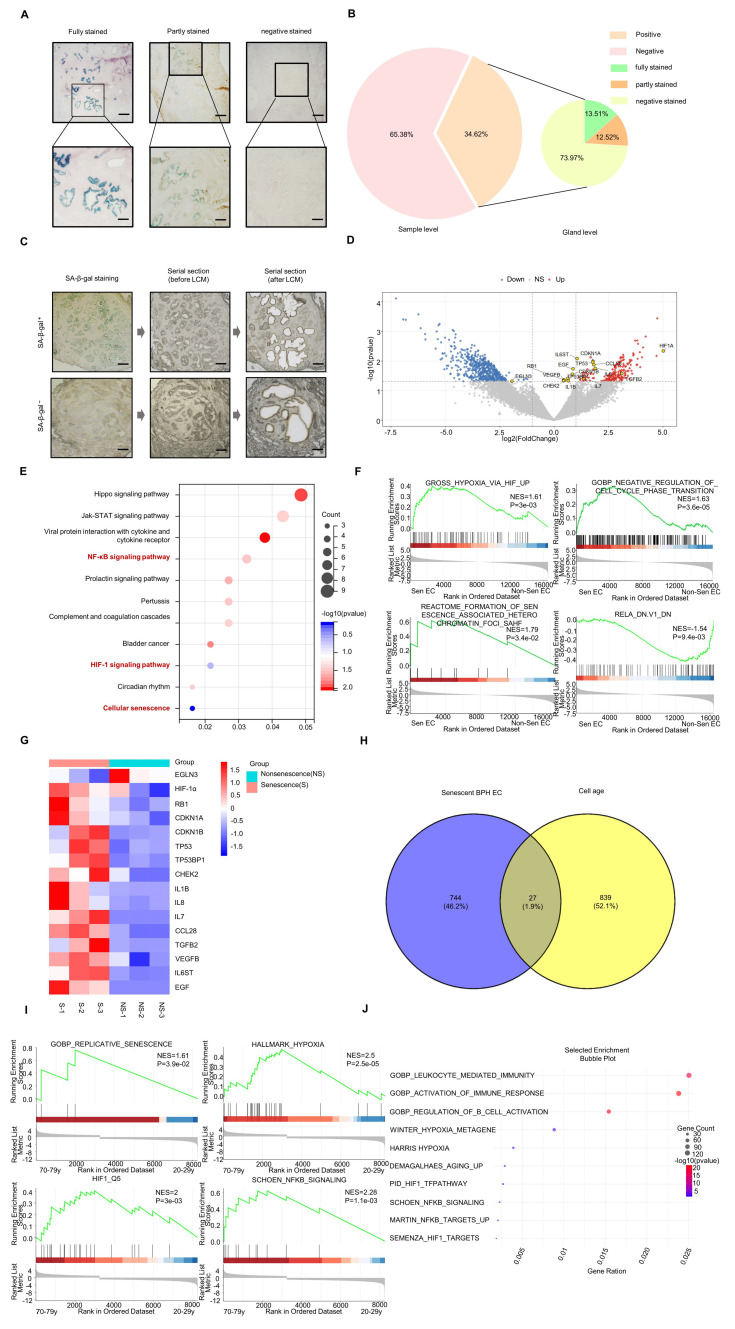
** Transcriptomic characterization of senescent cells in BPH tissues.** (A-B) Distribution of SA-β-gal staining in human BPH tissues. The 34.62% value represents the patient/specimen-level positivity rate, whereas the 13.51%, 12.52%, and 73.97% values represent gland-level staining patterns within the 18 SA-β-gal-positive specimens. Scale bars = 250 μm in the upper panels and 100 μm in the lower panels. (C) Laser capture microdissection (LCM) was used to isolate SA-β-gal-positive senescent epithelial cells from BPH tissues. Scale bar = 250 μm. (D) Volcano plot showing 771 differentially expressed genes (DEGs) between SA-β-gal-positive senescent epithelial cells and SA-β-gal-negative non-senescent glandular epithelial cells. Upregulated genes are indicated by red triangles, and downregulated genes are indicated by green triangles. (E) KEGG over-representation analysis of DEGs. Hypoxia-, NF-κB-, and senescence-related pathways are highlighted. (F) Representative GSEA plots of ranked DEGs comparing SA-β-gal-positive and SA-β-gal-negative epithelial cells. The displayed MSigDB gene sets/signatures include GROSS_HYPOXIA_VIA_HIF1A_UP, GOBP_NEGATIVE_REGULATION_OF_CELL_CYCLE_PHASE_TRANSITION, REACTOME_FORMATION_OF_SENESCENCE_ASSOCIATED_HETEROCHROMATIN_FOCI_SAHF, and RELA_DN.V1_DN. The green curve indicates the running enrichment score, and black vertical lines indicate the positions of gene-set members in the ranked list. (G) Transcriptomic analysis showing increased expression of representative genes associated with hypoxia signaling, senescence, and SASP-related programs in senescent epithelial cells, including *HIF1A*, *CDKN1A* encoding p21, *CDKN1B* encoding p27, *RB1* encoding Rb, and selected SASP-associated genes. In contrast, *EGLN3*, which encodes a prolyl hydroxylase involved in negative regulation of hypoxia signaling, was decreased. (H) Venn diagram showing the overlap between DEGs identified in senescent BPH epithelial cells and senescence-associated genes in the CellAge database. (I) Representative GSEA plots from the ADEIP prostate dataset comparing the older group aged 70-79 years with the younger group aged 20-29 years, showing enrichment of selected MSigDB gene sets/signatures related to replicative senescence, hypoxia/HIF-1 activity, and NF-κB signaling. (J) Bubble plot summarizing selected enriched gene sets/signatures from multiple MSigDB collections in the ADEIP comparison, including GO biological process terms, Hallmark hypoxia, and curated HIF-1- and NF-κB-related signatures. Panels C-G are based on reanalysis of the previously generated LCM RNA-seq dataset GSE297404, which was also reported in our recently published study on CXCL13-mediated CD4+ T-cell immune surveillance in BPH 48. The present analysis focuses on hypoxia/HIF-1α/NF-κB-linked epithelial senescence rather than immune-surveillance mechanisms.

For pathway analysis of the LCM-derived bulk RNA-seq data, over-representation analysis (ORA) was first performed on differentially expressed genes using the KEGG pathway database to identify enriched pathways, including HIF-1 signaling, NF-κB signaling, and cellular senescence-related pathways. In addition, gene set enrichment analysis (GSEA) was performed on the ranked gene list using selected gene sets/signatures from MSigDB. For the analyses presented in Fig. 1F, representative gene sets/signatures included GROSS_HYPOXIA_VIA_HIF1A_UP, GOBP_NEGATIVE_REGULATION_OF_CELL_CYCLE_PHASE_TRANSITION, REACTOME_FORMATION_OF_SENESCENCE_ASSOCIATED_HETEROCHROMATIN_FOCI_SAHF, and RELA_DN.V1_DN. These analyses were used to evaluate the coordinated enrichment of hypoxia/HIF-1α-, cell-cycle arrest-, senescence-associated chromatin/SAHF-, and NF-κB-related transcriptional programs in SA-β-gal-positive epithelial cells.

### Spatial transcriptomics analysis of human BPH prostate

Spatial transcriptomic data were obtained from GEO accession GSE278936, a prostate single-cell and spatial transcriptomics resource generated by Kiviaho *et al*.; the present analysis was restricted to the four specimens annotated as BPH 47. In the present study, we restricted our analysis to the four BPH specimens from the discovery cohort and downloaded the processed gene-by-spot count matrices, spot metadata, and corresponding hematoxylin and eosin (H&E) images from GEO. Because these data were generated in the original study, tissue processing, library construction, imaging, and sequencing were not performed by our group.

The 10x Genomics Visium platform provides spot-level spatial transcriptomic resolution rather than true single-cell resolution. Each capture area contains approximately 5,000 barcoded spots, with each spot measuring approximately 55 μm in diameter and a center-to-center distance of 100 μm between adjacent spots. Therefore, each spot may contain transcripts contributed by multiple neighboring cells. Accordingly, spatial spot labels in this study should be interpreted as the dominant transcriptional identity or cell state of a given location rather than as pure single-cell annotations.

Raw read processing and alignment to the human reference genome GRCh38/hg38 were performed in the original study. In our analysis, we started from the processed count matrices. Spots that did not overlap with tissue or failed basic quality control in the original dataset were excluded. To further ensure data quality, we removed spots with very low RNA content, extreme outlier values for detected genes or UMI counts, or an abnormally high fraction of mitochondrial reads using standard quality-control filters. The remaining 10,675 high-quality spots from the four BPH samples were used for downstream analyses.

Count data were normalized and variance-stabilized at the spot level, followed by scaling and principal component analysis (PCA). For each sample, we constructed a shared nearest-neighbor graph based on the top principal components and performed graph-based clustering. The resulting clusters were visualized using Uniform Manifold Approximation and Projection (UMAP). These per-sample analyses were used to assess intra-sample transcriptional heterogeneity.

To define cell types within the spatial data, we integrated the four BPH samples with our reference single-cell RNA-sequencing datasets, GSE226237 and GSE183676, derived from the human prostate transitional zone. A reference-based label-transfer approach was used to map single-cell-defined epithelial, stromal, and immune cell states onto spatial spots. Briefly, a cell-state reference was built from scRNA-seq data after batch correction and clustering, and anchor-based integration and label transfer were performed to infer the most likely dominant cell-state identity of each spot. These predicted labels were further curated using the expression patterns of canonical marker genes: *KRT8*, *KLK3*, and *ACPP* for Luminal cells; *ACTA2*, *MYH11*, and *TAGLN* for Smooth_muscle cells; *COL1A1*, *DCN*, and *PDGFRA* for Fibroblast_Myofib cells; *MZB1*, *JCHAIN*, and *IGKC* for Plasma cells; *SCGB1A1*, *PIGR*, and *KRT7* for Club cells; and *CD3D*, *CD4*, and *CD19* for T+B cells. The final cell-type assignments were overlaid on H&E images to visualize the spatial localization of each lineage.

For functional state analysis, we applied UCell gene-set scoring at the spot level using curated senescence- and hypoxia-related signatures. Senescence-associated transcriptional scores were calculated using the Gene Ontology term GOBP_POSITIVE_REGULATION_OF_CELLULAR_SENESCENCE, and hypoxia-response scores were calculated using REACTOME_CELLULAR_RESPONSE_TO_HYPOXIA. Because GOBP_POSITIVE_REGULATION_OF_CELLULAR_SENESCENCE represents genes involved in the positive regulation of cellular senescence rather than a definitive senescent-cell identity, the resulting UCell scores were interpreted as relative senescence-associated transcriptional activity rather than direct evidence that individual spots contained fully senescent cells. In addition, selected hypoxia- and senescence-related gene sets from MSigDB, including MENSE_HYPOXIA_UP and WEINMANN_ADAPTATION_TO_HYPOXIA_DN, were used in gene-set enrichment analyses. UCell scores were compared across major transferred spot identities/cell states using violin plots and spatial feature maps to identify spot populations with preferential activation of senescence-associated or hypoxia-response programs.

To further characterize senescence-associated transcriptional features within the luminal epithelial compartment, we restricted the analysis to Luminal-dominant spots and stratified them into senescence-score-high and senescence-score-low groups based on the median UCell score for GOBP_POSITIVE_REGULATION_OF_CELLULAR_SENESCENCE. These groups were used to compare relative senescence-associated pathway activity and should not be interpreted as definitive senescent versus non-senescent cell populations. For each group, spot-level counts were aggregated to generate pseudobulk expression profiles. Differential expression analysis between the two Luminal-dominant spot groups was performed using standard negative binomial models, yielding log₂ fold changes and adjusted *P* values. Ranked gene lists were then subjected to GSEA focusing on senescence-, proliferation-, hypoxia-, and NF-κB-related gene sets. ORA was also conducted on upregulated genes to identify enriched Gene Ontology Biological Process terms. These analyses were used to define pathway-level changes associated with spatially localized senescence-associated and hypoxia-related transcriptional programs in Luminal-dominant spots within BPH prostate tissue.

### CellAge and aging-related disease expression and immune profiling database analysis

To investigate aging-associated transcriptional programs relevant to prostatic epithelial senescence, we analyzed publicly available data from the CellAge database and the Aging-related Disease Expression and Immune Profiling (ADEIP) platform. The CellAge database was used to identify overlap between our differentially expressed genes and known senescence-associated genes. For ADEIP-based analyses, transcriptomic data from prostate tissues of elderly male donors aged 70-79 years and young male donors aged 20-29 years were compared. GSEA and enrichment visualization were performed using selected gene sets/signatures from multiple MSigDB collections, including GO Biological Process, Hallmark, and curated HIF-1- and NF-κB-related signatures. Representative gene sets/signatures used in Fig. 1I-J included GOBP_REPLICATIVE_SENESCENCE, HALLMARK_HYPOXIA, HIF1_Q5, SCHOEN_NFKB_SIGNALING, PID_HIF1_TFPATHWAY, MARTIN_NFKB_TARGETS_UP, and SEMENZA_HIF1_TARGETS. These analyses were used to assess enrichment of aging-, hypoxia/HIF-1-, and NF-κB-related transcriptional programs in the older group.

### scRNA-seq data acquisition and processing

scRNA-seq data were acquired for both human and mouse prostate tissues under BPH conditions. For human analysis, scRNA-seq datasets were obtained from GEO accessions GSE226237 and GSE183676, comprising six and five human BPH samples, respectively. For the mouse model, scRNA-seq data were retrieved from GSE212770, consisting of prostate tissues from ICR male mice subjected to castration followed by testosterone-induced BPH modeling. Raw sequencing data from all datasets were processed using the Cell Ranger pipeline v3.1.0 to generate standardized gene-expression matrices for downstream analysis.

### Data integration and quality control

For the human dataset, scRNA-seq data from 11 BPH patients were integrated using the Seurat R package v4.0. Cells expressing fewer than 200 genes or exhibiting more than 5% mitochondrial gene content were excluded from further analysis. Batch effects were corrected using canonical correlation analysis (CCA), resulting in a high-quality dataset comprising 41,547 cells.

For the mouse dataset, the same quality-control and integration procedures were applied, yielding 7,316 high-quality cells. Dimensionality reduction was performed using UMAP. Cell clustering was conducted using the Seurat FindClusters function, and cell types were annotated based on established canonical marker genes.

### Cell-type annotation

Cell types were identified by unsupervised clustering followed by annotation based on canonical marker genes.

In the human dataset, prostate cells were grouped into six major lineages: myeloid cells, B cells, T cells, natural killer (NK) cells, epithelial cells, and stromal cells. Marker genes used for annotation included *TPSAB1* for mast cells; *DCN*, *ACTA2*, and *COL1A2* for stromal cells; *KLK3* and *ACPP* for epithelial cells; *C1QA* and *C1QB* for myeloid cells; *CD79A* and *CD79B* for B cells; and *KLRD1* and *NKG7* for NK cells. T cells were identified by the expression of *CD3D*, *CD4*, and *CD8A*.

In the mouse dataset, unsupervised clustering identified nine major cell types: T/NK cells, B cells, myeloid cells, epithelial cells, mast cells, myofibroblasts, fibroblasts, smooth muscle cells, and endothelial cells. Representative marker genes included *Il2rb* and *Nkg7* for T/NK cells; *Cd19* and *Pax5* for B cells; *Mpeg1* and *Clec4a2* for myeloid cells; *Krt18*, *Krt8*, *Krt5*, and *Msmb* for epithelial cells; and *Col1a1* for fibroblasts.

### Epithelial cell subtype identification

Human epithelial cells (10,727 cells) were further classified into four subtypes: Luminal, Basal, Hillock, and Club cells. This classification was based on marker genes including *AR*, *KLK4*, *KLK3*, *KLK2*, and *ACPP* for Luminal cells; *TP63*, *KRT14*, and *KRT5* for Basal cells; *S100A14*, *S100A16*, and *KRT19* for Hillock cells; and *TACSTD2*, *SCGB1A3*, *WFDC2*, *LCN2*, and *MMP7* for Club cells.

Mouse epithelial cells (2,653 cells, 35.7% of total cells) were classified into three subtypes: Luminal cells (2,159 cells, 29.5%), Basal cells (376 cells, 5.1%), and rare epithelial cells (118 cells, 1.1%). This classification was based on marker genes including *Krt18* and *Krt8* for Luminal cells, *Krt5* and *Krt15* for Basal cells, and *Msmb* and *Gsdma* for rare epithelial cells.

### Hypoxia/HIF-1α pathway scoring

For both human and mouse epithelial cells, hypoxia/HIF-1α-related pathway activity was assessed using the MSigDB gene set GROSS_HYPOXIA_VIA_HIF1A_UP. Gene Set Variation Analysis (GSVA) was performed at the single-cell level, and each epithelial cell was assigned a GSVA score. Epithelial cells were then stratified into *HIF-1*α-pathway-high and *HIF-1*α-pathway-low groups using the median GSVA score as the cutoff. Thus, this grouping reflects pathway-level hypoxia/HIF-1α activity rather than single-gene *HIF1A* expression.

### Differential gene expression and pathway enrichment

Differential expression analysis between *HIF-1*α-pathway-high and *HIF-1*α-pathway-low epithelial cells was performed using the Seurat FindMarkers function. For human epithelial cells, 433 differentially expressed genes were identified, with 193 genes upregulated and 240 genes downregulated in the *HIF-1*α-pathway-high group. GSEA was conducted to identify pathways enriched in the HIF-1α-pathway-high group. Key enriched pathways included GROSS_HYPOXIA_VIA_HIF1A_UP, FRIDMAN_SENESCENCE_UP, HALLMARK_TNFA_SIGNALING_VIA_NFKB, and REACTOME_ONCOGENE_INDUCED_SENESCENCE, highlighting the roles of hypoxia, senescence, and NF-κB signaling in BPH pathophysiology.

### Immunohistochemical staining

To evaluate the relationship between HIF-1α expression and prostate epithelial cellular senescence in human BPH tissues, immunohistochemical (IHC) staining was performed on paraffin-embedded human prostate tissue sections.

For representative image presentation, corresponding regions from serial paraffin sections of the same specimen were selected to compare HIF-1α and senescence-marker staining. Prostate specimens obtained from patients undergoing TURP were fixed in 4% formalin buffer overnight at 4 °C, dehydrated, and embedded in paraffin. Sections were cut at a thickness of 5 μm. After deparaffinization and rehydration, endogenous peroxidase activity was blocked using 3% hydrogen peroxide. Antigen retrieval was performed where applicable. Sections were incubated overnight at 4 °C with the following primary antibodies:

Anti-HIF-1α (sc-10372, Santa Cruz Biotechnology, USA; 1:250 dilution), Anti-p21 (#2947, Cell Signaling Technology, USA; 1:800 dilution), Anti-p27 (D69C12, Cell Signaling Technology, USA; 1:2000 dilution), Anti-Rb (#9309, Cell Signaling Technology, USA; 1:2000 dilution).

After washing, biotinylated secondary antibodies were applied for 30 min at room temperature, followed by incubation with streptavidin-peroxidase conjugate. The signal was visualized using a DAB substrate kit, and hematoxylin was used for nuclear counterstaining. Two independent urologic pathologists performed blinded microscopic evaluations. The integrated optical density (IOD) of positively stained areas was quantified using Image-Pro Plus 6.0 software (Media Cybernetics, Rockville, MD, USA).

### Cell lines

The BPH-1 cell line, a human benign prostatic hyperplasia epithelial cell line, was obtained from Wuhan Pricella Biotechnology Co., Ltd. (Catalog No. CL-0865; Batch No. L861116000603) in November 2023. The RRID for this cell line is CVCL_1091, as registered in the Cellosaurus database. The RWPE-1 cell line, a STR-verified human normal prostate epithelial cell line, was also purchased from Wuhan Pricella Biotechnology Co., Ltd. (Catalog No. CL-0200) in November 2023. The RRID for RWPE-1 is CVCL_3791. The human prostate stromal cell line WPMY-1 was also obtained from Wuhan Pricella Biotechnology Co., Ltd. (Catalog No. CL-0467), and its RRID is CVCL_3814. Primary human prostate stromal cells (PrSCs) were isolated from fresh prostate tissues obtained from patients undergoing TURP for BPH, as described below.

RWPE-1 cells were cultured in complete Keratinocyte Serum-Free Medium (KSFM; Gibco, USA) supplemented with bovine pituitary extract and recombinant human epidermal growth factor (EGF). BPH-1 cells were maintained in RPMI-1640 medium (Gibco, USA) supplemented with 10% fetal bovine serum (FBS), 100 U/mL penicillin, and 100 μg/mL streptomycin. WPMY-1 cells were maintained in DMEM supplemented with 5% FBS and 1% penicillin/streptomycin. Primary PrSCs were cultured in DMEM/F12 medium at a 1:1 ratio, supplemented with 10% FBS, 1% penicillin/streptomycin, 1 μg/mL EGF, and 1 μg/mL bFGF. All cells were incubated at 37 °C in a humidified atmosphere containing 5% CO₂. For hypoxia conditioning, BPH-1 and RWPE-1 cells were cultured under hypoxic conditions (1% O₂) as indicated. BPH-1 and RWPE-1 cell lines were routinely tested and confirmed to be free of contamination, and RWPE-1 cells were STR-verified to ensure authenticity.

### Isolation and culture of primary human prostate stromal cells

Fresh prostate tissues were collected immediately after TURP from BPH patients under sterile conditions and placed in PBS containing 1% penicillin/streptomycin. The tissues were washed three times with PBS and minced into approximately 1-mm³ fragments. Tissue digestion was performed in collagenase digestion buffer containing PBS supplemented with 10 mM HEPES, BSA, CaCl₂, and type I collagenase (10 mg/mL), followed by incubation at 37 °C with gentle shaking at 75 rpm for 2-4 h. During digestion, samples were gently mixed every 30 min.

After digestion, the tissue suspension was centrifuged, resuspended in culture medium, and plated in 10-cm culture dishes. The culture medium consisted of DMEM/F12 at a 1:1 ratio supplemented with 10% FBS, 1% penicillin/streptomycin, 1 μg/mL EGF, and 1 μg/mL bFGF. Cells were cultured at 37 °C in a humidified atmosphere containing 5% CO₂. The medium was changed the following day to remove unattached tissue fragments. When sufficient stromal-cell outgrowth was observed, cells were passaged and expanded. Passage 2 to passage 10 primary stromal cells were used for subsequent experiments.

### Preparation of conditioned medium and stromal-cell stimulation

BPH-1 and RWPE-1 cells were divided into three groups: NC, Hypoxia, and Hypoxia + si*HIF1A*. Knockdown of *HIF1A* was achieved by siRNA transfection as described below. After the indicated treatments, epithelial cells were washed three times with PBS and incubated in fresh complete medium for an additional 24 h to generate conditioned medium (CM). Culture supernatants were then collected and centrifuged to remove cell debris. CM derived from the three groups was designated as NC-CM, Hypoxia-CM, and Hypoxia + si*HIF1A*-CM, respectively.

For stromal-cell stimulation experiments, WPMY-1 cells and PrSCs were seeded in the indicated plates and allowed to attach overnight. The original culture medium was then replaced with epithelial-derived conditioned medium mixed 1:1 with RPMI-1640 medium. Stromal cells were cultured in the corresponding mixed CM for subsequent CCK-8, EdU, and cell-cycle analyses.

### Cell counting kit-8 assay

Cell proliferation was assessed using the Cell Counting Kit-8 (CCK-8; Beyotime Institute of Biotechnology, China) according to the manufacturer's instructions. Briefly, 3,000 cells per well were seeded in 96-well flat-bottom plates and cultured at 37 °C under normoxic or hypoxic conditions (1% O₂), with or without YC-1 (10 μM; S7958, Selleck, USA) or PDTC (50 μM; S3633, Selleck, USA). Cell viability was measured every 12 h for a total of 2 or 4 days.

At each time point, 10 μL of CCK-8 reagent was diluted in 100 μL of complete medium and added to each well. After incubation at 37 °C for 2 h, absorbance was measured at 450 nm using a microplate reader (Bio-Rad). Each experiment was performed in triplicate and independently repeated at least three times.

Stromal-cell proliferation was also assessed using the CCK-8 assay. Briefly, WPMY-1 cells and PrSCs were seeded in 96-well flat-bottom plates at densities of 3,000 cells/well and 1,500 cells/well, respectively, and allowed to attach overnight. The cells were then cultured with the indicated epithelial-derived conditioned medium mixed 1:1 with RPMI-1640 medium for 5 days. Cell viability was measured on days 0, 1, 3, and 5. At each time point, the culture medium was removed, and 100 μL of serum-free, phenol red-free RPMI-1640 medium containing CCK-8 reagent at a 10:1 ratio was added to each well. After incubation at 37 °C for 2 h, absorbance was measured at 450 nm using a microplate reader. Each experiment was performed in triplicate and independently repeated at least three times.

### Flow cytometry

BPH-1 and RWPE-1 cells were seeded into six-well plates at a density of 300,000 cells per well and cultured at 37 °C under normoxic or hypoxic conditions (1% O₂), with or without YC-1 (10 μM; S7958, Selleck, USA) or PDTC (50 μM; S3633, Selleck, USA). Overexpression of *HIF1A* and knockdown of *HIF1A* or *RELA* were achieved by plasmid or siRNA transfection, as indicated. Cells were harvested every 12 h for 2 days.

For fixation, 5 mL of precooled 70% ethanol was added to each sample, and cells were stored overnight at 4 °C. Fixed cells were washed and incubated with RNase (10 mg/mL in 1 M Tris-HCl, pH 7.4) at 37 °C for 30 min. Subsequently, propidium iodide (PI) solution was added at 450 μL per sample, and cells were incubated on ice for 30 min in the dark.

Samples were analyzed using a flow cytometer, and DNA content was assessed using CellQuest software (BD Biosciences). Cell-cycle phase distribution, including G0/G1, S, and G2/M phases, was determined using ModFit LT software.

For stromal-cell cycle analysis, WPMY-1 cells and PrSCs were seeded into six-well plates at densities of 3 × 10⁵ cells/well and 1.5 × 10⁵ cells/well, respectively, and cultured with the indicated epithelial-derived conditioned medium mixed 1:1 with RPMI-1640 medium for 5 days. Cells were then washed three times with precooled PBS, harvested by trypsinization, and centrifuged. After resuspension in PBS and a second centrifugation step, cells were fixed in 75% ethanol overnight at 4 °C. Fixed cells were washed twice with PBS, resuspended in RNase A solution, and incubated at room temperature for 30 min. PI was then added at a final concentration of 50 μg/mL, and samples were incubated in the dark for 30 min. Cell-cycle distribution was analyzed using a flow cytometer (BD, USA), and data were analyzed using FlowJo software. The percentage of cells in S phase was used as the major indicator of stromal proliferative activity.

### Western blot analysis

Cells were lysed using RIPA lysis buffer supplemented with 1 mM PMSF, 0.5% phosphatase inhibitor, and 1% protease inhibitor (KGP250, Keygen Biotech, China). A total of 25 μg of protein per sample was loaded onto SDS-PAGE gels for electrophoresis, followed by transfer onto nitrocellulose membranes.

The membranes were blocked in Tris-buffered saline (TBS) containing 5% non-fat milk for 1 h at room temperature and then incubated overnight at 4 °C with the following primary antibodies diluted in TBS-T:

Anti-p21 (#13110, Cell Signaling Technology, USA; 1:1000), Anti-p27 (D69C12, Cell Signaling Technology, USA; 1:1000), Anti-Rb (#9309, Cell Signaling Technology, USA; 1:1000), Anti-p65 (#12282, Cell Signaling Technology, USA; 1:1000), Anti-phospho-p65 (Ser536) (#2527, Cell Signaling Technology, USA; 1:1000), Anti-IκB-α (L35A5, Cell Signaling Technology, USA; 1:1000), Anti-β-tubulin (60004-1-Ig, Proteintech, USA; 1:2000).

After three washes with TBS-T, the membranes were incubated with appropriate HRP-conjugated secondary antibodies (Invitrogen) for 1 h at room temperature. Protein bands were visualized using enhanced chemiluminescence reagents (P90719, Millipore, MA, USA).

### qRT-PCR validation of* HIF1A* overexpression and *HIF1A/RELA* knockdown

Overexpression of *HIF1A* was achieved by transient transfection of expression plasmids into prostate epithelial cells, whereas knockdown of *HIF1A* and *RELA* was achieved using siRNAs, as indicated in each experiment. Cells transfected with the corresponding empty vector were used as negative controls. After transfection, cells were collected at the indicated time points for qRT-PCR, western blotting, cell-cycle analysis, EdU assay, and SA-β-gal staining. Overexpression efficiency was validated by qRT-PCR and western blot analysis.

Small interfering RNAs (siRNAs) were used for transient knockdown of *HIF1A* and *RELA* in prostate epithelial cells. The sequences used for *HIF1A* knockdown were as follows:*HIF1A*-siRNA-1: sense 5′-CCAGCAGACUCAAAUACAATT-3′, antisense 5′-UUGUAUUUGAGUCUGCUGGTT-3′;*HIF1A*-siRNA-2: sense 5′-GCUGGAGACACAAUCAUAUTT-3′, antisense 5′-AUAUGAUUGUGUCUCCAGCTT-3′;*HIF1A*-siRNA-3: sense 5′-GCCGAGGAAGAACUAUGAATT-3′, antisense 5′-UUCAUAGUUCUUCCUCGGCTT-3′.The sequences used for *RELA* knockdown were as follows:*RELA*-siRNA-1: sense 5′-CCAUCAACUAUGAUGAGUUTT-3′, antisense 5′-AACUCAUCAUAGUUGAUGGTT-3′;*RELA*-siRNA-2: sense 5′-CUUCCAAGUUCCUAUAGAATT-3′, antisense 5′-UUCUAUAGGAACUUGGAAGTT-3′;*RELA*-siRNA-3: sense 5′-GGACAUAUGAGACCUUCAATT-3′, antisense 5′-UUGAAGGUCUCAUAUGUCCTT-3′.Cells transfected with scrambled control siRNA were used as negative controls. Knockdown efficiency was validated by qRT-PCR and western blot analysis.

Total RNA was extracted and reverse-transcribed into cDNA. Quantitative real-time PCR was performed using GoTaq qPCR Master Mix (A6001; Promega, Madison, WI, USA) on an Applied Biosystems 7500 Fast Real-Time PCR System according to the manufacturer's instructions. Relative mRNA expression levels were normalized to *GAPDH* and calculated using the comparative Ct method.

### EdU assay

BPH-1 and RWPE-1 cells were seeded into six-well plates at a density of 300,000 cells per well and cultured under normoxic or hypoxic conditions (1% O₂), with or without YC-1 (10 μM; S7958, Selleck, USA) or PDTC (50 μM; S3633, Selleck, USA), at 37 °C. Overexpression of *HIF1A* and knockdown of *HIF1A* or *RELA* were achieved by plasmid or siRNA transfection, as indicated. At 36 h post-treatment, cells were incubated with the Cell-Light EdU Apollo567 *In Vitro* Kit (100T; Cat. No. C10310-1, RiboBio, China) in a humidified incubator at 37 °C with 5% CO₂ for 20 h. EdU-labeled cells were visualized using a fluorescence microscope equipped with a 100× objective lens 27.

WPMY-1 cells and PrSCs were cultured with the indicated conditioned media and assessed for DNA synthesis using the Cell-Light EdU Apollo567 *In Vitro* Kit according to the manufacturer's instructions. Briefly, stromal cells were seeded in the indicated plates, allowed to attach overnight, and then incubated with the corresponding conditioned media. After the indicated treatment period, cells were incubated with EdU working solution, fixed, stained, and imaged under a fluorescence microscope equipped with a 100× objective lens. The percentage of EdU-positive cells was quantified from representative microscopic fields and used to evaluate the proliferative response of stromal cells after conditioned-medium stimulation.

### Cytokine antibody array assay

For exploratory profiling of secreted cytokines associated with epithelial senescence, BPH-1 cells were seeded into 35-mm culture dishes at a density of 600,000 cells per dish and maintained under normoxic or hypoxic conditions (1% O₂), with or without YC-1 (10 μM; S7958, Selleck, USA), at 37 °C for the indicated duration. At 36 h post-treatment, culture supernatants were collected and analyzed using a Human Cytokine Antibody Array (membrane-based, 42 targets; Cat. No. ab133997, Abcam, USA). Signal detection was performed according to the manufacturer's instructions, and membranes were visualized using a chemiluminescence imaging system. Relative signal intensity was quantified by densitometric analysis.

### ELISA assay for conditioned medium

To validate representative SASP-associated factors in conditioned media from hypoxia-treated prostate epithelial cells, BPH-1 and RWPE-1 cells were divided into three groups: NC, Hypoxia, and Hypoxia + si*HIF1A*. Knockdown of *HIF1A* was achieved by siRNA transfection as described above. After the indicated treatments, epithelial cells were washed three times with PBS and incubated in fresh complete medium for an additional 24 h to generate conditioned medium. Culture supernatants were then collected and centrifuged to remove cell debris.

The concentrations of IL-1α, IL-6, IL-8, IL-15, and CXCL12/SDF1 in the conditioned media were determined using individual human ELISA kits, including the Human IL-1 alpha ELISA Kit (Abcam, ab100560), Human IL-6 ELISA Kit (Abcam, ab178013), Human IL-8 ELISA Kit (Abcam, ab100575), Human IL-15 ELISA Kit (Abcam, ab100554), and Human SDF1 ELISA Kit (Abcam, ab100637), according to the manufacturers' instructions. Briefly, standards and samples were added to antibody-precoated 96-well plates and incubated as instructed by the manufacturer. After sequential incubation with the detection antibody and enzyme conjugate, substrate solution was added for color development, and the reaction was terminated with stop solution. Absorbance was measured at 450 nm using a microplate reader, and cytokine concentrations were calculated according to standard curves. Relative cytokine abundance was compared among the three groups.

### Testosterone propionate-induced BPH-like prostatic hyperplasia mouse model and pharmacological treatment

Male C57BL/6J mice aged 8-10 weeks were obtained from GemPharmatech Co., Ltd. (Nanjing, China) and maintained under specific pathogen-free conditions with free access to food and water. All animal experimental procedures were approved by the Animal Ethics Committee of Peking University First Hospital (Approval No. 2024-174) and were conducted in accordance with institutional and national guidelines for the care and use of laboratory animals.

A total of 20 mice were randomly divided into four groups, with five mice in each group: the Sham + Vehicle group, BPH + Vehicle group, BPH + PX-478 group, and BPH + BMS-345541 group. Mice in the Sham + Vehicle group underwent sham surgery, consisting of skin incision and closure without orchiectomy. Mice in the other three groups were subjected to bilateral orchiectomy. After a 1-week recovery period, mice in the BPH groups received daily subcutaneous injection of testosterone propionate (TP; T7260, Solarbio, Beijing, China) at 5 mg/kg/day for 42 consecutive days to induce BPH-like prostatic hyperplasia. TP was dissolved in corn oil.

For pharmacological intervention, mice in the BPH + PX-478 group received PX-478 (HY-10231, MedChemExpress, USA) at 20 mg/kg/day, whereas mice in the BPH + BMS-345541 group received BMS-345541 (HY-10519, MedChemExpress, USA) at 30 mg/kg/day. PX-478 and BMS-345541 were administered by oral gavage once daily, 5 days per week, for 4 consecutive weeks, for a total of 20 administrations during the TP induction period. Vehicle-treated mice received the corresponding vehicle in parallel. Body weight was recorded once weekly throughout the experimental period.

At the endpoint, mice were weighed and euthanized. Blood samples were collected, and prostate tissues were carefully dissected and weighed. The prostate index was calculated as prostate wet weight divided by body weight and expressed as mg/g. Prostate tissues were divided for paraffin embedding and frozen storage for subsequent histological and immunohistochemical analyses.

### Histological staining and morphometric analysis

Prostate tissues were fixed in 4% paraformaldehyde, embedded in paraffin, and sectioned at 5 μm. Sections were stained with hematoxylin and eosin (H&E) according to standard protocols. Histological images were captured under identical microscope settings.

Morphometric analysis was performed using ImageJ software by an observer blinded to group allocation. For each mouse, five randomly selected fields from comparable prostate regions were analyzed. Epithelial thickness was measured as the perpendicular distance from the basement membrane to the luminal surface of the glandular epithelium. Mean glandular lumen area was quantified by manually outlining the luminal area of intact, non-truncated glands and was expressed as ×10^-3 mm²/gland. The average value from each mouse was used as one biological replicate.

### Immunohistochemistry and semiquantitative scoring of mouse prostate tissues

Serial paraffin sections of mouse prostate tissues were used for immunohistochemistry. After deparaffinization and rehydration, antigen retrieval was performed using citrate buffer (pH 6.0) in a microwave oven. Endogenous peroxidase activity was blocked with 3% hydrogen peroxide. Sections were then incubated overnight at 4 °C with primary antibodies against HIF-1α (sc-13515, Santa Cruz Biotechnology, USA; 1:250 dilution), phosphorylated p65 (Ser536; p-p65; #3033, Cell Signaling Technology, USA; 1:500 dilution), p21 (ab188224, Abcam, UK; 1:1000 dilution), p27 (26714-1-AP, Proteintech, USA; 1:300 dilution), Rb (ab181616, Abcam, UK; 1:1000 dilution), and IκB-α (L35A5, #4814, Cell Signaling Technology, USA; 1:500 dilution).

After washing, sections were incubated with HRP-conjugated secondary antibodies, and immunoreactive signals were visualized using DAB. Nuclei were counterstained with hematoxylin. Images were acquired under identical microscope settings.

IHC staining was evaluated primarily in the glandular epithelial compartment by observers blinded to group allocation. Staining intensity was semiquantitatively scored as follows: 0, negative; 1, weak; 2, moderate; and 3, strong. Five randomly selected fields from each mouse were scored. For statistical analysis, the mean score of the five fields from each mouse was used as one biological replicate. Field-level scores are shown in violin plots to visualize the distribution of staining intensity, whereas statistical comparisons were performed using mouse-level mean values.

### Statistical analysis

Data are presented as mean ± SD for *in vitro* experiments unless otherwise indicated. For animal experiments, data are presented as mean ± SEM. For *in vitro* experiments, n represents independent biological replicates. For animal experiments, n represents individual mice. For histological and IHC quantification, multiple fields were evaluated for each mouse, and the mean value from each mouse was used as one biological replicate. Field-level IHC scores are shown only to visualize score distributions, whereas statistical comparisons were performed using mouse-level mean values. For comparisons between two groups, two-tailed Student's t tests were used. For comparisons among multiple groups, one-way ANOVA followed by Tukey's multiple-comparison test was performed. Pearson correlation analysis was used for IHC staining intensity and positive-area correlations unless otherwise indicated. Spearman's rank correlation analysis was used for pathway-score correlation analyses in scRNA-seq and spatial transcriptomic datasets. Statistical analyses were conducted using GraphPad Prism 9.0. A value of P < 0.05 was considered statistically significant.

## Results

### Transcriptomic characterization of senescent cells in BPH tissues

Although the mechanisms of cellular senescence have been extensively studied *in vitro*, Dimri *et al*. first confirmed the presence of senescent cells *in vivo* in skin tissue in 1995 using senescence-associated β-galactosidase (SA-β-gal) staining 43. To investigate senescence-associated features in BPH tissues, we performed SA-β-gal staining on freshly frozen prostate tissue sections from patients with BPH. Positive SA-β-gal staining, visualized as blue-green precipitates, was observed in 34.62% of cases (18/52). SA-β-gal-positive cells were predominantly localized in the epithelial compartment, with minimal staining observed in stromal regions. Among the 18 SA-β-gal-positive specimens, gland-level staining patterns were heterogeneous: 13.51% of glands showed diffuse positivity, 12.52% showed partial positivity, whereas 73.97% showed no detectable SA-β-gal staining (Fig. 1A-B). Although staining intensity varied among individuals, it was generally consistent within the same gland (Fig. S1).

To further investigate hypoxia-associated transcriptional programs in SA-β-gal-positive epithelial cells, we reanalyzed the previously generated LCM RNA-seq dataset GSE297404 from SA-β-gal-positive and SA-β-gal-negative epithelial cells, which was also reported in our recently published immune-surveillance study 48. This dataset was used to compare the transcriptomic profiles of SA-β-gal-positive and SA-β-gal-negative epithelial cells (Fig. 1C). A total of 771 differentially expressed genes (DEGs) were identified, including 203 upregulated and 568 downregulated genes in SA-β-gal-positive epithelial cells compared with SA-β-gal-negative epithelial cells (Fig. 1D). KEGG over-representation analysis of these DEGs identified significant enrichment of multiple pathways, including HIF-1 signaling, NF-κB signaling, and cellular senescence (Fig. 1E). GSEA of the ranked gene list further supported enrichment of hypoxia/HIF-, cell-cycle arrest-, senescence-associated chromatin-, and NF-κB-related gene sets/signatures in SA-β-gal-positive epithelial cells, including GROSS_HYPOXIA_VIA_HIF1A_UP, GOBP_NEGATIVE_REGULATION_OF_CELL_CYCLE_PHASE_TRANSITION, REACTOME_FORMATION_OF_SENESCENCE_ASSOCIATED_HETEROCHROMATIN_FOCI_SAHF, and RELA_DN.V1_DN (Fig. 1F).Transcriptomic profiling revealed increased expression of the hypoxia-associated gene *HIF1A*; classical senescence-related genes, including *RB1*, *CDKN1A* encoding p21, *CDKN1B* encoding p27, *TP53*, *TP53BP1*, and *CHEK2*; and genes encoding multiple SASP-associated factors, such as *IL1B*, *IL7*, *IL8*, *CCL28*, *TGFB2*, and *EGF*. In contrast, *EGLN3*, which encodes a prolyl hydroxylase involved in negative regulation of hypoxia signaling, was significantly downregulated in SA-β-gal-positive epithelial cells (Fig. 1G).

To assess whether these SA-β-gal-associated DEGs overlapped with known senescence regulators, we cross-referenced the 771 DEGs with the CellAge database and identified 27 overlapping genes, including *HIF1A* (Fig. 1H). We further analyzed transcriptomic data from the ADEIP database by comparing prostate tissues from elderly male donors aged 70-79 years with those from young male donors aged 20-29 years. GSEA and enrichment analysis using selected gene sets/signatures from multiple MSigDB collections showed enrichment of aging-, hypoxia/HIF-1-, and NF-κB-related programs in the older group, including GOBP_REPLICATIVE_SENESCENCE, HALLMARK_HYPOXIA, HIF1_Q5, and SCHOEN_NFKB_SIGNALING (Fig. 1I-J).

### Single-cell transcriptomic analysis reveals coordinated hypoxia-, NF-κB-, and senescence-associated transcriptional programs in epithelial subtypes of BPH prostate

We performed single-cell RNA sequencing (scRNA-seq) analysis of human prostate tissues using publicly available datasets GSE226237 and GSE183676 from the GEO database. After quality control and batch correction, 41,547 high-quality cells were retained. The full single-cell atlas, including lineage annotation, marker-gene summaries, epithelial-cell stratification by hypoxia/HIF-1α pathway activity, and pathway-enrichment visualizations, is provided in Fig. S2.

Based on canonical marker genes, cells were annotated into six major lineages: epithelial cells, stromal cells, myeloid cells, B cells, T cells, and natural killer (NK) cells. Marker specificity is summarized primarily in the dot plot shown in Fig. S2C, whereas feature plots are provided as supportive visualizations in Fig. S2D. These analyses established the single-cell annotation framework for downstream evaluation of hypoxia-, NF-κB-, and senescence-associated transcriptional programs in epithelial cells.

Given the enrichment of hypoxia-, HIF-1-, NF-κB-, and senescence-related programs in LCM-captured senescent epithelial cells, we next examined whether similar transcriptional features were present at single-cell resolution. Epithelial cells were stratified into HIF-1α pathway-high and HIF-1α pathway-low groups using GSVA scores for the MSigDB gene set GROSS_HYPOXIA_VIA_HIF1A_UP. Differential expression and pathway-level analyses indicated coordinated enrichment of hypoxia-, NF-κB-, and senescence-related transcriptional programs in HIF-1α pathway-high epithelial cells. Summary enrichment results are shown in Fig. S2I, whereas representative GSEA plots are provided in Fig. S2H.

To further characterize epithelial subtypes, we extracted 10,727 epithelial cells for detailed analysis. After quality control and batch correction, these cells were clustered into four epithelial subtypes: luminal, basal, hillock, and club cells. Among these epithelial populations, luminal cells showed the highest hypoxia/HIF-1α pathway activity and accounted for the largest fraction of epithelial cells with elevated GSVA scores. Differential-expression and enrichment analyses within luminal cells further supported an association between increased hypoxia/HIF-1α pathway activity and senescence-related transcriptional programs, as detailed in Fig. S3.

In the corresponding DEG volcano plot, we labeled representative senescence-associated and pathway-related genes selected for cross-dataset comparison, including *ARG2*, *PAWR*, *TP53*, *HIF1A*, *NFKB1*, and *CDKN1A* (Fig. S3G). These gene-level annotations complemented the pathway-level GSEA results and facilitated comparison with the subsequent spatial transcriptomic analysis.

Because these single-cell analyses were based on pathway scoring, differential expression, and enrichment analysis, they should be interpreted as evidence of coordinated transcriptional states rather than direct proof of unidirectional causality. To further examine whether hypoxia-, NF-κB-, and senescence-associated transcriptional programs were coordinated at the pathway-score level, we performed pairwise correlation analyses using the same gene-set scoring framework applied in our scRNA-seq and spatial transcriptomic analyses. In epithelial cells from the human BPH scRNA-seq dataset, hypoxia/HIF-1α pathway scores were positively correlated with both NF-κB and senescence scores, and NF-κB scores were also positively correlated with senescence scores (Fig. S9A-C). Similarly, in the spatial transcriptomic dataset, spot-level hypoxia-response, NF-κB, and senescence scores showed positive pairwise associations, with the strongest association observed between NF-κB and senescence scores (Fig. S9D-F). Together, these results provide transcriptomic evidence linking hypoxia-associated signaling, NF-κB activation, and epithelial senescence in BPH, while functional causality was further examined using perturbation-based *in vitro* experiments.

### Spatial transcriptomic atlas of human BPH prostate

We next performed spatial transcriptomic analysis of human BPH tissues using the publicly available GEO dataset GSE278936. Four BPH specimens, GSM8557976_BPH_1, GSM8557977_BPH_2, GSM8557978_BPH_3, and GSM8557979_BPH_4, hereafter referred to as Sample 1-4, were included, yielding a total of 10,675 high-quality spots after quality control. The full spatial transcriptomic atlas, including per-sample clustering, cell-type annotation, integrated UMAP projections, H&E-overlaid spatial localization, and sample-wise compositional analyses, is provided in Figs. S4-S5.

These analyses established the spatial annotation framework for downstream evaluation of senescence- and hypoxia-associated transcriptional states in BPH tissue. Integration with the reference scRNA-seq dataset identified six major transferred dominant spot identities/cell states: Smooth_muscle-dominant spots, Luminal-dominant spots, Fibroblast_Myofib-dominant spots, Plasma cell-dominant spots, Club cell-dominant spots, and T+B cell-dominant spots. Across all spots, Smooth_muscle-dominant spots represented the largest annotated population, followed by Luminal-dominant spots, whereas fibroblast/myofibroblast- and immune-dominant spots formed smaller but biologically relevant compartments of the tissue microenvironment. H&E-overlaid spatial localization further showed that Smooth_muscle-dominant spots were predominantly distributed in stromal and periglandular regions, whereas Luminal-dominant spots were enriched along the glandular epithelium.

To further validate the accuracy of cell-type annotation, we examined the expression patterns of representative marker genes in each lineage. Fig. S6A-C shows violin plots for T+B cells (*CD3D*, *CD4*, *CD19*), Plasma cells (*MZB1*, *JCHAIN*, *IGKC*), and Club cells (*SCGB1A1*, *PIGR*, *KRT7*), whereas Fig. S7A-C presents marker expression in Luminal cells (*KRT8*, *KLK3*, *ACPP*), Smooth_muscle cells (*ACTA2*, *MYH11*, *TAGLN*), and Fibroblast_Myofib cells (*COL1A1*, *DCN*, *PDGFRA*). In each case, marker genes displayed high expression in the expected lineage and low background expression in other clusters, supporting the robustness of the spatial cell-type annotations.

Building on this spatial atlas, we next assessed senescence- and hypoxia-associated transcriptional features across major transferred spot identities/cell states, with a particular focus on Luminal-dominant spots. UCell scoring using GOBP_POSITIVE_REGULATION_OF_CELLULAR_SENESCENCE showed that Luminal-dominant spots exhibited the highest senescence-associated transcriptional scores among the major spatially annotated spot populations (Fig. S8A-H). Because this gene set reflects positive regulation of cellular senescence rather than a definitive senescent-cell identity, these scores were interpreted as relative senescence-associated transcriptional activity at the spot level.

To further characterize senescence-associated transcriptional features within the luminal epithelial compartment while reducing confounding from major cell-type heterogeneity, we restricted the analysis to Luminal-dominant spots. These spots were stratified into senescence-score-high and senescence-score-low groups according to the median UCell score. Compared with senescence-score-low Luminal-dominant spots, senescence-score-high Luminal-dominant spots showed upregulation of representative senescence-associated genes and coordinated enrichment of hypoxia-, hypoxia-adaptation-, and pro-inflammatory programs, whereas proliferation-related signatures were negatively enriched (Fig. S8I-V). These findings suggest that Luminal-dominant spatial spots represent a major spatial compartment associated with coordinated senescence- and hypoxia-related transcriptional programs in BPH prostate tissues, rather than directly identifying pure senescent luminal epithelial cells.

### Expression of HIF-1α and senescence markers in human BPH tissues

To explore the relationship between HIF-1α expression and epithelial cellular senescence in BPH tissues, immunohistochemical staining was performed on serial paraffin sections from 52 human BPH prostate samples. HIF-1α, p21, p27, and Rb were primarily expressed in the epithelial compartment, with minimal or no detectable staining in stromal cells.

**Figure 2:**
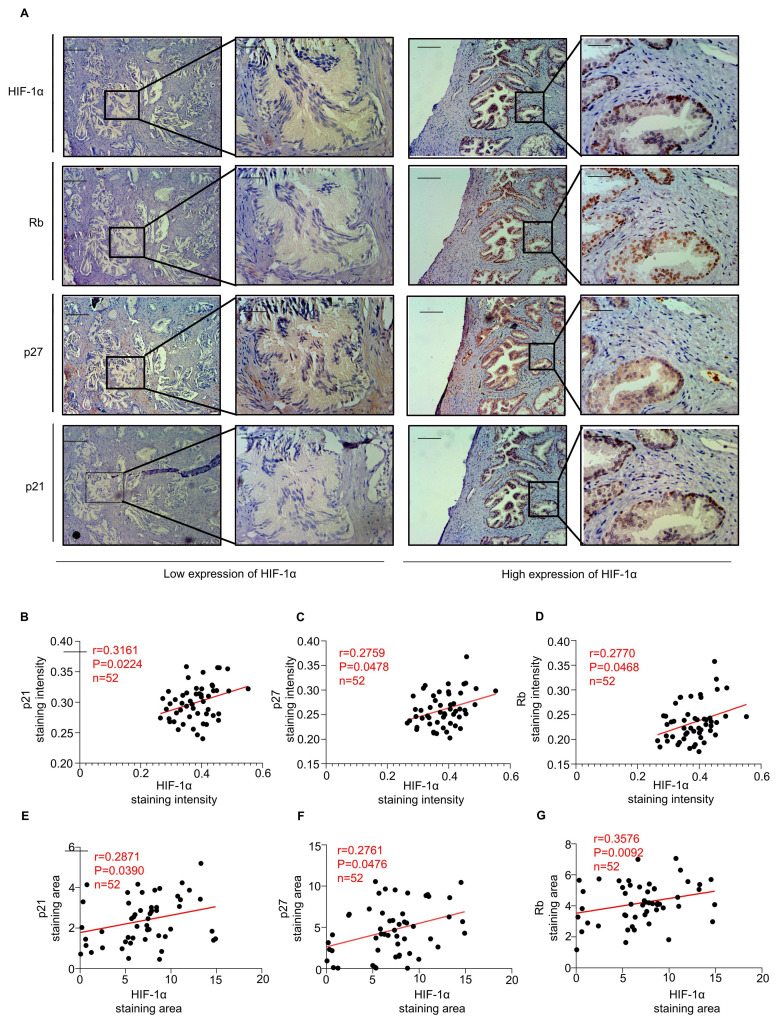
** Expression of HIF-1α and senescence markers in human BPH tissues.** (A) Representative immunohistochemical images from serial sections of the same specimen and corresponding tissue regions, illustrating the association between HIF-1α expression and p21, p27, and Rb staining. Areas with low HIF-1α expression are shown on the left, and areas with high HIF-1α expression are shown on the right. Scale bars = 400 μm for 100× images and 100 μm for 400× images. (B-D) Linear regression and correlation analyses showing the correlations between HIF-1α staining intensity and the staining intensities of p21, p27, and Rb. (E-G) Linear regression and correlation analyses showing the correlations between HIF-1α-positive staining area and the positive staining areas of p21, p27, and Rb.

Notably, immunohistochemical staining of serial paraffin sections from the same BPH specimen and corresponding tissue regions showed that epithelial areas with high HIF-1α expression exhibited stronger staining for p21, p27, and Rb (Fig. 2A-B). Linear regression and correlation analyses revealed that HIF-1α expression levels were positively correlated with staining intensities of p21 (r = 0.3161, P = 0.0224), p27 (r = 0.2759, P = 0.0478), and Rb (r = 0.2770, P = 0.0468) (Fig. 2C-D). Furthermore, the HIF-1α-positive staining area was also positively associated with the staining areas of p21 (r = 0.2871, P = 0.0390), p27 (r = 0.2761, P = 0.0476), and Rb (r = 0.3576, P = 0.0092) (Fig. 2E-G). These findings support a close association between HIF-1α expression and epithelial senescence-marker expression in human BPH tissues.

### Hypoxia/HIF-1α activation induces senescence-associated phenotypes in prostate epithelial cells

Based on the transcriptomic findings showing that epithelial cells, particularly luminal epithelial cells, exhibited prominent hypoxia/HIF-1α pathway activity and senescence-associated features in BPH tissues, we next performed *in vitro* experiments using the prostate epithelial cell lines BPH-1 and RWPE-1.

**Figure 3:**
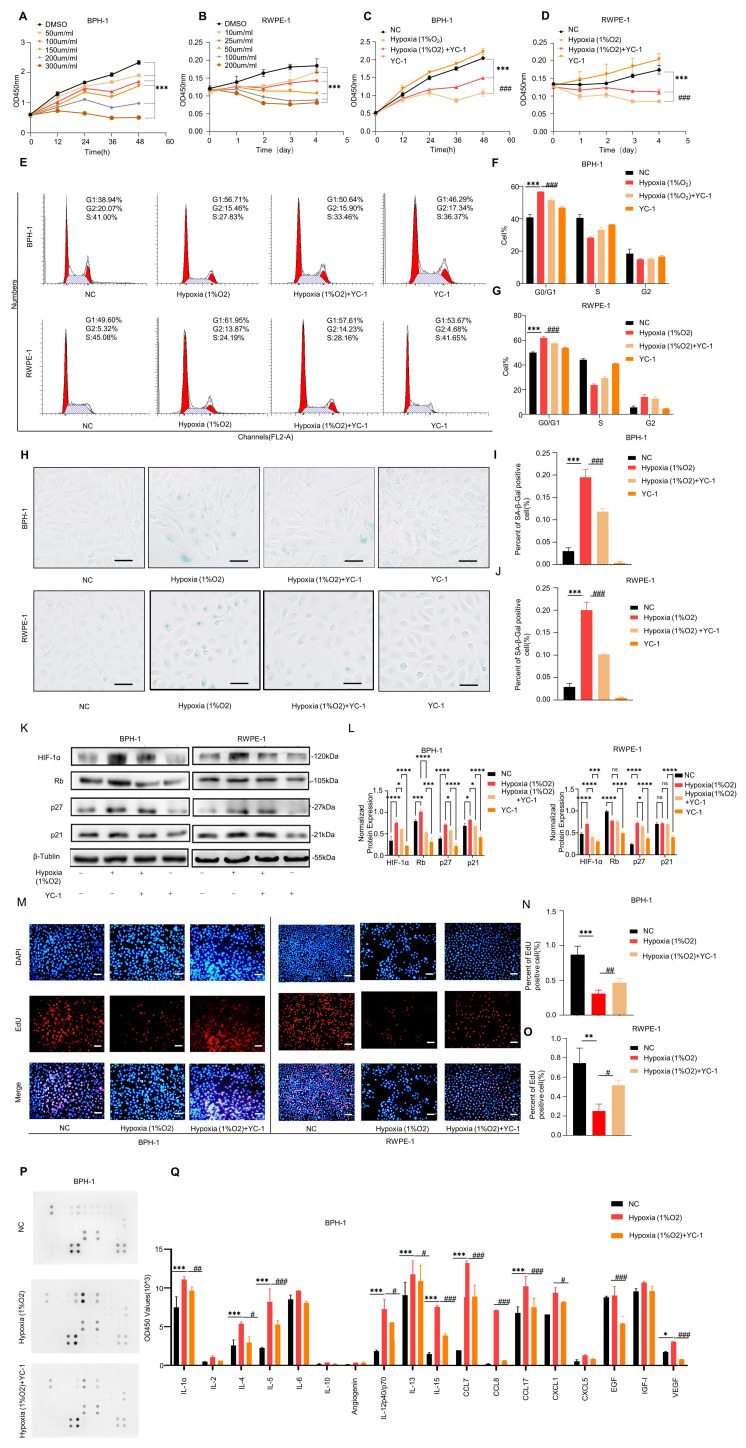
** Hypoxia activates HIF-1α and induces senescence in prostate epithelial cells.** (A-B) CCK-8 assays showing the proliferation of BPH-1 and RWPE-1 cells treated with increasing concentrations of CoCl₂ at the indicated time points. (C-D) CCK-8 assays showing the proliferation of BPH-1 and RWPE-1 cells after treatment with control, hypoxia (1% O₂), hypoxia (1% O₂) + YC-1 (10 μM), or YC-1 (10 μM) alone. (E-G) Flow cytometric analysis showing the cell-cycle distribution of BPH-1 and RWPE-1 cells after treatment with control, hypoxia (1% O₂), hypoxia (1% O₂) + YC-1, or YC-1 alone. Hypoxia increased the G0/G1-phase population, and this effect was attenuated by YC-1. (H-J) SA-β-gal staining of BPH-1 and RWPE-1 cells after treatment with control, hypoxia (1% O₂), hypoxia (1% O₂) + YC-1 (10 μM), or YC-1 alone. Scale bar = 100 μm. (K-L) Western blot analysis showing the expression of HIF-1α, p21, p27, and Rb in BPH-1 and RWPE-1 cells after treatment with control, hypoxia (1% O₂), hypoxia (1% O₂) + YC-1, or YC-1 alone. (M-O) EdU assays showing the proliferation of BPH-1 and RWPE-1 cells after treatment with control, hypoxia (1% O₂), or hypoxia (1% O₂) + YC-1. Scale bar = 100 μm. (P-Q) Cytokine antibody array analysis of SASP-associated secreted factors in BPH-1 cell culture supernatants after treatment with control, hypoxia (1% O₂), or hypoxia (1% O₂) + YC-1. All data are presented as the mean ± standard deviation, n = 3. *P < 0.05, **P < 0.01, ***P < 0.001; #P < 0.05, ##P < 0.01, ###P < 0.001.

Hypoxia mimetics, such as cobalt chloride (CoCl₂), can activate the HIF-1α signaling pathway by inhibiting prolyl hydroxylase activity 28. As an initial dose-response assessment, BPH-1 and RWPE-1 cells were treated with increasing concentrations of CoCl₂, and CCK-8 assays showed a dose-dependent decrease in cell viability in both cell lines (Fig. 3A-B).

We then examined the effects of hypoxia and HIF-1α inhibition under four conditions: control, hypoxia (1% O₂), hypoxia (1% O₂) + YC-1, and YC-1 alone. CCK-8 assays revealed that hypoxia significantly inhibited cell proliferation in both BPH-1 and RWPE-1 cells, whereas co-treatment with the HIF-1α inhibitor YC-1 partially reversed this effect (Fig. 3C-D). Flow cytometric analysis showed an increased G0/G1-phase population under hypoxic conditions, consistent with cell-cycle arrest, and this effect was alleviated by YC-1 co-treatment (Fig. 3E-G). SA-β-gal staining further showed that hypoxia promoted cellular senescence in both BPH-1 and RWPE-1 cells, whereas YC-1 attenuated this effect (Fig. 3H-J).

Western blot analysis showed that hypoxia increased HIF-1α protein levels and upregulated classical senescence markers, including p21, p27, and Rb. These effects were partially reversed by YC-1 (Fig. 3K-L). EdU incorporation assays demonstrated decreased DNA synthesis under hypoxia, which was also alleviated by YC-1 co-treatment (Fig. 3M-O). Cytokine antibody array analysis of BPH-1 cell culture supernatants showed that hypoxia increased the abundance of several SASP-associated cytokines, including IL-1α, IL-4, IL-13, and IL-15, and these increases were attenuated by YC-1 treatment (Fig. 3P-Q).

The efficiency of HIF-1α overexpression and si*HIF1A*-mediated reversal was confirmed by qRT-PCR and western blotting. Specifically, *HIF1A* mRNA levels were markedly elevated in the HIF-1α overexpression group relative to those in the negative control group and were reduced after si*HIF1A* co-transfection (Fig. S10C). Consistently, western blot analysis and quantitative densitometry showed a corresponding increase in HIF-1α protein in the HIF-1α overexpression group and a marked decrease after si*HIF1A* co-transfection (Fig. S10F, H). Functionally, HIF-1α overexpression increased the G0/G1-phase fraction, reduced EdU incorporation, and enhanced SA-β-gal staining in prostate epithelial cells, all of which were partially reversed by si*HIF1A* co-transfection (Fig. S11A-I).

Collectively, these results indicate that hypoxia/HIF-1α activation promotes senescence-associated phenotypes in prostate epithelial cell models.

### Hypoxia-treated epithelial cells with senescence-associated phenotypes promote stromal cell proliferation through a HIF-1α-dependent SASP-like secretory program

To investigate whether hypoxia-treated prostate epithelial cells with senescence-associated phenotypes promote stromal cell proliferation through paracrine effects, conditioned media (CM) were collected from BPH-1 and RWPE-1 cells under three conditions: normoxia (NC), hypoxia, and hypoxia combined with *HIF1A* knockdown.

**Figure 4:**
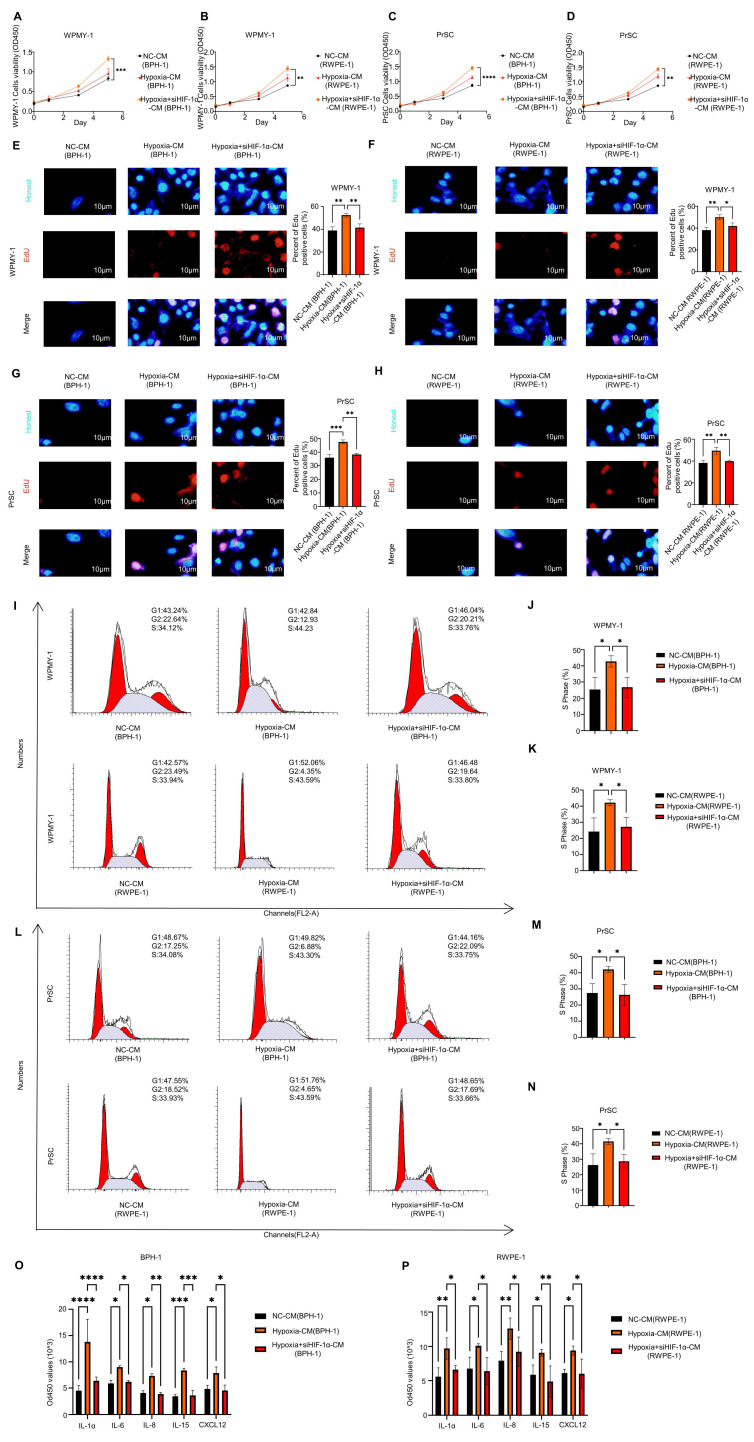
** Hypoxia-treated epithelial cells with senescence-associated phenotypes promote stromal cell proliferation through a HIF-1α-dependent SASP-like secretory program.** (A-D) CCK-8 assays showing the proliferative responses of stromal cells after stimulation with conditioned media (CM) derived from prostate epithelial cells under different treatment conditions. (A) WPMY-1 cells treated with CM from BPH-1 cells in the NC, Hypoxia, and Hypoxia + si*HIF1A* groups. (B) WPMY-1 cells treated with CM from RWPE-1 cells in the NC, Hypoxia, and Hypoxia + si*HIF1A* groups. (C) PrSCs treated with CM from RWPE-1 cells in the NC, Hypoxia, and Hypoxia + si*HIF1A* groups. (D) PrSCs treated with CM from BPH-1 cells in the NC, Hypoxia, and Hypoxia + si*HIF1A* groups. Hypoxia-CM significantly promoted stromal cell proliferation, whereas *HIF1A* knockdown in epithelial cells attenuated this effect. (E-H) EdU assays showing DNA synthesis in stromal cells after CM stimulation. (E) WPMY-1 cells treated with BPH-1-derived CM. (F) WPMY-1 cells treated with RWPE-1-derived CM. (G) PrSCs treated with BPH-1-derived CM. (H) PrSCs treated with RWPE-1-derived CM. Scale bar = 10 μm. Hypoxia-CM increased the proportion of EdU-positive cells, and this effect was partially reversed by *HIF1A* knockdown. (I, L) Representative flow cytometric plots showing cell-cycle distribution of stromal cells after CM stimulation. (I) WPMY-1 cells treated with BPH-1- or RWPE-1-derived CM. (L) PrSCs treated with BPH-1- or RWPE-1-derived CM. (J, K, M, N) Quantification of the S-phase fraction in stromal cells after CM stimulation. (J) WPMY-1 cells treated with BPH-1-derived CM. (K) WPMY-1 cells treated with RWPE-1-derived CM. (M) PrSCs treated with BPH-1-derived CM. (N) PrSCs treated with RWPE-1-derived CM. Hypoxia-CM increased the proportion of stromal cells in S phase, whereas Hypoxia + si*HIF1A*-CM partially reversed this effect. (O-P) Detection of SASP-associated factors in epithelial CM. (O) Levels of IL-1α, IL-6, IL-8, IL-15, and CXCL12 in BPH-1-derived CM. (P) Levels of IL-1α, IL-6, IL-8, IL-15, and CXCL12 in RWPE-1-derived CM. Hypoxia increased the secretion of multiple SASP-associated cytokines and chemokines, and these increases were reduced after *HIF1A* knockdown. All data are presented as the mean ± standard deviation, n = 3. *P < 0.05, **P < 0.01, ***P < 0.001.

CCK-8 assays showed that CM derived from hypoxia-treated epithelial cells significantly enhanced stromal cell proliferation compared with NC-CM, whereas *HIF1A* knockdown attenuated this effect (Fig. 4A-D). In WPMY-1 cells, BPH-1-derived Hypoxia-CM increased the OD450 value at day 5 to 1.33 ± 0.06, compared with 0.83 ± 0.06 in the NC-CM group, whereas Hypoxia + si*HIF1A*-CM reduced this value to 0.96 ± 0.12 (Fig. 4A). Similarly, RWPE-1-derived Hypoxia-CM increased the day-5 OD450 value in WPMY-1 cells to 1.44 ± 0.09, compared with 0.87 ± 0.02 in the NC-CM group, and this increase was partially reversed in the Hypoxia + si*HIF1A*-CM group (1.13 ± 0.11) (Fig. 4B). In PrSCs, RWPE-1-derived Hypoxia-CM increased the OD450 value to 1.45 ± 0.06, compared with 0.87 ± 0.05 in the NC-CM group, whereas Hypoxia + si*HIF1A*-CM reduced it to 1.14 ± 0.05 (Fig. 4C). Consistently, BPH-1-derived Hypoxia-CM increased PrSC proliferation to 1.42 ± 0.07, compared with 0.87 ± 0.03 in the NC-CM group, whereas Hypoxia + si*HIF1A*-CM reduced the value to 1.18 ± 0.08 (Fig. 4D). These findings indicate that hypoxia-treated epithelial cells acquire a stromal growth-promoting capacity that is at least partly dependent on *HIF1A*/HIF-1α signaling.

EdU incorporation assays further confirmed the pro-proliferative effect of hypoxic epithelial CM on stromal cells (Fig. 4E-H). In WPMY-1 cells, the percentage of EdU-positive cells increased from 38.90 ± 3.22% in the NC-CM group to 52.40 ± 1.70% after stimulation with BPH-1-derived Hypoxia-CM and decreased to 41.40 ± 3.35% in the Hypoxia + si*HIF1A*-CM group (Fig. 4E). Similar results were observed with RWPE-1-derived CM, in which the EdU-positive fraction increased from 38.07 ± 2.68% to 49.90 ± 2.31% and then decreased to 41.93 ± 2.79% after *HIF1A* knockdown (Fig. 4F). In PrSCs, BPH-1-derived Hypoxia-CM increased the EdU-positive fraction from 35.90 ± 2.45% to 47.53 ± 1.42%, whereas Hypoxia + si*HIF1A*-CM reduced it to 38.27 ± 0.78% (Fig. 4G). A similar pattern was observed with RWPE-1-derived CM, with corresponding values of 38.27 ± 2.46%, 49.53 ± 3.14%, and 39.93 ± 0.96% in the NC-CM, Hypoxia-CM, and Hypoxia + si*HIF1A*-CM groups, respectively (Fig. 4H). These data indicate that soluble factors released by hypoxic epithelial cells enhance stromal DNA synthesis and that this effect is weakened by *HIF1A* silencing.

Flow cytometric analysis of the cell cycle further demonstrated that hypoxic epithelial CM promoted stromal cell-cycle progression (Fig. 4I-N). Representative cell-cycle plots are shown in Fig. 4I and Fig. 4L. In WPMY-1 cells stimulated with BPH-1-derived CM, the S-phase fraction increased from 25.37 ± 7.58% in the NC-CM group to 42.78 ± 3.49% in the Hypoxia-CM group and decreased to 26.75 ± 6.16% in the Hypoxia + si*HIF1A*-CM group (Fig. 4J). Similar changes were observed in WPMY-1 cells treated with RWPE-1-derived CM, with S-phase fractions of 24.28 ± 8.43%, 42.23 ± 1.79%, and 27.13 ± 5.94%, respectively (Fig. 4K). In PrSCs, BPH-1-derived Hypoxia-CM increased the S-phase fraction from 27.49 ± 5.72% to 42.13 ± 1.85%, whereas Hypoxia + si*HIF1A*-CM reduced it to 26.32 ± 6.48% (Fig. 4M). RWPE-1-derived Hypoxia-CM showed a similar effect, increasing the S-phase fraction from 26.24 ± 7.26% to 41.53 ± 1.88%, which was partially reversed to 28.72 ± 4.34% after *HIF1A* knockdown (Fig. 4N). Together, these results suggest that hypoxic epithelial CM promotes stromal cell proliferation by facilitating cell-cycle entry and S-phase progression.

To determine whether this paracrine effect was associated with a SASP-like secretory phenotype, we measured representative cytokines in epithelial CM (Fig. 4O-P). In BPH-1-derived CM, hypoxia markedly increased the secretion of IL-1α from 4.53 ± 0.95 to 13.80 ± 4.30, IL-6 from 5.87 ± 0.60 to 9.00 ± 0.26, IL-8 from 4.10 ± 0.46 to 7.33 ± 0.40, IL-15 from 3.47 ± 0.35 to 8.37 ± 0.42, and CXCL12 from 4.83 ± 0.65 to 7.87 ± 1.16 (Fig. 4O). These elevations were generally reduced in the Hypoxia + si*HIF1A*-CM group, with corresponding values of 6.37 ± 0.74, 6.17 ± 0.25, 3.90 ± 0.30, 3.63 ± 0.97, and 4.57 ± 1.01, respectively. In RWPE-1-derived CM, hypoxia similarly increased the secretion of IL-1α from 5.60 ± 1.28 to 9.70 ± 1.55, IL-6 from 6.77 ± 1.68 to 10.10 ± 0.30, IL-8 from 7.93 ± 1.33 to 12.60 ± 1.54, IL-15 from 5.87 ± 1.42 to 9.07 ± 0.47, and CXCL12 from 6.13 ± 0.55 to 9.40 ± 0.66 (Fig. 4P), and these increases also showed a decreasing trend after *HIF1A* knockdown.

Notably, several cytokines measured in the present study, particularly IL-1α, IL-8, and CXCL12, have previously been implicated in proliferative epithelial-stromal crosstalk in BPH or the aging prostate microenvironment, whereas IL-6 and IL-15 are recognized inflammatory/SASP-associated mediators in the hyperplastic prostate 30. Therefore, these results support the notion that hypoxia induces a HIF-1α-dependent SASP-like secretory program in prostate epithelial cells, which in turn promotes stromal cell proliferation through a biologically relevant paracrine network rather than through nonspecific conditioned-medium effects.

Overall, these data indicate that hypoxia-treated epithelial cells with senescence-associated phenotypes promote stromal cell proliferation through a partly HIF-1α-dependent paracrine mechanism accompanied by increased secretion of SASP-associated factors.

### NF-κB signaling contributes to HIF-1α-mediated epithelial senescence in BPH

Because transcriptomic analyses indicated that NF-κB signaling was enriched in senescent epithelial cells and in epithelial cells with high hypoxia/HIF-1α pathway activity (Fig. 1E-F), we next examined whether NF-κB signaling contributes functionally to HIF-1α-associated epithelial senescence.

To further clarify the temporal relationship between HIF-1α accumulation and canonical NF-κB pathway activation, we performed a time-course western blot analysis in BPH-1 cells exposed to 1% O₂ hypoxia. HIF-1α protein levels remained largely unchanged at the earliest time points but increased from group 3 onward. In contrast, phosphorylation of IKKα, IKKβ, and IκBα remained low during groups 1-3 and increased mainly at groups 4-5. Total IKKα and IKKβ levels remained relatively stable, whereas total IκBα decreased at later time points, consistent with phosphorylation-associated IκBα degradation. These temporal patterns suggest that hypoxia-induced HIF-1α accumulation precedes detectable activation of the canonical IKK/IκBα signaling module. Together with our previous results showing p65 phosphorylation, IκB degradation, and senescence-marker induction, these findings support HIF-1α/NF-κB-linked pathway activation during hypoxia-associated epithelial senescence, while not excluding reciprocal regulation between HIF-1α and NF-κB signaling (Fig. S10A-B).

**Figure 5:**
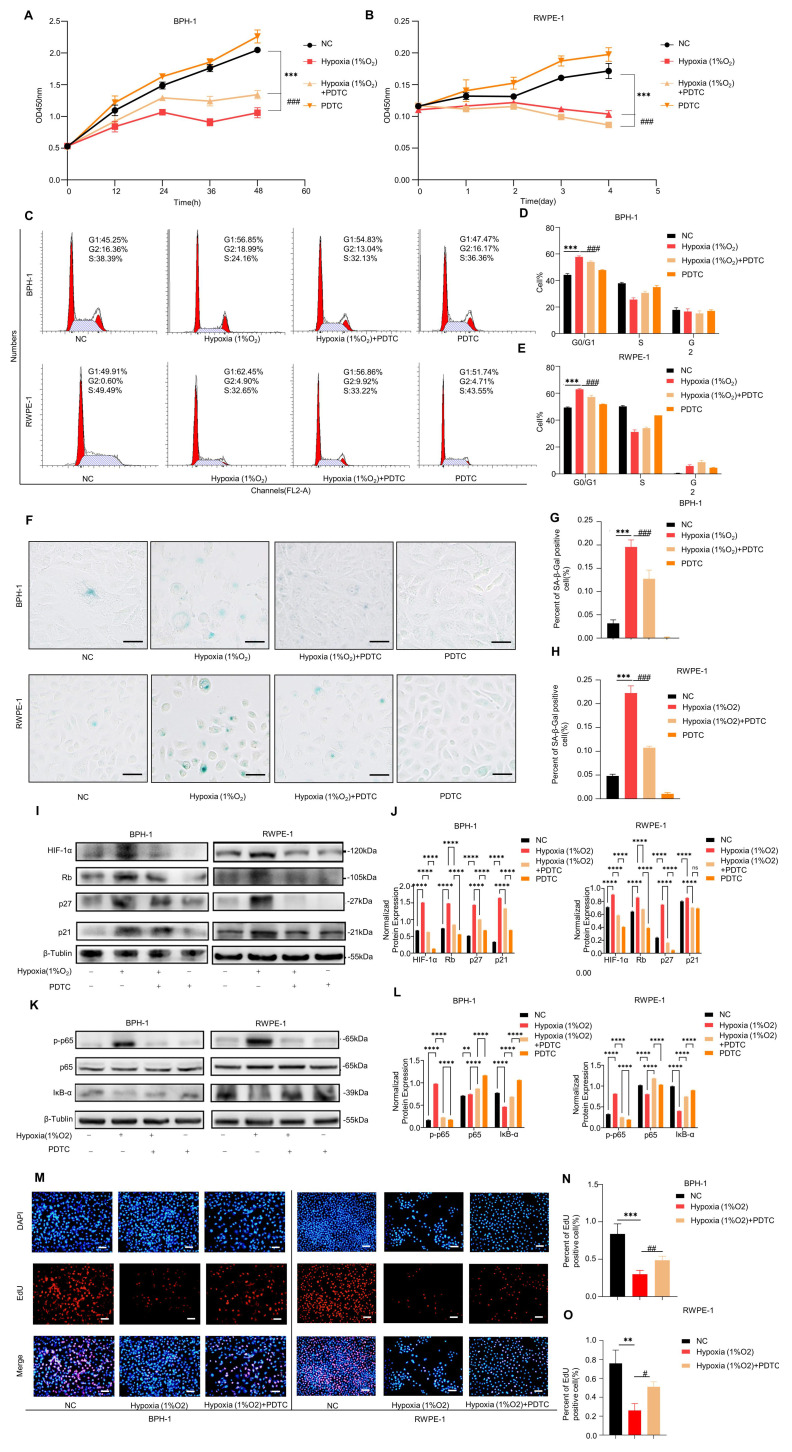
** NF-κB signaling contributes to hypoxia-induced senescence in prostate epithelial cells.** (A-B) CCK-8 assays showing the proliferation of BPH-1 and RWPE-1 cells after treatment with control, hypoxia (1% O₂), hypoxia (1% O₂) + PDTC, or PDTC alone. (C-E) Flow cytometric analysis showing the cell-cycle distribution of BPH-1 and RWPE-1 cells after treatment with control, hypoxia (1% O₂), hypoxia (1% O₂) + PDTC, or PDTC alone. (F-H) SA-β-gal staining of BPH-1 and RWPE-1 cells after treatment with control, hypoxia (1% O₂), hypoxia (1% O₂) + PDTC, or PDTC alone. Scale bar = 100 μm. (I-J) Western blot analysis showing the expression of HIF-1α, Rb, p27, and p21 in BPH-1 and RWPE-1 cells after treatment with control, hypoxia (1% O₂), hypoxia (1% O₂) + PDTC, or PDTC alone. (K-L) Western blot analysis showing the protein levels of phosphorylated p65 (p-p65) and IκB-α in BPH-1 and RWPE-1 cells after treatment with control, hypoxia (1% O₂), hypoxia (1% O₂) + PDTC, or PDTC alone. (M-O) EdU assays showing the proliferation of BPH-1 and RWPE-1 cells after treatment with control, hypoxia (1% O₂), or hypoxia (1% O₂) + PDTC. Scale bar = 100 μm.All data are presented as the mean ± standard deviation, n = 3. *P < 0.05, **P < 0.01, ***P < 0.001; #P < 0.05, ##P < 0.01.

CCK-8 assays showed that hypoxia (1% O₂) significantly inhibited cell proliferation, whereas co-treatment with the NF-κB inhibitor PDTC partially restored cell proliferation in both BPH-1 (Fig. 5A) and RWPE-1 cells (Fig. 5B). Cell-cycle analysis revealed a marked increase in the proportion of cells in G1 phase under hypoxia, which was attenuated by PDTC treatment (Fig. 5C-E). Similarly, SA-β-gal staining demonstrated that PDTC alleviated hypoxia-induced senescence in both cell lines (Fig. 5F-H).

Western blot analysis further showed that hypoxia induced p65 phosphorylation, IκB degradation, and upregulation of senescence markers, including p21, p27, and Rb, all of which were mitigated by PDTC co-treatment (Fig. 5I-L). EdU assays confirmed that hypoxia suppressed cell proliferation, whereas PDTC partially reversed this suppression in both cell lines (Fig. 5M-O).

The efficiency of the genetic manipulations was confirmed by qRT-PCR and western blotting. *HIF1A* mRNA levels remained elevated in the HIF-1α overexpression + si*RELA* group relative to those in the negative control group, indicating sustained HIF-1α overexpression (Fig. S10D), whereas *RELA* mRNA levels were markedly reduced after si*RELA* transfection (Fig. S10E). Consistently, western blot analysis and quantitative densitometry showed that HIF-1α protein remained elevated, whereas p65 protein was effectively reduced in the HIF-1α overexpression + si*RELA* group (Fig. S10G-J). To further examine the role of NF-κB signaling in HIF-1α-mediated senescence, we knocked down *RELA*, which encodes p65, a key transcription factor in the canonical NF-κB pathway. HIF-1α overexpression increased G0/G1-phase arrest, reduced EdU incorporation, and enhanced SA-β-gal staining, whereas si*RELA* co-transfection partially reversed these effects in BPH-1 and RWPE-1 cells (Fig. S12A-I).

Together, these findings suggest that NF-κB activation is functionally involved in HIF-1α-associated epithelial senescence in BPH, while not excluding potential reciprocal regulation between HIF-1α and NF-κB signaling.

### Single-cell RNA sequencing reveals cellular heterogeneity and hypoxia-associated epithelial states in a castration plus testosterone-induced mouse model of BPH

Using the GSE212770 dataset, we constructed a single-cell transcriptomic atlas of the prostate in a castration plus testosterone-induced mouse model of BPH. A total of 7,316 cells were categorized into nine major lineages based on canonical marker-gene expression: T/NK cells (418 cells, 5.7%; markers: *Il2rb*, *Nkg7*), B cells (211 cells, 2.9%; *Cd19*, *Pax5*), myeloid cells (1,260 cells, 17.2%; *Mpeg1*, *Clec4a2*), epithelial cells (2,653 cells, 35.7%; *Krt18*, *Krt8*, *Krt5*, *Krt15*, *Msmb*, *Gsdma*), mast cells (44 cells, 0.6%; *Cma1*, *Tpsab1*), myofibroblasts (241 cells, 3.3%; *Acta2*, *Pdgfra*), fibroblasts (1,838 cells, 25.1%; *Col1a1*, *Mfap2*), smooth muscle cells (94 cells, 1.3%; *Rgs5*, *Actg2*), and endothelial cells (557 cells, 7.6%; *Cdh5*, *Sox18*) (Fig. S13A-B; Fig. S15A-B). Epithelial cells were the most abundant population, followed by stromal and immune compartments.

Gene-expression profiles of representative markers confirmed lineage identity: *Il2rb* and *Nkg7* for T/NK cells, *Cd19* and *Pax5* for B cells, *Mpeg1* and *Clec4a2* for myeloid cells, *Krt18* and *Krt8* for luminal epithelial cells, and *Krt5* and *Krt15* for basal cells (Fig. S15C). Stromal markers, such as *Col1a1*, and vascular markers, such as *Cdh5* and *Sox18*, confirmed fibroblast and endothelial identities, respectively. Importantly, the small epithelial cluster previously annotated as neuroendocrine cells did not show enrichment of canonical neuroendocrine markers, including *Syp*, *Chga*, *Insm1*, and *Ascl1* (Fig. S14A), while retaining epithelial-marker expression, including *Epcam*, *Krt8*, *Krt18*, and *Krt19* (Fig. S14B). In addition, this cluster did not fit the classical basal program and was distinct from the major luminal clusters (Fig. S14C). Therefore, to avoid overinterpretation, we conservatively reannotated this population as rare epithelial cells.

To evaluate hypoxia-associated pathway activity, GSVA was performed using the GROSS_HYPOXIA_VIA_HIF1A_UP gene set. Hypoxia pathway activation was prominent in epithelial and stromal cells (Fig. S15D). Epithelial cells were further stratified into HIF-1α pathway-high and HIF-1α pathway-low subgroups based on GSVA scores (Fig. S15E), revealing transcriptional heterogeneity within the epithelial compartment.

Differential expression analysis revealed upregulation of multiple genes related to hypoxia/HIF signaling, NF-κB-associated inflammatory responses, and senescence or cell-cycle regulation in the HIF-1α pathway-high epithelial subgroup, including *Aldoa*, *Pgk1*, *Ldha*, *Ddit4*, *Nfkbia*, *Cdkn1b*, *Jun*, *Junb*, and *Fos* (Fig. S15F). Pathway enrichment analysis demonstrated activation of hypoxia-related pathways, including ELVIDGE_HYPOXIA_UP (NES = 1.98, P = 0.0024), cell-cycle regulation, including GOBP_REGULATION_OF_CELL_CYCLE (NES = 2.01, P = 0.00043), and cellular aging, including GOBP_CELL_AGING (NES = 1.87, P = 0.014), in the HIF-1α pathway-high epithelial group (Fig. S15G).

Further enrichment analysis confirmed that REACTOME_CELLULAR_RESPONSE_TO_HYPOXIA, FRIDMAN_SENESCENCE_UP, and HALLMARK_TNFA_SIGNALING_VIA_NFKB were enriched in HIF-1α pathway-high epithelial cells (Fig. S15H). These findings suggest coordinated activation of hypoxia-, NF-κB-, and senescence-associated transcriptional programs in epithelial cells of the mouse BPH model.

Epithelial subtypes were identified as Luminal cells, Basal cells, and rare epithelial cells (Fig. S15I). Luminal cells comprised the majority of epithelial cells with above-mean GSVA scores for the GROSS_HYPOXIA_VIA_HIF1A_UP pathway (Fig. S15J) and were further stratified into HIF-1α pathway-high and HIF-1α pathway-low groups (Fig. S15K). In HIF-1α pathway-high Luminal cells, *Ddit4* and *Cdkn1b* were among the representative upregulated genes associated with the hypoxia-related transcriptional state (Fig. S15L). Pathway analysis revealed enrichment of hypoxia-responsive pathways, including ELVIDGE_HYPOXIA_UP (NES = 2.22, P = 0.0014), cell-cycle regulation, including GOBP_REGULATION_OF_CELL_CYCLE (NES = 2.02, P = 0.0057), and aging-related processes, including GOBP_AGING (NES = 2.22, P = 0.00086) (Fig. S15M).

Broadly enriched biological processes in HIF-1α pathway-high Luminal cells included hypoxia response, represented by REACTOME_CELLULAR_RESPONSE_TO_HYPOXIA; NF-κB signaling, represented by HALLMARK_TNFA_SIGNALING_VIA_NFKB; and senescence-associated programs, represented by FRIDMAN_SENESCENCE_UP (Fig. S15N). These findings further support the presence of coordinated hypoxia-, NF-κB-, and senescence-associated transcriptional states in epithelial cells in the mouse BPH model.

### Pharmacological inhibition of HIF-1α or NF-κB attenuates testosterone propionate-induced prostatic hyperplasia and senescence-associated changes *in vivo*

**Figure 6:**
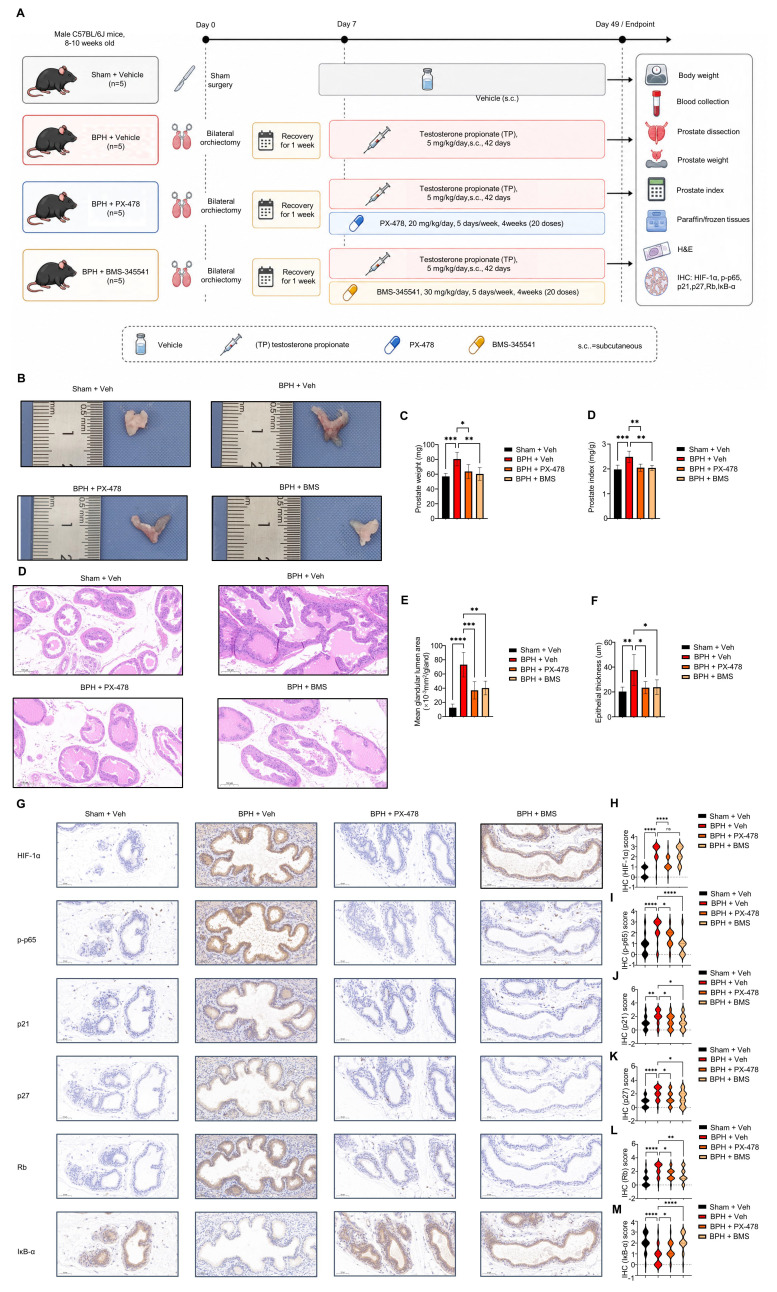
** Pharmacological inhibition of HIF-1α or NF-κB attenuates testosterone propionate-induced BPH-like prostatic hyperplasia and senescence-associated molecular changes *in vivo*.** (A) Schematic illustration of the animal experimental design. Male C57BL/6J mice were assigned to four groups: Sham + Vehicle, BPH + Vehicle, BPH + PX-478, and BPH + BMS-345541. Mice in the Sham + Vehicle group underwent sham surgery, whereas mice in the BPH groups underwent bilateral orchiectomy followed by testosterone propionate (TP) administration. PX-478 or BMS-345541 was administered during the TP induction period. (B) Representative gross images of dissected prostates from each group. (C) Quantification of prostate weight. (D) Quantification of prostate index. (E) Representative H&E staining of prostate tissues. (F) Quantification of mean glandular lumen area. (G) Quantification of epithelial thickness. (H) Representative serial IHC staining for HIF-1α, p-p65, p21, p27, Rb, and IκB-α in mouse prostate tissues. (I-N) Semiquantitative IHC scores of HIF-1α, p-p65, p21, p27, Rb, and IκB-α, respectively. Data are presented as mean ± SEM. n = 5 mice per group. For histological and IHC analyses, five randomly selected fields per mouse were evaluated. Field-level IHC scores are shown for visualization, whereas statistical analyses were performed using mouse-level mean values. Statistical significance was determined by one-way ANOVA followed by Tukey's multiple-comparison test. *P < 0.05, **P < 0.01, ***P < 0.001, ****P < 0.0001. Scale bars, 100 μm.

To strengthen the *in vivo* evidence for the involvement of HIF-1α/NF-κB-linked signaling in BPH-associated epithelial remodeling, we established a testosterone propionate (TP)-induced BPH-like prostatic hyperplasia model in orchiectomized male C57BL/6J mice. After a 1-week recovery period following orchiectomy, mice received TP administration for 42 days. BPH mice were further treated with the HIF-1α inhibitor PX-478 or the IKK/NF-κB pathway inhibitor BMS-345541 during the modeling period (Fig. 6A).

Gross examination of dissected prostates showed that the BPH + Vehicle group exhibited visible prostate enlargement compared with the Sham + Vehicle group, whereas this enlargement was attenuated in mice treated with PX-478 or BMS-345541 (Fig. 6B). Consistently, prostate weight was increased in the BPH + Vehicle group compared with the Sham + Vehicle group (80.5 ± 4.1 mg vs. 57.0 ± 1.9 mg). PX-478 and BMS-345541 treatment reduced prostate weight to 63.7 ± 4.2 mg and 60.4 ± 3.9 mg, respectively (Fig. 6C). Similarly, the prostate index was elevated in BPH + Vehicle mice compared with Sham + Vehicle mice (2.49 ± 0.10 mg/g vs. 1.98 ± 0.08 mg/g), whereas PX-478 and BMS-345541 treatment reduced the prostate index to 2.05 ± 0.06 mg/g and 2.04 ± 0.05 mg/g, respectively (Fig. 6D).

H&E staining further confirmed the BPH-like histological phenotype induced by TP. Compared with Sham + Vehicle mice, BPH + Vehicle mice displayed epithelial thickening, increased epithelial folding, irregular glandular architecture, and enlarged glandular lumens. These histological abnormalities were alleviated in the BPH + PX-478 and BPH + BMS-345541 groups (Fig. 6E). Quantitative morphometric analysis showed that the mean glandular lumen area was markedly increased in the BPH + Vehicle group compared with the Sham + Vehicle group (73.18 ± 7.76 vs. 12.66 ± 2.27 ×10^-3 mm²/gland). PX-478 and BMS-345541 reduced the mean glandular lumen area to 37.04 ± 5.50 and 40.32 ± 4.34 ×10^-3 mm²/gland, respectively (Fig. 6F). In parallel, epithelial thickness was increased in BPH + Vehicle mice compared with Sham + Vehicle mice (37.64 ± 5.57 μm vs. 20.32 ± 1.64 μm), whereas PX-478 and BMS-345541 reduced epithelial thickness to 23.48 ± 2.22 μm and 23.84 ± 2.65 μm, respectively (Fig. 6G).

We next examined whether these pathological changes were accompanied by alterations in HIF-1α/NF-κB signaling and senescence-associated markers *in vivo*. Serial prostate sections were stained for HIF-1α, p-p65, p21, p27, Rb, and IκB-α (Fig. 6H). HIF-1α staining was markedly increased in BPH + Vehicle mice compared with Sham + Vehicle mice, with the mean IHC score increasing from 0.48 to 2.48. PX-478 substantially reduced HIF-1α staining to 1.36, whereas BMS-345541 showed only a limited effect on HIF-1α staining, with a mean score of 2.20 (Fig. 6I). Conversely, p-p65 staining was increased in BPH + Vehicle mice compared with Sham + Vehicle mice, with the mean score increasing from 0.88 to 2.24. BMS-345541 markedly reduced p-p65 staining to 0.76, while PX-478 partially reduced it to 1.56 (Fig. 6J). These results are consistent with the expected *in vivo* pathway engagement of the two inhibitors, with PX-478 primarily suppressing HIF-1α-associated signaling and BMS-345541 primarily inhibiting canonical NF-κB activation.

We further assessed senescence- and cell cycle arrest-associated markers in mouse prostate tissues. Compared with Sham + Vehicle mice, BPH + Vehicle mice showed increased staining for p21, p27, and Rb. The mean IHC scores increased from 1.04 to 1.96 for p21, from 0.76 to 2.08 for p27, and from 0.76 to 2.24 for Rb. PX-478 reduced the scores of p21, p27, and Rb to 1.24, 1.36, and 1.60, respectively. BMS-345541 similarly reduced these scores to 1.20, 1.36, and 1.44, respectively (Fig. 6K-M). In addition, IκB-α staining was decreased in BPH + Vehicle mice compared with Sham + Vehicle mice, with the mean score decreasing from 2.08 to 0.76, consistent with activation of the canonical NF-κB pathway. This reduction was partially restored by PX-478 and more markedly restored by BMS-345541, with mean scores of 1.36 and 2.12, respectively (Fig. 6N).

Together, these data indicate that pharmacological inhibition of HIF-1α or NF-κB attenuates TP-induced BPH-like prostatic hyperplasia, reduces epithelial hyperplastic remodeling, and partially reverses senescence-associated molecular changes *in vivo*. These findings provide *in vivo* pharmacological evidence supporting the involvement of HIF-1α/NF-κB-linked signaling in BPH-associated epithelial proliferation and senescence-like changes.

## Discussion

In this study, we characterized the spatial distribution and molecular features of senescence-associated epithelial states in BPH tissues and found that senescence-associated features were preferentially enriched within the epithelial compartment, particularly in luminal epithelial cells and Luminal-dominant spatial spots. Multi-layered transcriptomic analyses further indicated that epithelial senescence in BPH is accompanied by hypoxia-associated HIF-1α/NF-κB pathway activation. Because BPH is strongly associated with aging, and because different tissue compartments exhibit distinct microenvironmental and senescence-related profiles, defining the localization and regulatory context of epithelial senescence is important for understanding BPH pathogenesis. Although emerging evidence has implicated cellular senescence in BPH development 31, the specific spatial localization and molecular regulation of senescent cells in human prostate tissue remain incompletely understood.

In our cohort of 52 BPH specimens, 34.62% of cases (18/52) showed positive SA-β-gal staining, with SA-β-gal-positive cells predominantly concentrated in the epithelial compartment and only minimal staining observed in stromal regions. These findings are consistent with previous reports indicating the presence of senescence-associated epithelial alterations in BPH tissues 12,13. Further analysis revealed that the distribution of SA-β-gal positivity was heterogeneous at the glandular level: 13.51% of glands showed diffuse positivity, 12.52% showed partial positivity, and 73.97% showed no detectable positivity. These results suggest that epithelial senescence in BPH is not uniformly distributed but instead occurs in a focal and heterogeneous manner.

This heterogeneity may reflect intrinsic differences in the biological roles and stress responses of epithelial and stromal cells 5,32. Prostatic epithelial cells, particularly luminal epithelial cells, have substantial secretory and metabolic activity required for glandular function and epithelial turnover. These features may render them more susceptible to senescence-associated changes under microenvironmental stressors such as hypoxia, inflammation, and hormonal perturbation. Stromal cells, by contrast, are largely structural and supportive components of the prostate microenvironment and may respond to these stressors through different biological programs.

Single-cell RNA sequencing further showed that luminal epithelial cells exhibited the strongest hypoxia/HIF-1α pathway activity among epithelial subtypes, while spatial transcriptomic analysis indicated that Luminal-dominant spots showed high senescence-associated and hypoxia-response scores. Specifically, HIF-1α pathway-high luminal epithelial subsets displayed coordinated enrichment of hypoxia-responsive, NF-κB-related, and senescence-associated transcriptional programs. These findings suggest that luminal epithelial cells are prominent responders to the hypoxic prostate microenvironment and may represent a major epithelial population in which hypoxia-associated senescence-related programs are enriched in BPH.

Hypoxia is a recognized feature of the BPH microenvironment. Previous color Doppler ultrasound studies have reported decreased perfusion and elevated vascular resistance in hyperplastic prostate regions, suggesting sustained tissue hypoxia. Hypoxia, together with oxidative stress, telomere attrition, and DNA damage, is a known contributor to cellular senescence 33,34. In the present study, immunohistochemical analyses showed that epithelial regions with higher HIF-1α expression also exhibited stronger staining for senescence-associated markers, including p21, p27, and Rb. Together with the LCM-based transcriptomic data, these findings support a close association between hypoxia/HIF-1α signaling and epithelial senescence-associated programs in BPH tissues.

HIF-1α is a master regulator of cellular adaptation to hypoxia and has been implicated in senescence regulation in multiple biological contexts. However, its role appears to be context-dependent 35,36. For example, HIF-1α has been reported to promote senescence in lung and breast cancer cells by modulating cell-cycle regulators 37, whereas in bone marrow stromal cells, it may delay senescence through the E2A-p21 axis 38. To clarify its role in BPH, we combined SA-β-gal-based LCM, transcriptomic profiling, public human and mouse single-cell datasets, spatial transcriptomics, immunohistochemistry, and perturbation-based *in vitro* experiments. These analyses consistently supported the association of HIF-1α pathway activation with senescence-associated epithelial states in BPH. *In vitro*, CoCl₂-mediated hypoxia-mimetic stimulation or 1% O₂ exposure induced G0/G1 arrest, SA-β-gal positivity, HIF-1α accumulation, p21/p27/Rb upregulation, and SASP-associated cytokine secretion in prostate epithelial cells. These changes were attenuated by pharmacological inhibition or knockdown of *HIF1A*, supporting the functional involvement of HIF-1α in hypoxia-associated epithelial senescence phenotypes.

We further identified NF-κB signaling as a functionally involved component of HIF-1α-associated epithelial senescence in BPH. Hypoxic exposure activated NF-κB signaling in prostate epithelial cells, as reflected by increased p65 phosphorylation and IκB degradation. Pharmacological inhibition of HIF-1α attenuated NF-κB activation, whereas knockdown of *RELA*, which encodes p65, partially reversed HIF-1α-induced senescence-associated phenotypes. These findings support a functional interaction between HIF-1α and NF-κB in hypoxia-treated prostate epithelial cells and suggest that NF-κB contributes to the senescence-associated phenotype observed in this context.

NF-κB is also a key regulator of the senescence-associated secretory phenotype (SASP), which can reshape the local tissue microenvironment 39,40. SASP factors, including IL-6, IL-8, and TNF-α, can exert autocrine effects that reinforce senescence and paracrine effects that influence neighboring non-senescent cells 41-43. In BPH, such paracrine signaling may contribute to epithelial-stromal crosstalk and promote stromal proliferative responses, thereby participating in glandular remodeling and disease progression 44,45. Consistent with this possibility, conditioned media from hypoxia-treated epithelial cells promoted stromal cell proliferation, enhanced EdU incorporation, and increased S-phase entry, whereas *HIF1A* knockdown attenuated these effects. In parallel, hypoxia increased the secretion of SASP-associated factors, including IL-1α, IL-6, IL-8, IL-15, and CXCL12, and these increases were generally reduced after *HIF1A* knockdown. These results suggest that hypoxia-treated epithelial cells with senescence-associated phenotypes exhibit a partly HIF-1α-dependent SASP-like secretory program that may contribute to stromal proliferative responses.

Previous studies have also shown that SASP factors can enhance immune surveillance by activating natural killer (NK) cells to selectively eliminate senescent cells 46. This suggests that the prostate microenvironment may involve a dynamic balance between senescence induction, paracrine signaling, and immune-mediated clearance of senescent cells. Although this aspect was not directly tested in the present study, it provides an important direction for future investigation. Overall, our findings support a working model in which focal epithelial senescence is accompanied by hypoxia-associated HIF-1α/NF-κB activation, SASP-like paracrine activity, and stromal proliferative responses in BPH.

Recent single-cell and spatial transcriptomic studies have also highlighted hypoxia-associated epithelial alterations in BPH. Notably, a recent study identified a BE5 basal epithelial subgroup characterized by increased hypoxia and FOS activity and linked this state to epithelial-mesenchymal transition-related remodeling during nodular formation. Our findings are complementary rather than redundant. Whereas that study emphasized basal epithelial remodeling and hypoxia-associated epithelial-mesenchymal transition features, our study focuses on luminal epithelial senescence and its association with coordinated HIF-1α/NF-κB activation, together with a SASP-like epithelial-stromal paracrine program. Therefore, our work refines the cell-type-specific and functional context of hypoxia-related pathology in BPH rather than proposing an entirely separate signaling paradigm.

### Limitations of the study

Several limitations should be acknowledged. First, the single-cell and spatial transcriptomic analyses primarily define cell states, spatial context, and coordinated pathway activation, but they do not independently establish strict directional causality in situ. In particular, the spatial transcriptomic analysis was based on 10x Genomics Visium data, which provide spot-level rather than true single-cell resolution. Because each spot may contain transcripts from multiple neighboring cells, the transferred labels used in this study should be interpreted as dominant spot identities or dominant cell states rather than pure single-cell annotations. Moreover, the UCell score based on GOBP_POSITIVE_REGULATION_OF_CELLULAR_SENESCENCE reflects relative activity of a senescence-associated regulatory program and does not by itself prove that each high-scoring spot contains fully senescent cells. Although we restricted the comparison between senescence-score-high and senescence-score-low groups to Luminal-dominant spots to reduce confounding from major cell-type mixing, this strategy cannot fully resolve intra-spot cellular heterogeneity. Future studies using higher-resolution spatial transcriptomics, spatial single-cell approaches, or multiplexed protein/RNA imaging will be needed to validate the precise cellular sources of these senescence-associated transcriptional programs.

Second, the LCM-based RNA-seq analysis was performed using SA-β-gal-positive and SA-β-gal-negative epithelial cells isolated from only three BPH patients. Therefore, these data should be interpreted as discovery-level transcriptomic evidence rather than definitive population-level validation. To mitigate this limitation, we integrated these findings with public human single-cell and spatial transcriptomic datasets, IHC analysis of 52 human BPH specimens, and perturbation-based *in vitro* and *in vivo* experiments.

Third, most perturbation experiments were performed in simplified *in vitro* models, including BPH-1 and RWPE-1 cells exposed to CoCl₂ or short-term 1% O₂. These conditions may not fully recapitulate the chronic, tissue-specific hypoxic microenvironment of human BPH. In addition, although genetic perturbation experiments and conditioned-medium assays provided functional support for the proposed epithelial-stromal paracrine mechanism, these systems still do not fully reproduce the long-term epithelial-stromal interactions that occur *in vivo*.

Fourth, the castration plus testosterone propionate-induced mouse model used in this study represents a hormone-driven model of BPH-like prostatic hyperplasia and does not fully recapitulate the chronic, age-associated microenvironment of human BPH. Therefore, the mouse data should be interpreted as supportive *in vivo* pharmacological evidence rather than definitive proof that HIF-1α/NF-κB signaling drives human BPH pathogenesis. Although PX-478 and BMS-345541 attenuated BPH-like histological changes and reduced senescence-associated marker expression *in vivo*, these pharmacological approaches do not provide cell-type-specific genetic evidence. Future studies using prostate epithelial-specific conditional knockout models, such as epithelial-specific Hif1a or Rela/Ikbkb deletion, will be required to establish cell-autonomous causality.

Finally, although our conditioned-medium and ELISA data support a SASP-like paracrine effect on stromal proliferation, the relative contribution of individual secreted mediators and the potential role of immune-mediated senescent-cell clearance remain to be clarified. Future studies incorporating lineage tracing, *in vivo* genetic perturbation, temporal analyses, primary epithelial cells, organoid models, long-term hypoxia exposure, and higher-resolution spatial validation will be required to further define the specificity, causality, and potential therapeutic relevance of the hypoxia-senescence relationship in BPH.

## Conclusion

In summary, this study shows that senescence-associated features in BPH are preferentially enriched in the epithelial compartment, particularly in luminal epithelial cells and Luminal-dominant spatial spots, and are accompanied by activation of hypoxia-associated HIF-1α/NF-κB signaling. Our data support a model in which hypoxia-associated HIF-1α activation is functionally linked to epithelial senescence-associated phenotypes, at least in part through NF-κB signaling, and induces SASP-like secretory changes that may contribute to epithelial-stromal crosstalk and stromal proliferative responses. These findings nominate the HIF-1α/NF-κB-associated hypoxia-senescence program as a candidate pathway for future mechanistic investigation and potential therapeutic exploration in BPH.

## Supplementary Material

Supplementary figures.

Supplementary table 1: Statistical results for the selected genes shown in Fig. 1G.

## Data Availability

The LCM RNA-seq dataset GSE297404 was previously reported in our recently published study on CXCL13-mediated CD4+ T-cell immune surveillance in BPH [48] and was reanalyzed here to investigate hypoxia/HIF-1α/NF-κB-linked epithelial senescence. Public scRNA-seq datasets were retrieved from GEO under accession numbers GSE226237, GSE183676, and GSE212770. Human spatial transcriptomic data were obtained from GEO under accession number GSE278936. Bulk RNA-seq data from mouse prostate tissues used for transcriptomic comparisons have been deposited in GEO under accession number GSE297417. Transcriptomic aging signatures were analyzed using the Aging-related Disease Expression and Immune Profiling platform, and senescence-associated gene lists were obtained from the CellAge database. All other data supporting the findings of this study are available within the article and its supplementary information files or from the corresponding authors upon reasonable request.

## Abbreviations

CCK-8: Cell Counting Kit-8
CM: conditioned medium
EGLN3: egl-9 family hypoxia-inducible factor 3
ELISA: enzyme-linked immunosorbent assay
GO: Gene Ontology
GSEA: gene set enrichment analysis
GSVA: Gene Set Variation Analysis
HIF-1α: hypoxia-inducible factor-1α
IHC: immunohistochemistry
IPSS: International Prostate Symptom Score
IκB-α: inhibitor of NF-κB
KEGG: Kyoto Encyclopedia of Genes and Genomes
LCM: laser capture microdissection
NF-κB: nuclear factor kappa-B
ORA: over-representation analysis
PDTC: pyrrolidinedithiocarbamate ammonium
PrSCs: primary human prostate stromal cells
RNA-seq: RNA sequencing
SASP: senescence-associated secretory phenotype
SA-β-gal: senescence-associated β-galactosidase
scRNA-seq: single-cell RNA sequencing
TP: testosterone propionate
TURP: transurethral resection of the prostate
UMAP: Uniform Manifold Approximation and Projection
YC-1: lificiguat

## References

[1] Chughtai B, Forde JC, Thomas DD, Laor L, Hossack T, Woo HH, Te AE, Kaplan SA (2016). Benign prostatic hyperplasia. Nat Rev Dis Primers.

[2] Zeng XT, Jin YH, Liu TZ, Chen FM, Ding DG, Fu M, Gu XQ, Han BM, Huang X, Hou Z (2022). Clinical practice guideline for transurethral plasmakinetic resection of prostate for benign prostatic hyperplasia (2021 Edition). Mil Med Res.

[3] Berry SJ, Coffey DS, Walsh PC, Ewing LL (1984). The development of human benign prostatic hyperplasia with age. J Urol.

[4] Castro P, Giri D, Lamb D, Ittmann M (2003). Cellular senescence in the pathogenesis of benign prostatic hyperplasia. Prostate.

[5] Vital P, Castro P, Tsang S, Ittmann M (2014). The senescence-associated secretory phenotype promotes benign prostatic hyperplasia. Am J Pathol.

[6] Jiang S, Song CS, Chatterjee B (2019). Stimulation of prostate cells by the senescence phenotype of epithelial and stromal cells: implication for benign prostate hyperplasia. FASEB Bioadv.

[7] Robert G, Descazeaud A, Nicolaiew N, Terry S, Sirab N, Vacherot F, Maille P, Allory Y, de la Taille A (2009). Inflammation in benign prostatic hyperplasia: a 282 patients' immunohistochemical analysis. Prostate.

[8] Nicholson TM, Ricke WA (2011). Androgens and estrogens in benign prostatic hyperplasia: past, present and future. Differentiation.

[9] Hayflick L, Moorhead PS (1961). The serial cultivation of human diploid cell strains. Exp Cell Res.

[10] López-Otín C, Blasco MA, Partridge L, Serrano M, Kroemer G (2023). Hallmarks of aging: an expanding universe. Cell.

[11] Campisi J (1996). Replicative senescence: an old lives' tale?. Cell.

[12] Castro P, Xia C, Gomez L, Lamb DJ, Ittmann M (2004). Interleukin-8 expression is increased in senescent prostatic epithelial cells and promotes the development of benign prostatic hyperplasia. Prostate.

[13] Choi J, Shendrik I, Peacocke M, Peehl D, Buttyan R, Ikeguchi EF, Katz AE, Benson MC (2000). Expression of senescence-associated beta-galactosidase in enlarged prostates from men with benign prostatic hyperplasia. Urology.

[14] Carnell DM, Smith RE, Daley FM, Saunders MI, Bentzen SM, Hoskin PJ (2006). An immunohistochemical assessment of hypoxia in prostate carcinoma using pimonidazole: implications for radioresistance. Int J Radiat Oncol Biol Phys.

[15] Berger AP, Horninger W, Bektic J, Pelzer A, Spranger R, Bartsch G, Frauscher F (2006). Vascular resistance in the prostate evaluated by colour Doppler ultrasonography: is benign prostatic hyperplasia a vascular disease?. BJU Int.

[16] Balaban RS, Nemoto S, Finkel T (2005). Mitochondria, oxidants, and aging. Cell.

[17] Gorgoulis V, Adams PD, Alimonti A, Bennett DC, Bischof O, Bishop C, Campisi J, Collado M, Evangelou K, Ferbeyre G (2019). Cellular senescence: defining a path forward. Cell.

[18] You L, Nepovimova E, Valko M, Wu Q, Kuca K (2022). Mycotoxins and cellular senescence: the impact of oxidative stress, hypoxia, and immunosuppression. Arch Toxicol.

[19] Du Z, Fujiyama C, Chen Y, Masaki Z (2003). Expression of hypoxia-inducible factor 1alpha in human normal, benign, and malignant prostate tissue. Chin Med J (Engl).

[20] Lekas A, Lazaris AC, Deliveliotis C, Chrisofos M, Zoubouli C, Lapas D, Papathomas T, Fokitis I, Nakopoulou L (2006). The expression of hypoxia-inducible factor-1alpha (HIF-1alpha) and angiogenesis markers in hyperplastic and malignant prostate tissue. Anticancer Res.

[21] Wu F, Ding S, Li X, Wang H, Liu S, Wu H, Bi D, Ding K, Lu J (2016). Elevated expression of HIF-1α in actively growing prostate tissues is associated with clinical features of benign prostatic hyperplasia. Oncotarget.

[22] Chen Y, Xu H, Shi Q, Gu M, Wan X, Chen Q, Wang Z (2019). Hypoxia-inducible factor 1α (HIF-1α) mediates the epithelial-mesenchymal transition in benign prostatic hyperplasia. Int J Clin Exp Pathol.

[23] Kim HJ, Park JW, Cho YS, Cho CH, Kim JS, Shin HW, Chung DH, Kim SJ, Chun YS (2013). Pathogenic role of HIF-1α in prostate hyperplasia in the presence of chronic inflammation. Biochim Biophys Acta.

[24] Young AP, Schlisio S, Minamishima YA, Zhang Q, Li L, Grisanzio C, Signoretti S, Kaelin WG Jr (2008). VHL loss actuates a HIF-independent senescence programme mediated by Rb and p400. Nat Cell Biol.

[25] Kato H, Inoue T, Asanoma K, Nishimura C, Matsuda T, Wake N (2006). Induction of human endometrial cancer cell senescence through modulation of HIF-1alpha activity by EGLN1. Int J Cancer.

[26] Wu H, Ma H, Wang L, Zhang H, Lu L, Xiao T, Cheng C, Wang P, Yang Y, Wu M (2022). Regulation of lung epithelial cell senescence in smoking-induced COPD/emphysema by microR-125a-5p via Sp1 mediation of SIRT1/HIF-1α. Int J Biol Sci.

[27] Sikora E, Arendt T, Bennett M, Narita M (2011). Impact of cellular senescence signature on ageing research. Ageing Res Rev.

[28] Piret JP, Mottet D, Raes M, Michiels C (2002). CoCl2, a chemical inducer of hypoxia-inducible factor-1, and hypoxia reduce apoptotic cell death in hepatoma cell line HepG2. Ann N Y Acad Sci.

[29] Sun HL, Liu YN, Huang YT, Pan SL, Huang DY, Guh JH, Lee FY, Kuo SC, Teng CM (2007). YC-1 inhibits HIF-1 expression in prostate cancer cells: contribution of Akt/NF-kappaB signaling to HIF-1alpha accumulation during hypoxia. Oncogene.

[30] Smith DK, Hasanali SL, Wang J, Kallifatidis G, Morera DS, Jordan AR, Terris MK, Klaassen Z, Rosser CJ, Lokeshwar BL (2020). Promotion of epithelial hyperplasia by interleukin-8-CXCR axis in human prostate. Prostate.

[31] Fiard G, Stavrinides V, Chambers ES, Heavey S, Freeman A, Ball R, Akbar AN, Emberton M (2021). Cellular senescence as a possible link between prostate diseases of the ageing male. Nat Rev Urol.

[32] Franco AC, Aveleira C, Cavadas C (2022). Skin senescence: mechanisms and impact on whole-body aging. Trends Mol Med.

[33] Kuilman T, Michaloglou C, Mooi WJ, Peeper DS (2010). The essence of senescence. Genes Dev.

[34] Gao HY, Nepovimova E, Heger Z, Valko M, Wu Q, Kuca K, Adam V (2023). Role of hypoxia in cellular senescence. Pharmacol Res.

[35] Ham PB 3rd, Raju R (2017). Mitochondrial function in hypoxic ischemic injury and influence of aging. Prog Neurobiol.

[36] Sengupta T, Abraham G, Xu Y, Clurman BE, Minella AC (2011). Hypoxia-inducible factor 1 is activated by dysregulated cyclin E during mammary epithelial morphogenesis. Mol Cell Biol.

[37] Luczak MW, Zhitkovich A (2017). Nickel-induced HIF-1alpha promotes growth arrest and senescence in normal human cells but lacks toxic effects in transformed cells. Toxicol Appl Pharmacol.

[38] Tsai CC, Chen YJ, Yew TL, Chen LL, Wang JY, Chiu CH, Hung SC (2011). Hypoxia inhibits senescence and maintains mesenchymal stem cell properties through down-regulation of E2A-p21 by HIF-TWIST. Blood.

[39] Salminen A, Kaarniranta K, Kauppinen A (2016). Hypoxia-inducible histone lysine demethylases: impact on the aging process and age-related diseases. Aging Dis.

[40] Molinari E, Bar H, Pyle AM, Patrizio P (2016). Transcriptome analysis of human cumulus cells reveals hypoxia as the main determinant of follicular senescence. Mol Hum Reprod.

[41] Sun L, Li FH, Han C, Wang ZZ, Gao KK, Qiao YB, Ma S, Xie T, Wang J (2021). Alterations in mitochondrial biogenesis and respiratory activity, inflammation of the senescence-associated secretory phenotype, and lipolysis in the perirenal fat and liver of rats following lifelong exercise and detraining. FASEB J.

[42] Wu SK, Ariffin J, Tay SC, Picone R (2023). The variant senescence-associated secretory phenotype induced by centrosome amplification constitutes a pathway that activates hypoxia-inducible factor-1alpha. Aging Cell.

[43] Dimri GP, Lee X, Basile G, Acosta M, Scott G, Roskelley C, Medrano EE, Linskens M, Rubelj I, Pereira-Smith O, Peacocke M, Campisi J (1995). A biomarker that identifies senescent human cells in culture and in aging skin in vivo. Proc Natl Acad Sci U S A.

[44] Groppo R, Richter JD (2011). CPEB control of NF-κB nuclear localization and interleukin-6 production mediates cellular senescence. Mol Cell Biol.

[45] Jung YH, Chae CW, Chang HS, Choi GE, Lee HJ, Han HJ (2022). Silencing SIRT5 induces the senescence of UCB-MSCs exposed to TNF-α by reduction of fatty acid β-oxidation and anti-oxidation. Free Radic Biol Med.

[46] Li J, Wang X, Nepovimova E, Wu Q, Kuca K (2024). Deoxynivalenol induces cell senescence in RAW264.7 macrophages via HIF-1α-mediated activation of the p53/p21 pathway. Toxicology.

[47] Kiviaho A, Eerola SK, Kallio HML (2024). Single cell and spatial transcriptomics highlight the interaction of club-like cells with immunosuppressive myeloid cells in prostate cancer. Nat Commun.

[48] Li Z, Wang X, Liu Z, Li S, Zhang Z, Huang C, Liu Y, Tang X, Zhang J, Zhou P (2025). Single-cell sequencing reveals that CD4+ T cells eliminate senescent prostate epithelium to delay progression of benign prostatic hyperplasia. Aging Cell.

