# Supplementary material for: Spatial and single-cell transcriptomics reveal a HIF-1α/NF-κB-driven hypoxia-induced senescence axis in BPH epithelium
Source: Int J Biol Sci. 2026 Jun 10;22(11):6149–76. doi: 10.7150/ijbs.130242 (PMC13282791; doi:10.7150/ijbs.130242)
Supplement: Supplementary file 1 — Supplementary figures. [file ijbsv22p6149s1.pdf]

Supplementary Figures

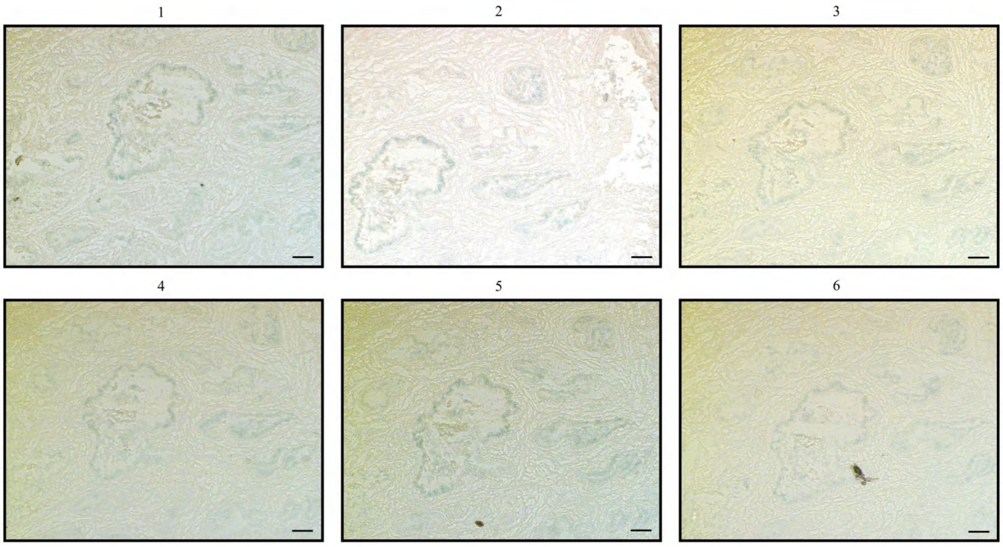

Supplementary Figure S1. Serial-section SA-β-gal staining in BPH tissues.

Serial frozen sections were used to assess the consistency of SA-β-gal staining within the same glandular structure. SA-β-gal staining was generally consistent within the same gland. Scale bar = 100 μm.

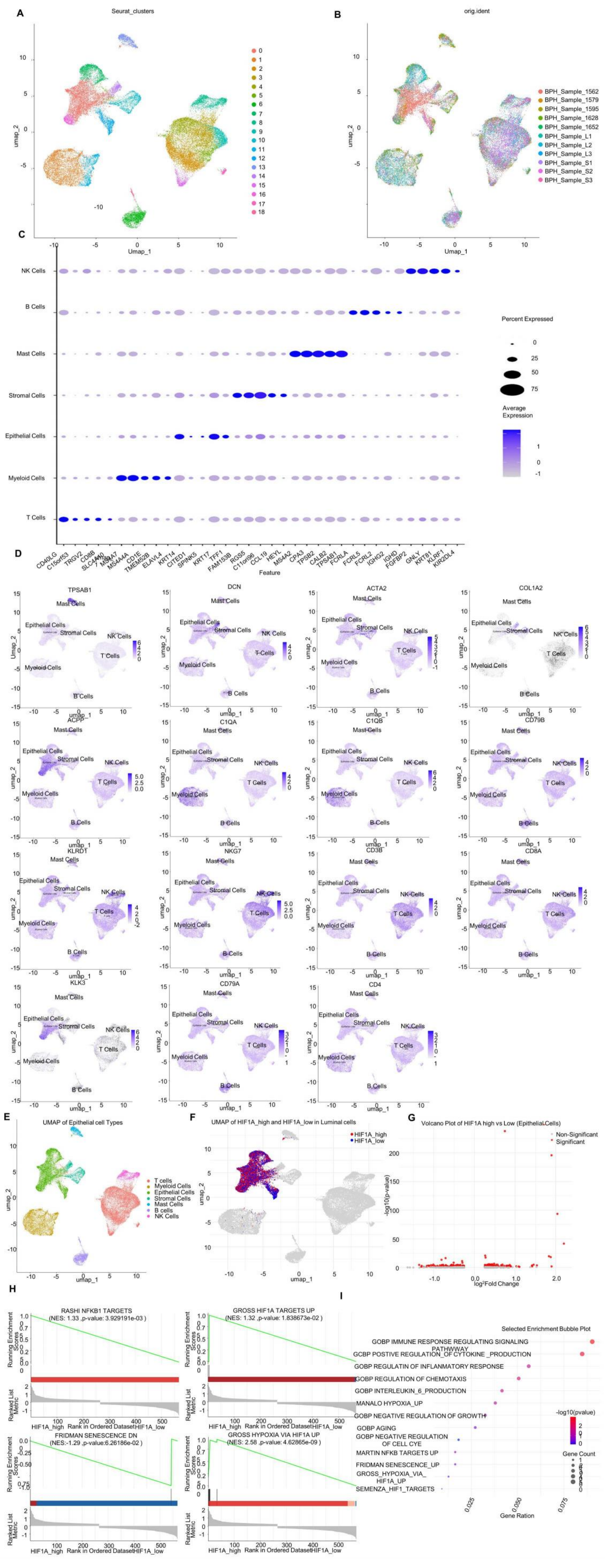

A
Seurat_clusters
B
orig.ident
BPH_Sample_1562
BPH_Sample_1579
BPH_Sample_1595
BPH_Sample_1628
BPH_Sample_1652
BPH_Sample_L1
BPH_Sample_L2
BPH_Sample_L3
BPH_Sample_S1
BPH_Sample_S2
BPH_Sample_S3
Umap_1
umap_2
C
NK Cells
B Cells
Mast Cells
Stromal Cells
Epithelial Cells
Myeloid Cells
T Cells
Percent Expressed
Average Expression
Feature
D
TPSAB1
DCN
ACTA2
COL1A2
ACPP
C1QA
C1QB
CD79B
KLRD1
NKG7
CD3E
CD8A
KLK3
CD79A
CD4
E
UMAP of Epithelial cell Types
T cells
Myeloid Cells
Epithelial Cells
Stromal Cells
Mast Cells
B cells
NK Cells
F
UMAP of HIF1A_high and HIF1A_low in Luminal cells
HIF1A_high
HIF1A_low
G
Volcano Plot of HIF1A high vs Low (Epithelial Cells)
Non-Significant
Significant
-log10(p-value)
log2Fold Change
H
RASHI NFKB1 TARGETS
(NES: 1.33 ,p-value: 3.929191e-03)
GROSS HIF1A TARGETS UP
(NES: 1.32 ,p-value: 1.838673e-02)
FRIDMAN SENESCENCE DN
(NES:-1.29 ,p-value:6.26186e-02)
GROSS HYPOXIA VIA HIF1A UP
(NES: 2.58 ,p-value: 4.62865e-09)
Running Enrichment Scores
Ranked List Metric
HIF1A_high Rank in Ordered Dataset HIF1A_low
I
Selected Enrichment Bubble Plot
GOBP IMMUNE RESPONSE REGULATING SIGNALING PATHWAY
GCBP POSTIVE REGULATION_OF CYTOKINE _PRODUCTION
GOBP REGULATIN OF INFLANMATORY RESPONSE
GOBP REGULATION OF CHEMOTAXIS
GOBP INTERLEUKIN_6_PRODUCTION
MANALO HYPOXIA_UP
GOBP NEGATIVE REGULATION OF GROWTH
GOBP AGING
GOBP NEGATIVE REGULATION OF CELL CYE
MARTIN NFKB TARGETS UP
FRIDMAN SENESCENCE_UP
GROSS_HYPOXIA_VIA_HIF1A_UP
SEMENZA_HIF1_TARGETS
-log10(pvalue)
Gene Count
Gene Ration

Supplementary Figure S2. Single-cell transcriptomic analysis of prostate tissues from BPH patients.

(A) UMAP plot displaying clusters identified using the Seurat algorithm, with each color representing a distinct cell type in the prostate transitional zone.

(B) UMAP plot showing cells color-coded by patient origin, highlighting inter-sample variability and integration across samples.

(C) Dot plot showing the expression of marker genes used to annotate major cell types, including *DCN*, *ACTA2*, and *COL1A2* for stromal cells; *KLK3* and *ACPP* for epithelial cells; *C1QA* and *C1QB* for myeloid cells; *CD79A* and *CD79B* for B cells; *KLRD1* and *NKG7* for NK cells; and *CD3D*, *CD4*, and *CD8A* for T cells.

(D) Feature plots of representative marker-gene expression confirming the identity of each cell lineage in the UMAP visualization.

(E) UMAP projection of the six major cell types identified in the human BPH scRNA-seq dataset, including T cells, myeloid cells, epithelial cells, stromal cells, NK cells, and B cells.

(F) UMAP projection of epithelial cells stratified into HIF-1α pathway-high and HIF-1α pathway-low groups based on GSVA scores for the MSigDB gene set GROSS_HYPOXIA_VIA_HIF1A_UP, highlighting the enrichment of Luminal cells in the HIF-1α pathway-high group.

(G) Volcano plot showing DEGs between HIF-1α pathway-high and HIF-1α pathway-low epithelial cells. Red dots indicate significantly upregulated genes, and blue dots indicate significantly downregulated genes.

(H) GSEA plots showing pathways enriched in HIF-1α pathway-high epithelial cells, including hypoxia-related pathways and NF-κB signaling.

(I) Bubble plot analysis of hypoxia- and senescence-related pathways significantly enriched in HIF-1α pathway-high epithelial cells.

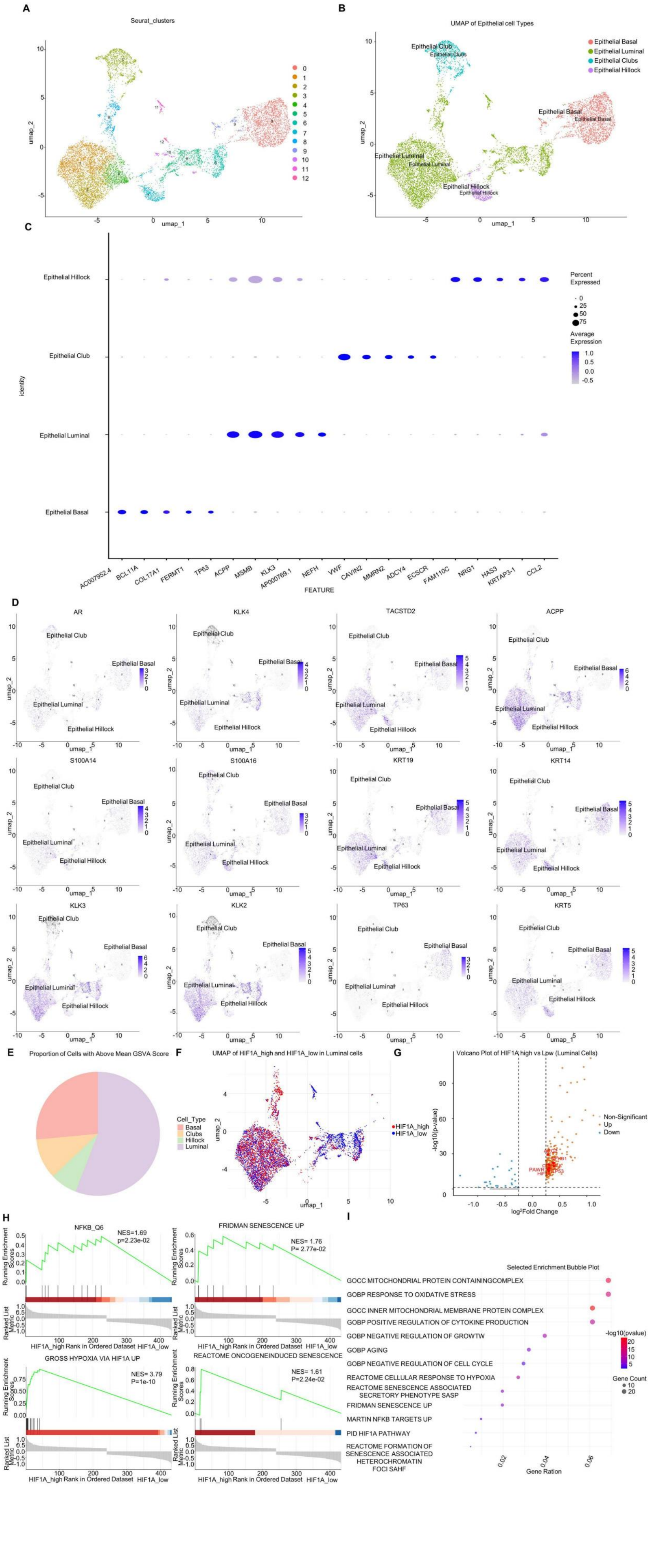
A
Seurat_clusters
B
UMAP of Epithelial cell Types
Epithelial Basal
Epithelial Luminal
Epithelial Clubs
Epithelial Hillock
C
Percent Expressed
Average Expression
Epithelial Hillock
Epithelial Club
Epithelial Luminal
Epithelial Basal
identity
AC007952.4
BCL11A
COL17A1
FERMT1
TP63
ACPP
MSMB
KLK3
AP000769.1
NEFH
VWF
CAVIN2
MMRN2
ADCY4
ECSCR
FAM110C
NRG1
HAS3
KRTAP3-1
CCL2
FEATURE
D
AR
KLK4
TACSTD2
ACPP
S100A14
S100A16
KRT19
KRT14
KLK3
KLK2
TP63
KRT5
E
Proportion of Cells with Above Mean GSVA Score
Cell_Type
Basal
Clubs
Hillock
Luminal
F
UMAP of HIF1A_high and HIF1A_low in Luminal cells
HIF1A_high
HIF1A_low
G
Volcano Plot of HIF1A high vs Low (Luminal Cells)
Non-Significant
Up
Down
-log10(p-value)
log2Fold Change
H
NFKB_Q6
NES=1.69
p=2.23e-02
FRIDMAN SENESCENCE UP
NES= 1.76
P= 2.77e-02
GROSS HYPOXIA VIA HIF1A UP
NES= 3.79
P=1e-10
REACTOME ONCOGENEINDUCED SENESCENCE
NES= 1.61
P=2.24e-02
Running Enrichment Scores
Ranked List Metric
HIF1A_high Rank in Ordered Dataset HIF1A_low
I
Selected Enrichment Bubble Plot
GOCC MITOCHONDRIAL PROTEIN CONTAININGCOMPLEX
GOBP RESPONSE TO OXIDATIVE STRESS
GOCC INNER MITOCHONDRIAL MEMBRANE PROTEIN COMPLEX
GOBP POSITIVE REGULATION OF CYTOKINE PRODUCTION
GOBP NEGATIVE REGULATION OF GROWTW
GOBP AGING
GOBP NEGATIVE REGULATION OF CELL CYCLE
REACTOME CELLULAR RESPONSE TO HYPOXIA
REACTOME SENESCENCE ASSOCIATED SECRETORY PHENOTYPE SASP
FRIDMAN SENESCENCE UP
MARTIN NFKB TARGETS UP
PID HIF1A PATHWAY
REACTOME FORMATION OF SENESCENCE ASSOCIATED HETEROCHROMATIN FOCI SAHF
-log10(pvalue)
Gene Count
Gene Ration

Supplementary Figure S3. Detailed analysis of epithelial cells in the transitional zone of prostate tissues from BPH patients.

(A) UMAP plot showing clustering of 10,727 epithelial cells from BPH prostate tissues, classified into four epithelial subtypes: Luminal, Basal, Hillock, and Club cells.

(B) Dot plot showing the expression of key marker genes distinguishing the four epithelial subtypes.

(C) UMAP plot of epithelial subtypes.

(D) Feature plots showing the expression patterns of key marker genes across epithelial subtypes, including *AR*, *KLK4*, *TACSTD2*, *ACPP*, *S100A14*, *S100A16*, and *KRT19*.

(E) Pie chart showing the proportion of epithelial subtypes with above-mean GSVA scores for the hypoxia/HIF-1α-related signature.

(F) UMAP projection of epithelial cells stratified into HIF-1α pathway-high and HIF-1α pathway-low groups based on GSVA scores for the MSigDB gene set GROSS_HYPOXIA_VIA_HIF1A_UP, highlighting the predominance of Luminal cells in the HIF-1α pathway-high group.

(G) Volcano plot showing DEGs between HIF-1α pathway-high and HIF-1α pathway-low epithelial cells. Representative senescence-associated and pathway-related genes selected for cross-dataset comparison, including *ARG2*, *PAWR*, *TP53*, *HIF1A*, *NFKB1*, and *CDKN1A*, are labeled.

(H) GSEA plots showing significant enrichment of hypoxia-, NF-κB-, and senescence-related pathways, including NFKB_Q6, FRIDMAN_SENESCENCE_UP, GROSS_HYPOXIA_VIA_HIF1A_UP, and REACTOME_ONCOGENE_INDUCED_SENESCENCE.

(I) Selected enrichment bubble plot showing gene ratio and statistical significance of

pathways related to mitochondrial function, oxidative stress, cytokine production, growth regulation, and senescence in epithelial cell subtypes.

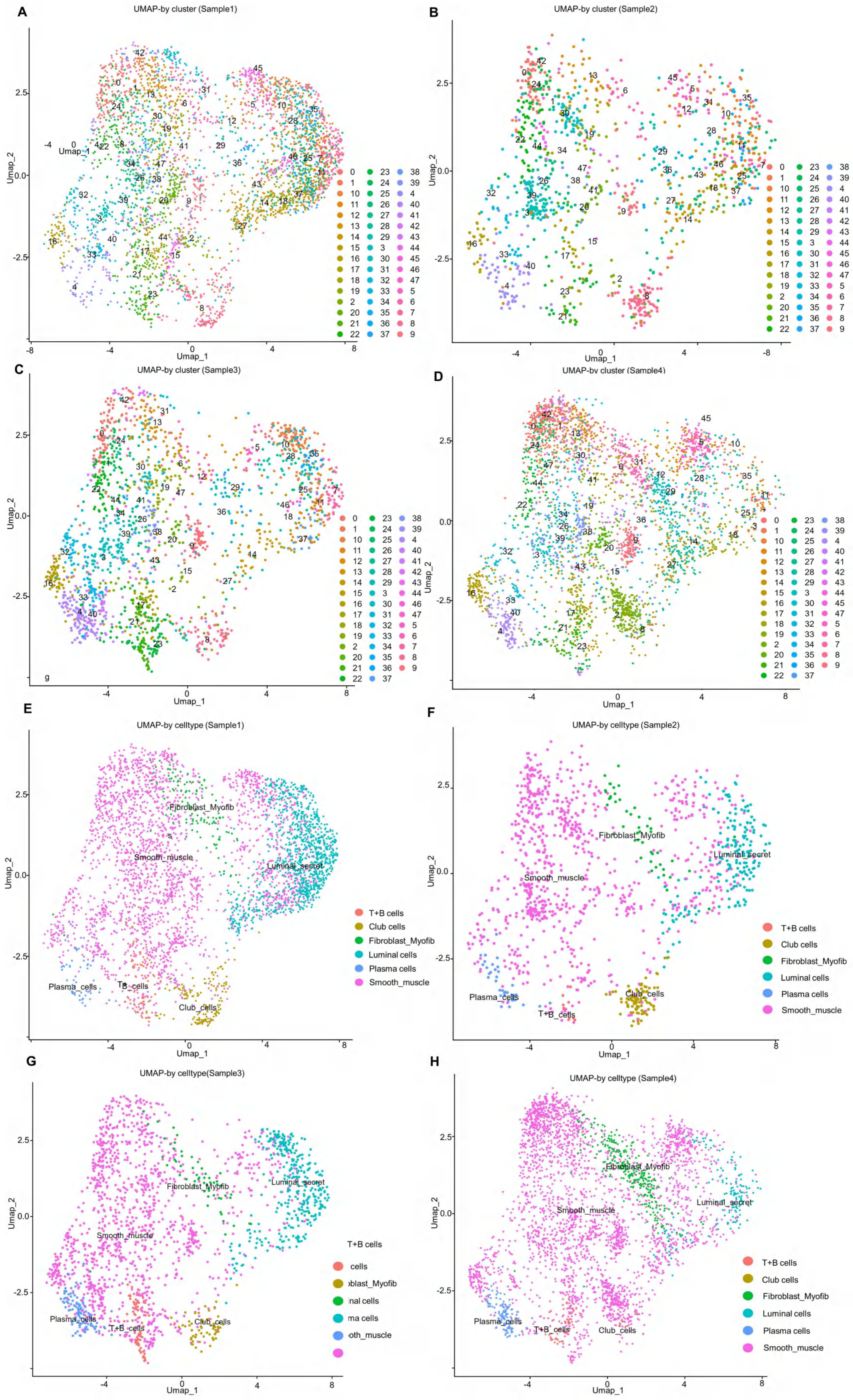
A
UMAP-by cluster (Sample1)
B
UMAP-by cluster (Sample2)
C
UMAP-by cluster (Sample3)
D
UMAP-by cluster (Sample4)
E
UMAP-by celltype (Sample1)
F
UMAP-by celltype (Sample2)
G
UMAP-by celltype(Sample3)
H
UMAP-by celltype (Sample4)
Umap_1
Umap_2
Fibroblast_Myofib
Smooth_muscle
Luminal_secret
Plasma_cells
T+B_cells
Club_cells
T+B cells
Club cells
Fibroblast_Myofib
Luminal cells
Plasma cells
Smooth_muscle

Supplementary Figure S4. Sample-wise spatial transcriptomic analysis and reference-based spot annotation of BPH tissues.

(A–D) UMAP embeddings of spatial transcriptomic spots from four BPH samples, analyzed separately to illustrate sample-level transcriptomic heterogeneity before cross-sample integration.

(E–H) Reference-based annotation of spatial spots following label transfer from the scRNA-seq reference, identifying six major dominant spot identities or cell states: Smooth_muscle cells, Luminal cells, Fibroblast_Myofib cells, Plasma cells, Club cells, and T+B cells.

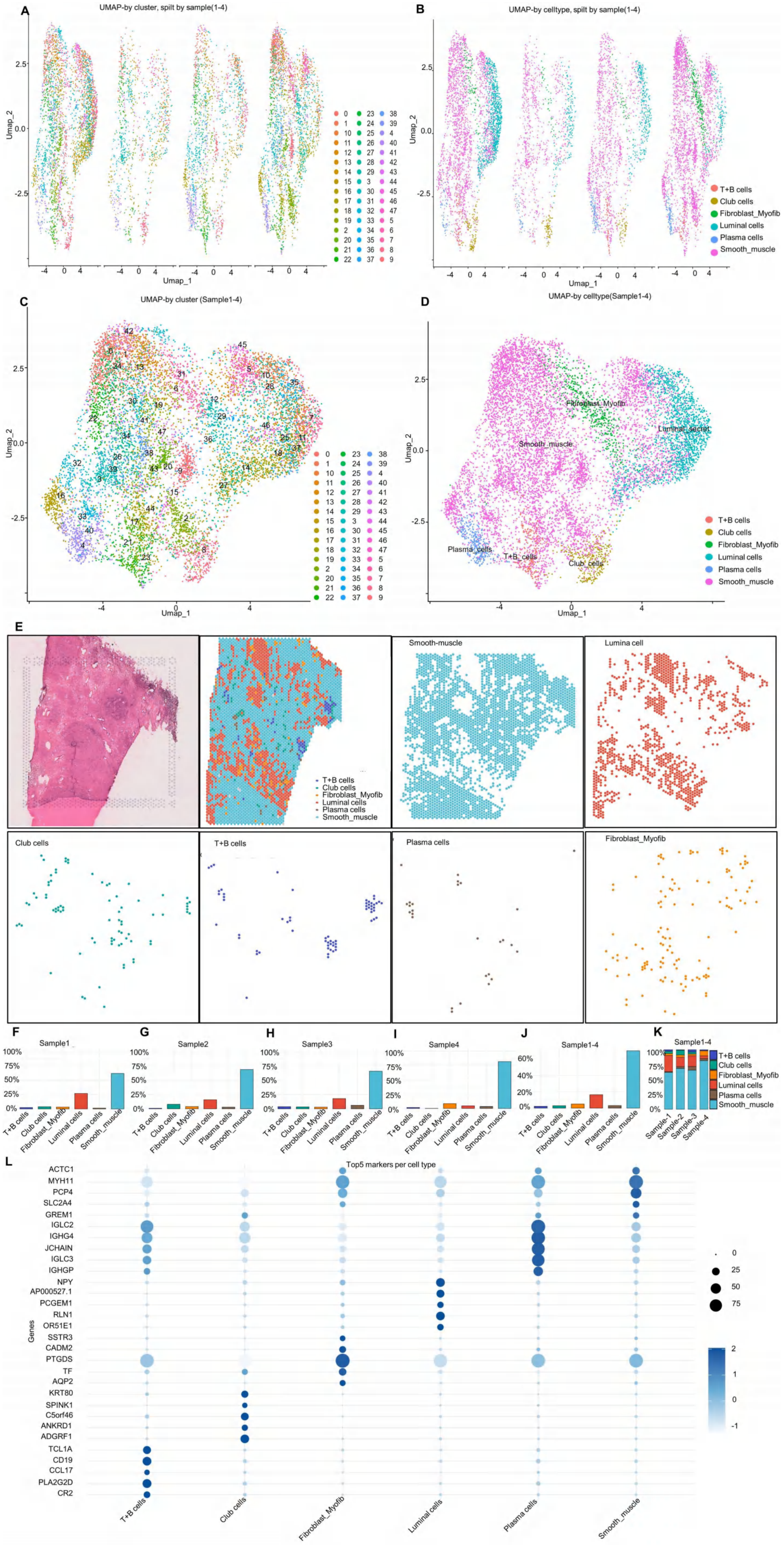
A
UMAP-by cluster, spilt by sample(1-4)
B
UMAP-by celltype, spilt by sample(1-4)
C
UMAP-by cluster (Sample1-4)
D
UMAP-by celltype(Sample1-4)
Umap_1
Umap_2
T+B cells
Club cells
Fibroblast_Myofib
Luminal cells
Plasma cells
Smooth_muscle
Luminal_secret
Smooth_muscle
Plasma_cells
T+B_cells
Club_cells
E
Smooth-muscle
Lumina cell
Club cells
T+B cells
Plasma cells
Fibroblast_Myofib
F
Sample1
G
Sample2
H
Sample3
I
Sample4
J
Sample1-4
K
Sample1-4
Sample-1
Sample-2
Sample-3
Sample-4
L
Top5 markers per cell type
ACTC1
MYH11
PCP4
SLC2A4
GREM1
IGLC2
IGHG4
JCHAIN
IGLC3
IGHGP
NPY
AP000527.1
PCGEM1
RLN1
OR51E1
SSTR3
CADM2
PTGDS
TF
AQP2
KRT80
SPINK1
C5orf46
ANKRD1
ADGRF1
TCL1A
CD19
CCL17
PLA2G2D
CR2
Genes

Supplementary Figure S5. Integrated spatial transcriptomic atlas and compositional features of BPH tissues.

(A–D) Integrated UMAP plots of spatial transcriptomic spots from all four BPH samples, colored by sample origin (A, B) and by transferred dominant spot identity/cell state (C, D), showing effective cross-sample integration.

(E) Representative H&E-stained tissue section overlaid with transferred spot annotations, illustrating the spatial distribution of major lineages/cell states in BPH tissue.

(F–I) Proportions of major transferred spot identities/cell states in each individual spatial transcriptomic sample.

(J–K) Overall composition of transferred spot identities/cell states across all four samples, shown as a bar plot (J) and stacked bar plot (K).

(L) Supportive summary of selected marker-gene expression patterns across major transferred spatial spot identities/cell states. Detailed marker-expression validation for individual annotated populations is shown in Figs. S6–S7.

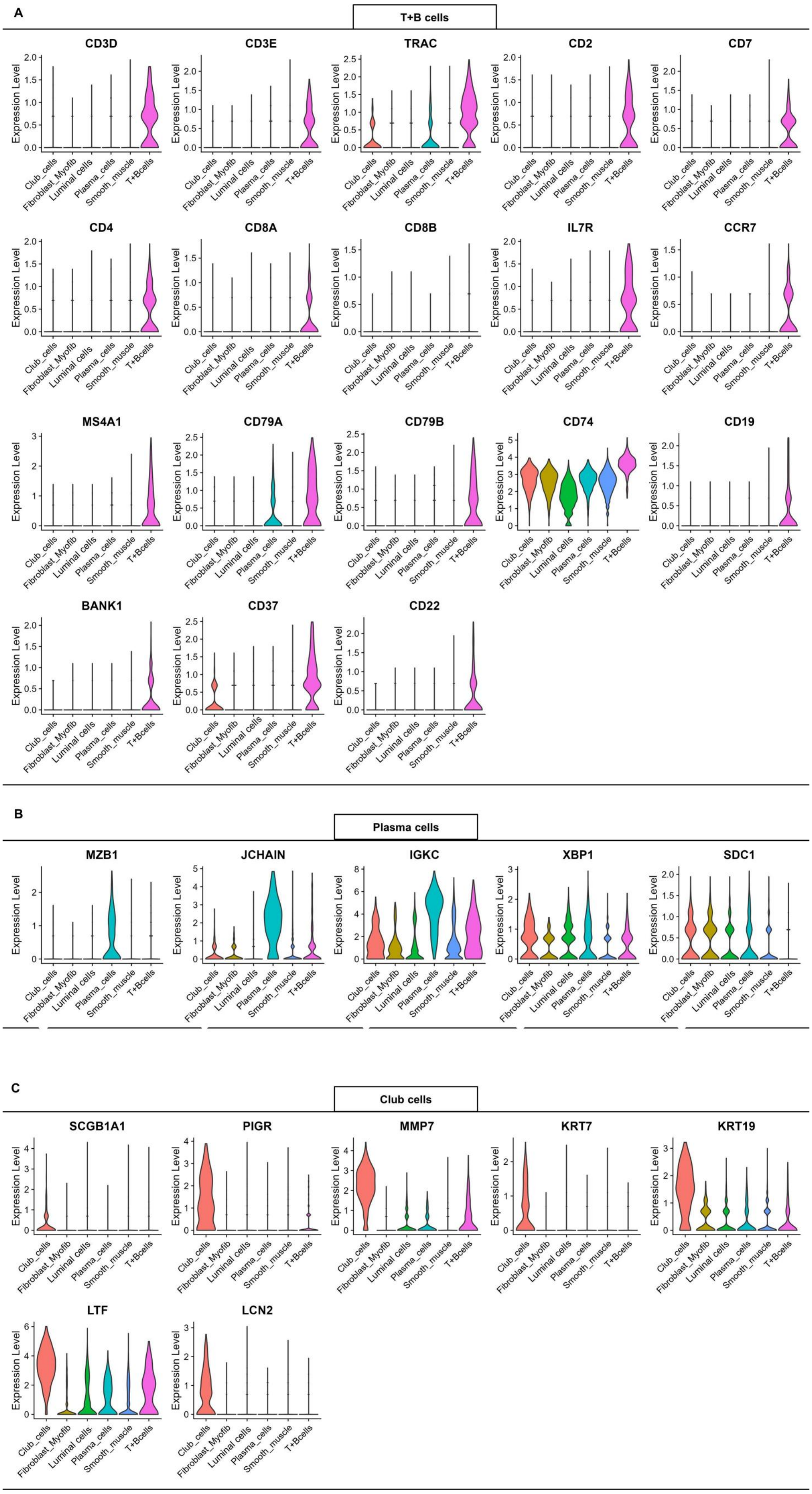
A
T+B cells
CD3D
CD3E
TRAC
CD2
CD7
CD4
CD8A
CD8B
IL7R
CCR7
MS4A1
CD79A
CD79B
CD74
CD19
BANK1
CD37
CD22
B
Plasma cells
MZB1
JCHAIN
IGKC
XBP1
SDC1
C
Club cells
SCGB1A1
PIGR
MMP7
KRT7
KRT19
LTF
LCN2
Expression Level
Club_cells
Fibroblast_Myofib
Luminal cells
Plasma_cells
Smooth_muscle
T+Bcells

Supplementary Figure S6. Representative marker-gene expression in T+B, Plasma, and Club cell populations.

(A) Violin plots showing expression of canonical T- and B-cell markers in the T+B cell cluster, including *CD3D* and *CD4* as T-cell markers and *CD19* as a B-cell marker.
(B) Violin plots showing expression of plasma cell markers *MZB1*, *JCHAIN*, and *IGKC*, highlighting their selective enrichment in the Plasma cell population.
(C) Violin plots showing expression of representative Club cell markers *SCGB1A1*, *PIGR*, and *KRT7*, demonstrating their preferential expression in Club cells relative to other lineages.

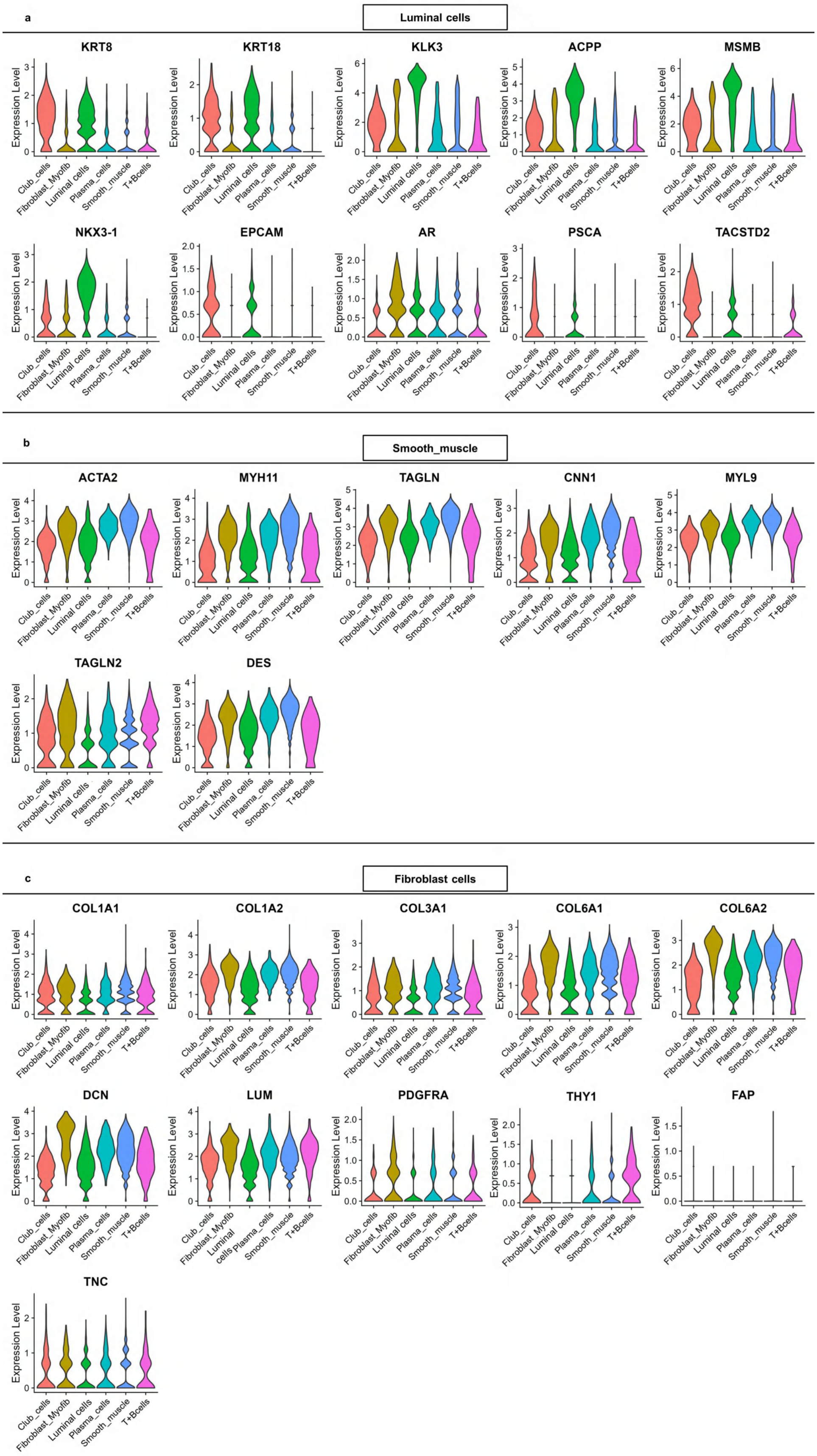

a
Luminal cells
KRT8
KRT18
KLK3
ACPP
MSMB
NKX3-1
EPCAM
AR
PSCA
TACSTD2
b
Smooth_muscle
ACTA2
MYH11
TAGLN
CNN1
MYL9
TAGLN2
DES
c
Fibroblast cells
COL1A1
COL1A2
COL3A1
COL6A1
COL6A2
DCN
LUM
PDGFRA
THY1
FAP
TNC
Expression Level
Club_cells
Fibroblast_Myofib
Luminal cells
Plasma_cells
Smooth_muscle
T+Bcells

Supplementary Figure S7. Representative marker-gene expression in Luminal, Smooth_muscle, and Fibroblast_Myofib populations.

(A) Violin plots showing expression of luminal epithelial markers *KRT8*, *KLK3*, and *ACPP* in the Luminal cell population.

(B) Violin plots showing expression of smooth muscle markers *ACTA2*, *MYH11*, and *TAGLN* in the Smooth_muscle population.

(C) Violin plots showing expression of fibroblast/myofibroblast-associated markers *COL1A1*, *DCN*, and *PDGFRA* in the Fibroblast_Myofib population.

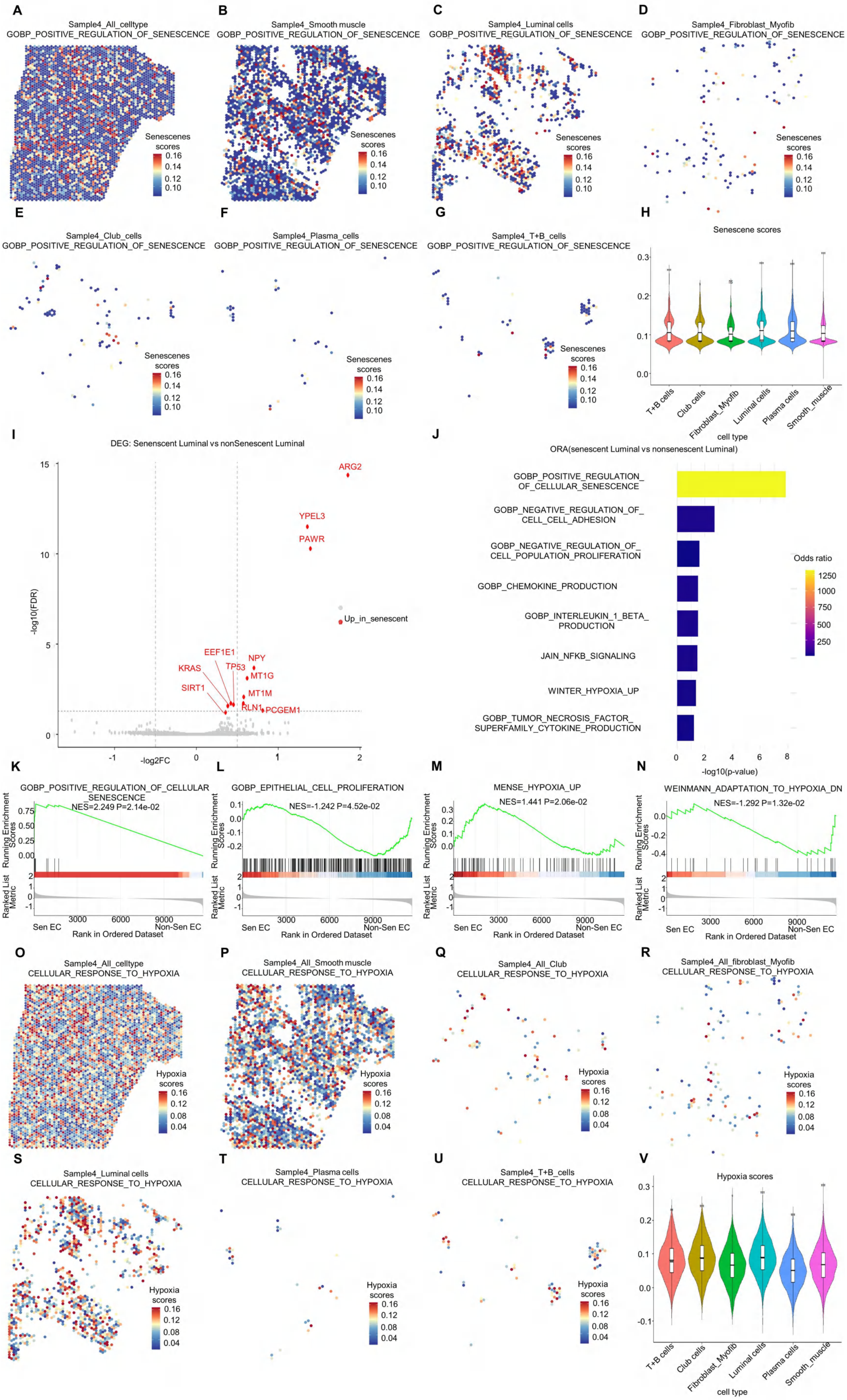
A
Sample4_All_celltype
GOBP_POSITIVE_REGULATION_OF_SENESCENCE
B
Sample4_Smooth muscle
GOBP_POSITIVE_REGULATION_OF_SENESCENCE
C
Sample4_Luminal cells
GOBP_POSITIVE_REGULATION_OF_SENESCENCE
D
Sample4_Fibroblast_Myofib
GOBP_POSITIVE_REGULATION_OF_SENESCENCE
Senescenes scores
0.16
0.14
0.12
0.10
E
Sample4_Club_cells
GOBP_POSITIVE_REGULATION_OF_SENESCENCE
F
Sample4_Plasma_cells
GOBP_POSITIVE_REGULATION_OF_SENESCENCE
G
Sample4_T+B_cells
GOBP_POSITIVE_REGULATION_OF_SENESCENCE
H
Senescene scores
T+B cells
Club cells
Fibroblast_Myofib
Luminal cells
Plasma cells
Smooth_muscle
cell type
I
DEG: Senenscent Luminal vs nonSenescent Luminal
-log10(FDR)
-log2FC
ARG2
YPEL3
PAWR
Up_in_senescent
EEF1E1
NPY
KRAS
TP53
MT1G
SIRT1
MT1M
RLN1
PCGEM1
J
ORA(senescent Luminal vs nonsenescent Luminal)
GOBP_POSITIVE_REGULATION_OF_CELLULAR_SENESCENCE
GOBP_NEGATIVE_REGULATION_OF_CELL_CELL_ADHESION
GOBP_NEGATIVE_REGULATION_OF_CELL_POPULATION_PROLIFERATION
GOBP_CHEMOKINE_PRODUCTION
GOBP_INTERLEUKIN_1_BETA_PRODUCTION
JAIN_NFKB_SIGNALING
WINTER_HYPOXIA_UP
GOBP_TUMOR_NECROSIS_FACTOR_SUPERFAMILY_CYTOKINE_PRODUCTION
Odds ratio
1250
1000
750
500
250
-log10(p-value)
K
GOBP_POSITIVE_REGULATION_OF_CELLULAR_SENESCENCE
NES=2.249 P=2.14e-02
L
GOBP_EPITHELIAL_CELL_PROLIFERATION
NES=-1.242 P=4.52e-02
M
MENSE_HYPOXIA_UP
NES=1.441 P=2.06e-02
N
WEINMANN_ADAPTATION_TO_HYPOXIA_DN
NES=-1.292 P=1.32e-02
Running Enrichment Scores
Ranked List Metric
Sen EC
Non-Sen EC
Rank in Ordered Dataset
O
Sample4_All_celltype
CELLULAR_RESPONSE_TO_HYPOXIA
P
Sample4_All_Smooth muscle
CELLULAR_RESPONSE_TO_HYPOXIA
Q
Sample4_All_Club
CELLULAR_RESPONSE_TO_HYPOXIA
R
Sample4_All_fibroblast_Myofib
CELLULAR_RESPONSE_TO_HYPOXIA
Hypoxia scores
0.16
0.12
0.08
0.04
S
Sample4_Luminal cells
CELLULAR_RESPONSE_TO_HYPOXIA
T
Sample4_Plasma cells
CELLULAR_RESPONSE_TO_HYPOXIA
U
Sample4_T+B_cells
CELLULAR_RESPONSE_TO_HYPOXIA
V
Hypoxia scores
T+B cells
Club cells
Fibroblast_Myofib
Luminal cells
Plasma cells
Smooth_muscle
cell type

Supplementary Figure S8. Spatial transcriptomic analysis of senescence- and hypoxia-associated transcriptional programs in human BPH prostate tissues.

(A–G) Spatial feature maps showing UCell scores for GOBP_POSITIVE_REGULATION_OF_CELLULAR_SENESCENCE across all spots and major transferred spot identities/cell states in representative spatial transcriptomic sections. The color scale indicates senescence-associated transcriptional scores.

(H) Violin plots with embedded boxplots showing senescence-associated UCell scores across major transferred spot identities/cell states. The embedded boxplots indicate the median and interquartile range. Luminal-dominant spots showed relatively high senescence-associated transcriptional scores compared with other major spot identities/cell states.

(I) Volcano plot showing differentially expressed genes between senescence-score-high and senescence-score-low Luminal-dominant spots. Representative upregulated genes in senescence-score-high Luminal-dominant spots, including ARG2, YPEL3, PAWR, KRAS, EEF1E1, and TP53, are labeled.

(J) Over-representation analysis of genes upregulated in senescence-score-high Luminal-dominant spots, showing enrichment of senescence-, inflammatory cytokine-, NF-κB-, and hypoxia-related biological processes or pathways.

(K–N) Gene set enrichment analysis of ranked genes comparing senescence-score-high and senescence-score-low Luminal-dominant spots. Senescence-associated and hypoxia-related signatures were positively enriched in senescence-score-high Luminal-dominant spots, whereas epithelial-cell proliferation and hypoxia-adaptation-down signatures showed negative enrichment.

(O–U) Spatial feature maps showing UCell scores for REACTOME_CELLULAR_RESPONSE_TO_HYPOXIA across all spots and major transferred spot identities/cell states in representative spatial transcriptomic sections. The color scale indicates hypoxia-response scores.

(V) Violin plots with embedded boxplots showing hypoxia-response UCell scores across major transferred spot identities/cell states. The embedded boxplots indicate the median and interquartile range.

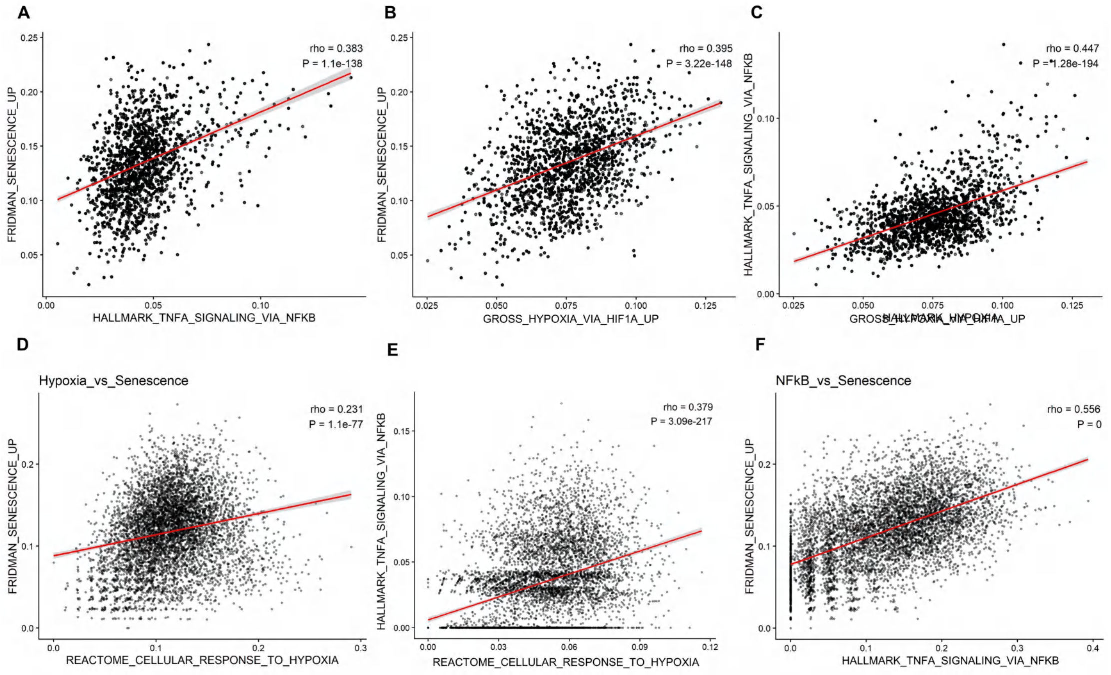

Supplementary Figure S9. Correlation analysis of hypoxia-, NF-κB-, and senescence-associated pathway scores in single-cell and spatial transcriptomic datasets.

(A–C) Scatter plots showing pairwise correlations among pathway scores in epithelial cells from the human BPH scRNA-seq dataset. Pathway activity was scored using gene sets applied in the single-cell analysis, including GROSS_HYPOXIA_VIA_HIF1A_UP for hypoxia/HIF-1α pathway activity, HALLMARK_TNFA_SIGNALING_VIA_NFKB for NF-κB-related inflammatory signaling, and FRIDMAN_SENESCENCE_UP for cellular senescence. Positive correlations were observed between NF-κB and senescence scores (A), hypoxia/HIF-1α and senescence scores (B), and hypoxia/HIF-1α and NF-κB scores (C).

(D–F) Scatter plots showing pairwise correlations among pathway scores in spatial

transcriptomic spots from human BPH tissues. Spatial pathway activity was scored using REACTOME_CELLULAR_RESPONSE_TO_HYPOXIA for hypoxia response, HALLMARK_TNFA_SIGNALING_VIA_NFKB for NF-κB signaling, and GOBP_POSITIVE_REGULATION_OF_CELLULAR_SENESCENCE for senescence-associated transcriptional activity. Positive correlations were observed between hypoxia and senescence scores (D), hypoxia and NF-κB scores (E), and NF-κB and senescence scores (F). Red lines indicate fitted linear trends. Correlations were assessed using Spearman ’ s rank correlation analysis; rho and P values are shown in each panel. For the spatial dataset, pathway scores represent spot-level transcriptional activity and should not be interpreted as single-cell state assignments.

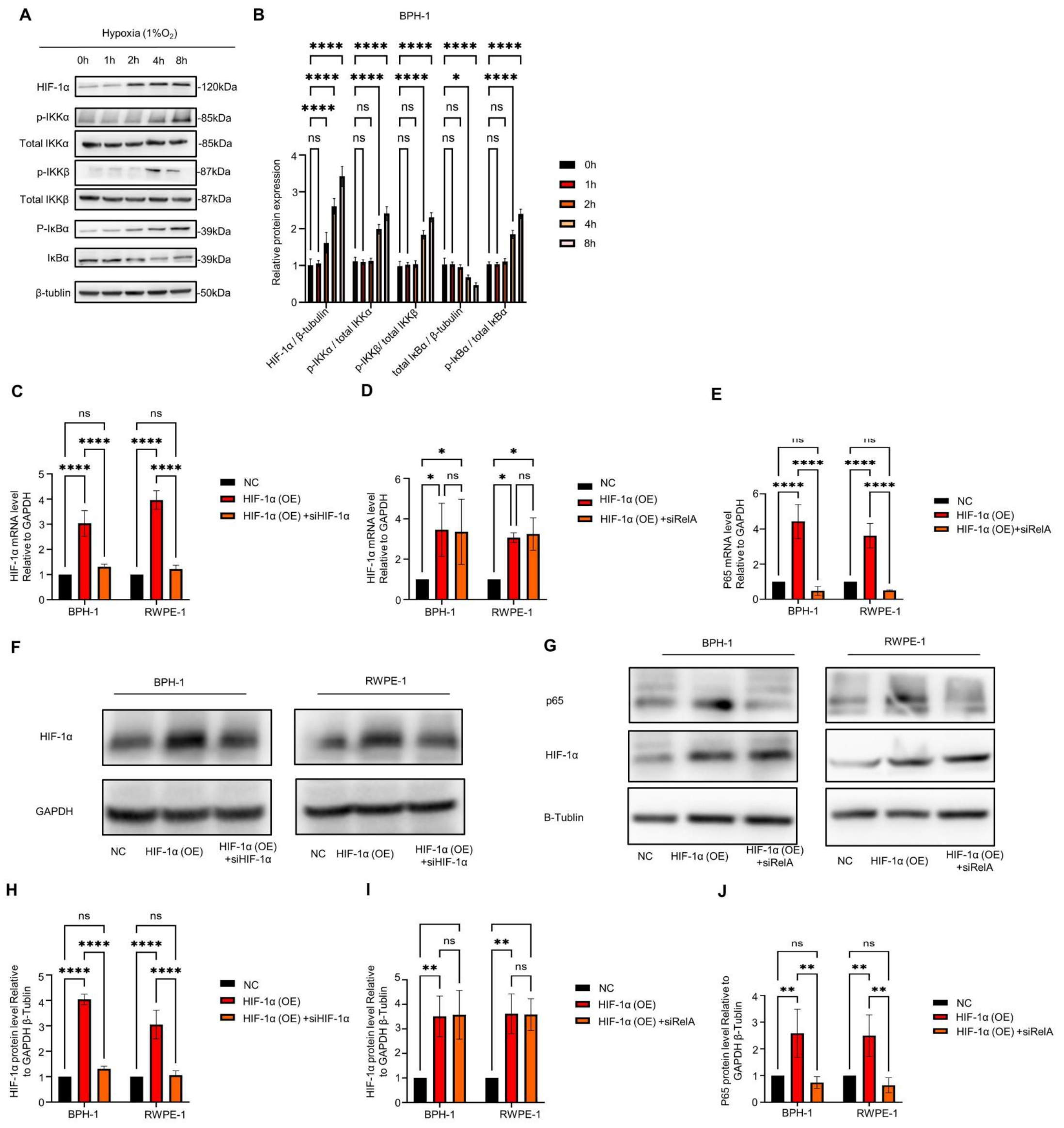

Supplementary Figure S10. Time-course analysis of HIF-1α accumulation and IKK/IκBα phosphorylation under hypoxia and validation of HIF-1α/*RELA* genetic manipulation efficiency.

(A) Western blot analysis of HIF-1α, p-IKKα, total IKKα, p-IKKβ, total IKKβ, p-IκBα, total IκBα, and β-tubulin in BPH-1 cells exposed to 1% O_2 hypoxia for the indicated time points. Groups 1–5 represent 0, 1, 2, 4, and 8 h of hypoxia exposure, respectively.

(B) Quantification of the indicated proteins in (A). HIF-1α and total IκBα were normalized to β-tubulin. p-IKKα, p-IKKβ, and p-IκBα were normalized to total IKKα, total IKKβ, and total IκBα, respectively. All values were expressed relative to group 1.

(C) qRT-PCR analysis of *HIF1A* mRNA levels in BPH-1 and RWPE-1 cells after HIF-1α overexpression with or without si*HIF1A* co-transfection.

(D, E) qRT-PCR analysis of *HIF1A* (D) and *RELA* (E) mRNA levels in BPH-1 and RWPE-1 cells after HIF-1α overexpression with or without si*RELA* co-transfection.

(F, H) Western blot analysis (F) and quantification (H) of HIF-1α protein levels in BPH-1 and RWPE-1 cells after HIF-1α overexpression with or without si*HIF1A* co-transfection. GAPDH was used as the loading control.

(G, I, J) Western blot analysis (G) and quantification of HIF-1α (I) and p65 (J) protein levels in BPH-1 and RWPE-1 cells after HIF-1α overexpression with or without si*RELA* co-transfection. β-tubulin was used as the loading control. Data are presented as the mean ± SD. ns, not significant; $P < 0.05$; $P < 0.01$; $P < 0.001$; $P < 0.0001$.

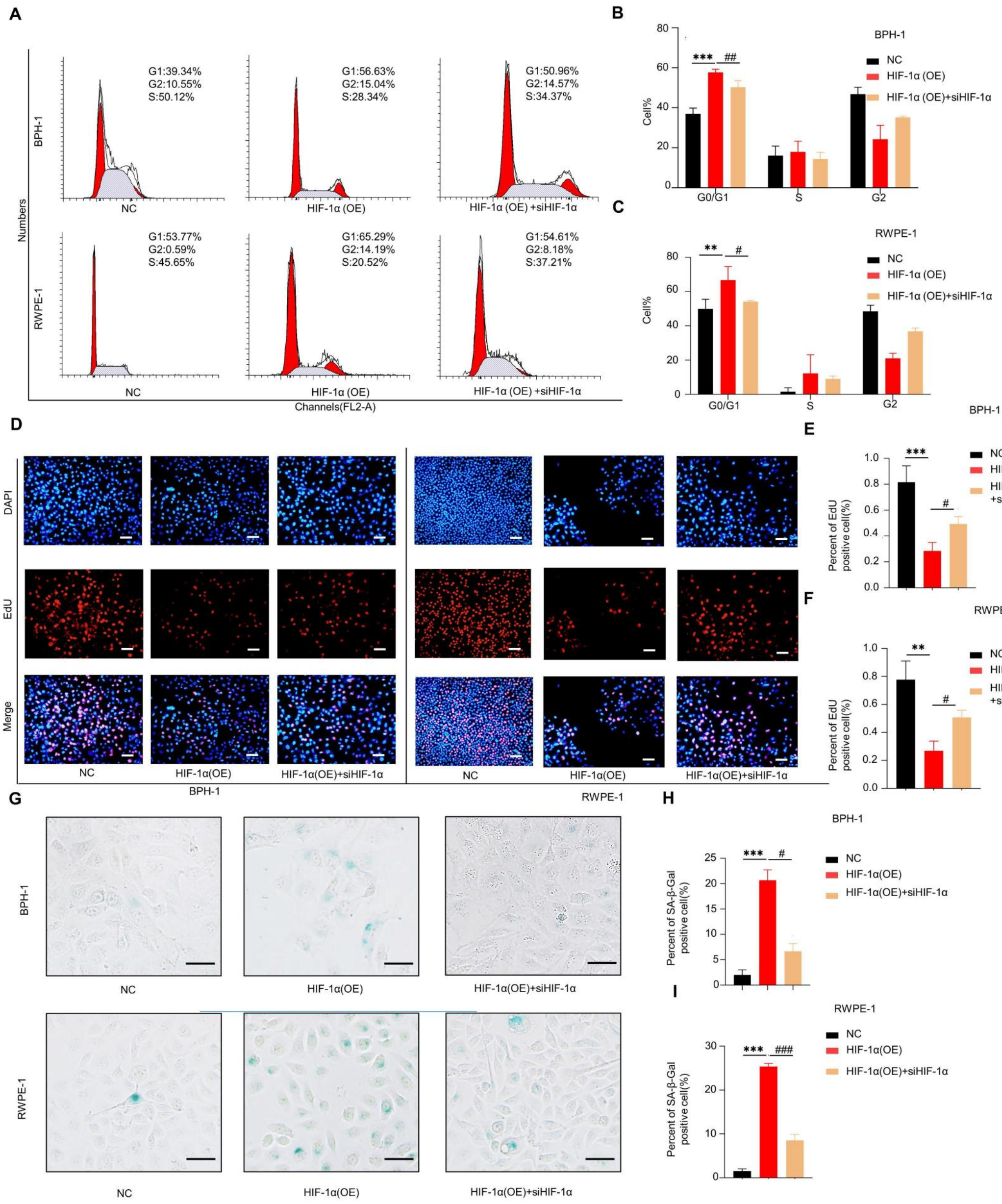

Supplementary Figure S11. HIF-1α overexpression induces senescence-associated phenotypes in prostate epithelial cells.

(A–C) Flow cytometric analysis showing the cell-cycle distribution of BPH-1 and RWPE-1 cells in the control, HIF-1α overexpression, and HIF-1α overexpression + si*HIF1A* groups.

(D–F) EdU assays showing the proliferation of BPH-1 and RWPE-1 cells in the control, HIF-1α overexpression, and HIF-1α overexpression + si*HIF1A* groups. Scale bar = 100 μm.

(G–I) SA-β-gal staining of BPH-1 and RWPE-1 cells in the control, HIF-1α overexpression, and HIF-1α overexpression + si*HIF1A* groups. Scale bar = 100 μm. All data are presented as the mean ± standard deviation, n = 3. *P < 0.05, **P < 0.01, ***P < 0.001; #P < 0.05, ##P < 0.01, ###P < 0.001.

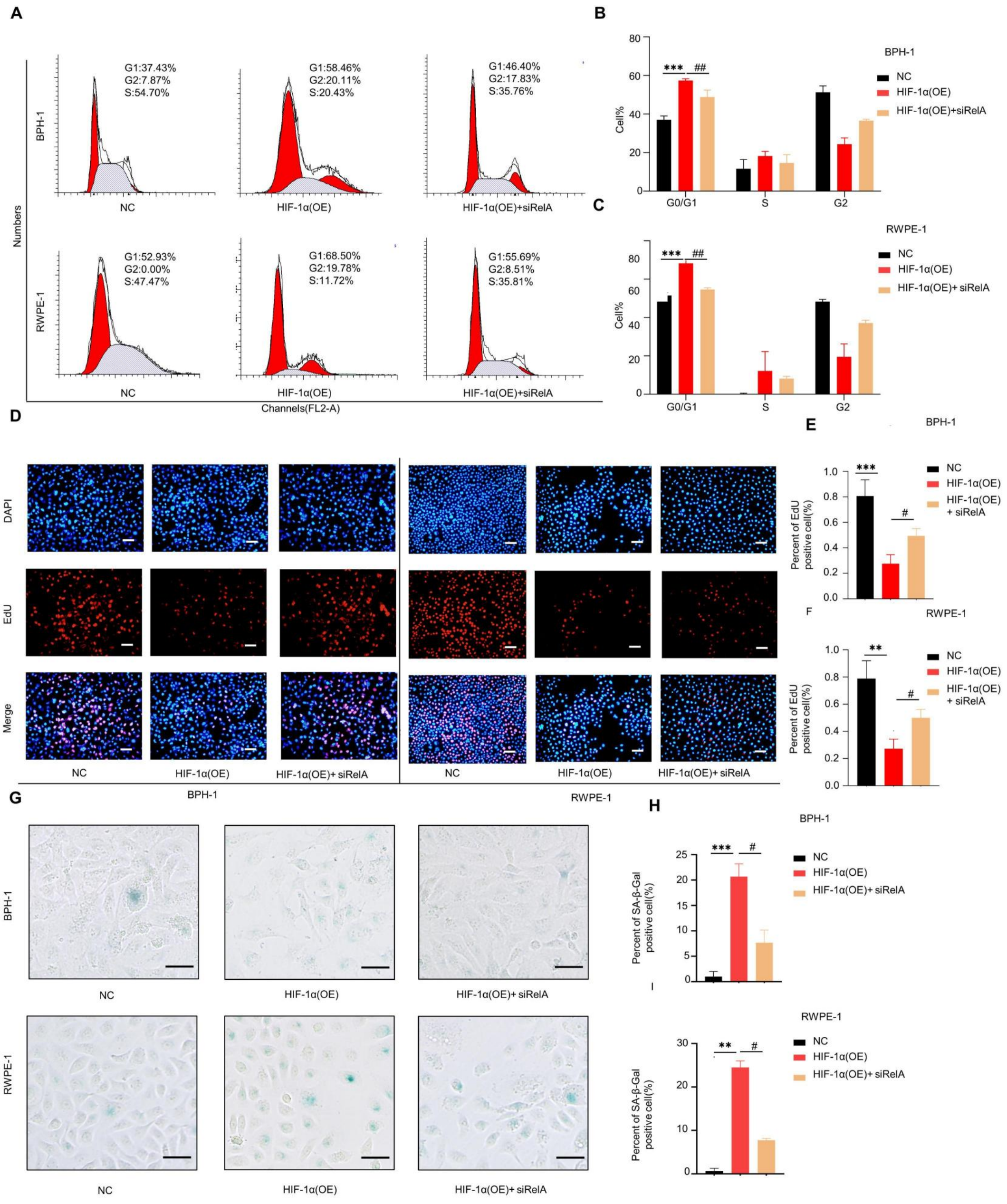

Supplementary Figure S12. NF-κB signaling contributes to HIF-1α–induced senescence-associated phenotypes in prostate epithelial cells.

(A–C) Flow cytometric analysis showing the cell-cycle distribution of BPH-1 and RWPE-1 cells in the control, HIF-1α overexpression, and HIF-1α overexpression + si*RELA* groups.

(D–F) EdU assays showing the proliferation of BPH-1 and RWPE-1 cells in the control, HIF-1α overexpression, and HIF-1α overexpression + si*RELA* groups. Scale bar = 100 μm.

(G–I) SA-β-gal staining of BPH-1 and RWPE-1 cells in the control, HIF-1α overexpression, and HIF-1α overexpression + si*RELA* groups. Scale bar = 100 μm.

All data are presented as the mean ± standard deviation, n = 3. *P < 0.05, **P < 0.01, ***P < 0.001; #P < 0.05, ##P < 0.01.

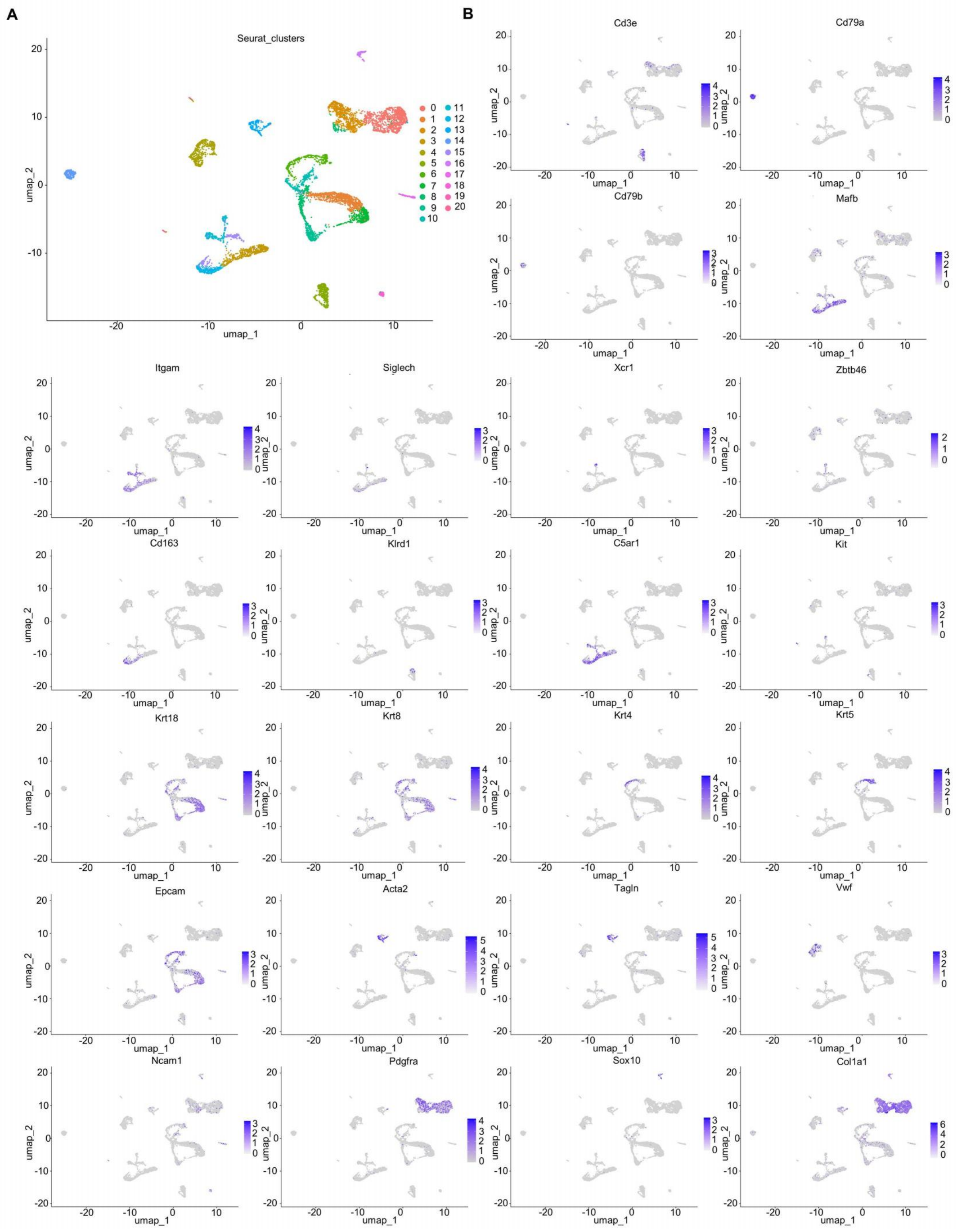

Supplementary Figure S13. Single-cell transcriptomic atlas of castration plus testosterone-induced BPH in ICR male mice.

(A) UMAP plot showing 7,316 cells classified into nine major cell lineages based on marker-gene expression. The identified cell types include T/NK cells, B cells, myeloid cells, epithelial cells, mast cells, myofibroblasts, fibroblasts, smooth muscle cells, and endothelial cells.

(B) Feature plots of representative marker genes used to define each cell type,

including *Il2rb* and *Nkg7* for T/NK cells, *Cd19* and *Pax5* for B cells, *Mpeg1* and *Clec4a2* for myeloid cells, *Krt18* and *Krt8* for epithelial cells, *Cma1* and *Tpsab1* for mast cells, *Acta2* and *Pdgfra* for myofibroblasts, *Col1a1* for fibroblasts, *Rgs5* and *Actg2* for smooth muscle cells, and *Cdh5* and *Sox18* for endothelial cells. Expression intensity is shown on a blue gradient, with darker shades indicating higher expression levels.

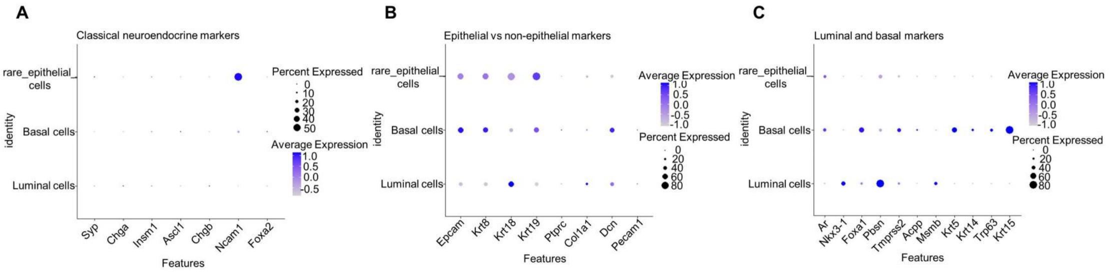

Supplementary Figure S14. Marker-based support for reannotation of the minor epithelial cluster as rare epithelial cells in the mouse prostate scRNA-seq dataset.

(A) Dot plot showing expression of classical neuroendocrine markers, including *Syp*, *Chga*, *Insm1*, *Ascl1*, *Chgb*, *Ncam1*, and *Foxa2*, across luminal, basal, and rare epithelial cell populations. The rare epithelial cluster does not show a coherent canonical neuroendocrine marker program.

(B) Dot plot comparing epithelial and non-epithelial markers across luminal, basal, and rare epithelial cell populations. Rare epithelial cells retain epithelial-marker expression, including *Epcam*, *Krt8*, *Krt18*, and *Krt19*, and do not show clear enrichment of non-epithelial markers such as *Ptprc*, *Col1a1*, *Dcn*, and *Pecam1*.

(C) Dot plot showing representative luminal and basal markers across epithelial subtypes. Rare epithelial cells do not fully conform to either the canonical luminal program, represented by *Ar*, *Nkx3-1*, *Pbsn*, *Tmprss2*, *Acpp*, and *Msmb*, or the basal program, represented by *Krt5*, *Krt14*, *Trp63*, and *Krt15*. These findings support their conservative annotation as a distinct rare epithelial population.

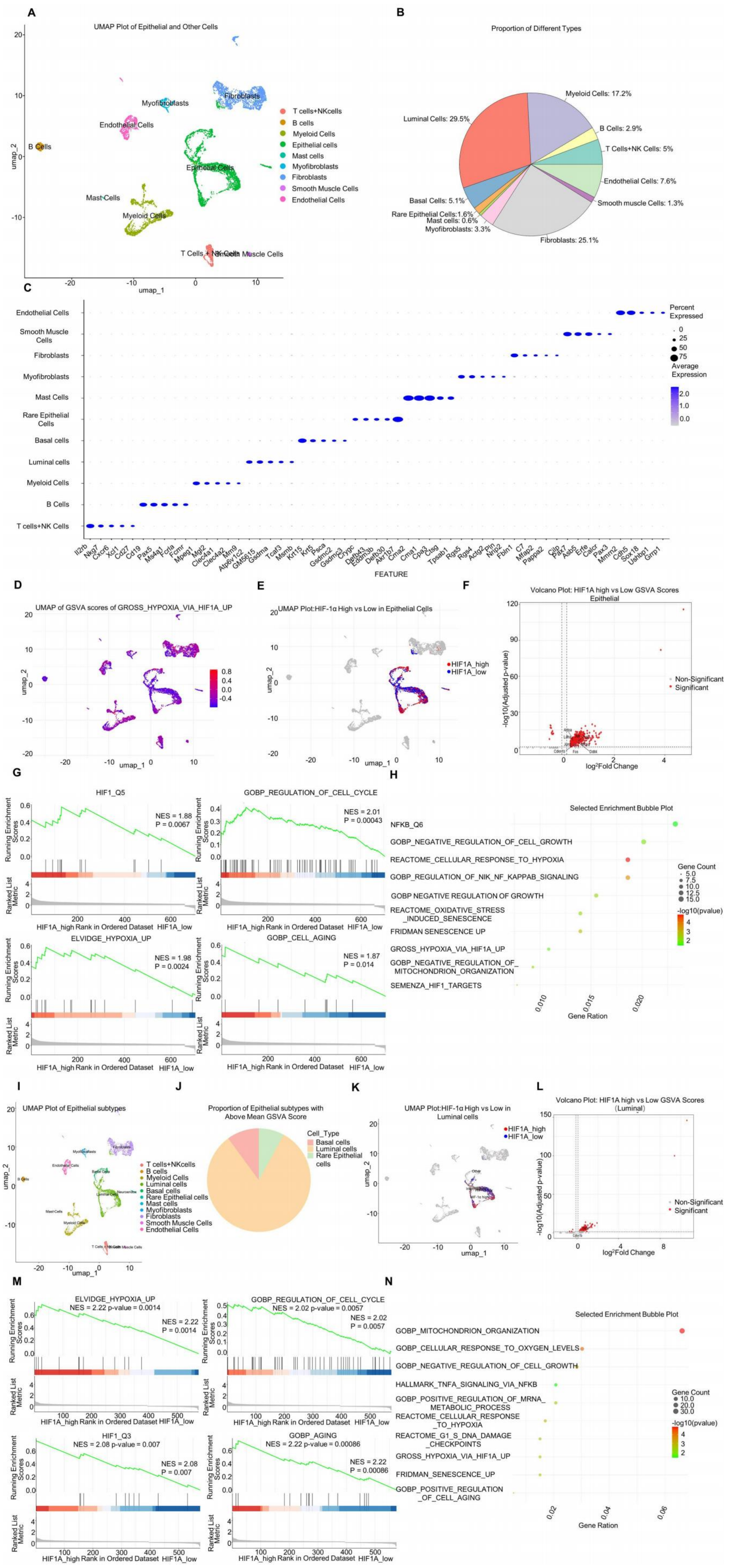
A
UMAP Plot of Epithelial and Other Cells
T cells+NKcells
B cells
Myeloid Cells
Epithelial cells
Mast cells
Myofibroblasts
Fibroblasts
Smooth Muscle Cells
Endothelial Cells
B
Proportion of Different Types
Myeloid Cells: 17.2%
Luminal Cells: 29.5%
B Cells: 2.9%
T Cells+NK Cells: 5%
Endothelial Cells: 7.6%
Basal Cells: 5.1%
Smooth muscle Cells: 1.3%
Rare Epithelial Cells: 1.6%
Mast cells: 0.6%
Myofibroblasts: 3.3%
Fibroblasts: 25.1%
C
Percent Expressed
Average Expression
FEATURE
D
UMAP of GSVA scores of GROSS_HYPOXIA_VIA_HIF1A_UP
E
UMAP Plot:HIF-1α High vs Low in Epithelial Cells
HIF1A_high
HIF1A_low
F
Volcano Plot: HIF1A high vs Low GSVA Scores Epithelial
Non-Significant
Significant
-log10(Adjusted p-value)
log2Fold Change
G
HIF1_Q5
NES = 1.88
P = 0.0067
GOBP_REGULATION_OF_CELL_CYCLE
NES = 2.01
P = 0.00043
ELVIDGE_HYPOXIA_UP
NES = 1.98
P = 0.0024
GOBP_CELL_AGING
NES = 1.87
P = 0.014
Running Enrichment Scores
Ranked List Metric
HIF1A_high Rank in Ordered Dataset HIF1A_low
H
Selected Enrichment Bubble Plot
NFKB_Q6
GOBP_NEGATIVE_REGULATION_OF_CELL_GROWTH
REACTOME_CELLULAR_RESPONSE_TO_HYPOXIA
GOBP_REGULATION_OF_NIK_NF_KAPPAB_SIGNALING
GOBP NEGATIVE REGULATION OF GROWTH
REACTOME_OXIDATIVE_STRESS_INDUCED_SENESCENCE
FRIDMAN SENESCENCE UP
GROSS_HYPOXIA_VIA_HIF1A_UP
GOBP_NEGATIVE_REGULATION_OF_MITOCHONDRION_ORGANIZATION
SEMENZA_HIF1_TARGETS
Gene Count
-log10(pvalue)
Gene Ration
I
UMAP Plot of Epithelial subtypes
T cells+NKcells
B cells
Myeloid Cells
Luminal cells
Basal cells
Rare Epithelial cells
Mast cells
Myofibroblasts
Fibroblasts
Smooth Muscle Cells
Endothelial Cells
J
Proportion of Epithelial subtypes with Above Mean GSVA Score
Cell_Type
Basal cells
Luminal cells
Rare Epithelial cells
K
UMAP Plot:HIF-1α High vs Low in Luminal cells
HIF1A_high
HIF1A_low
L
Volcano Plot: HIF1A high vs Low GSVA Scores (Luminal)
Non-Significant
Significant
M
ELVIDGE_HYPOXIA_UP
NES = 2.22 p-value = 0.0014
GOBP_REGULATION_OF_CELL_CYCLE
NES = 2.02 p-value = 0.0057
HIF1_Q3
NES = 2.08 p-value = 0.007
GOBP_AGING
NES = 2.22 p-value = 0.00086
N
Selected Enrichment Bubble Plot
GOBP_MITOCHONDRION_ORGANIZATION
GOBP_CELLULAR_RESPONSE_TO_OXYGEN_LEVELS
GOBP_NEGATIVE_REGULATION_OF_CELL_GROWTH
HALLMARK_TNFA_SIGNALING_VIA_NFKB
GOBP_POSITIVE_REGULATION_OF_MRNA_METABOLIC_PROCESS
REACTOME_CELLULAR_RESPONSE_TO_HYPOXIA
REACTOME_G1_S_DNA_DAMAGE_CHECKPOINTS
GROSS_HYPOXIA_VIA_HIF1A_UP
FRIDMAN_SENESCENCE_UP
GOBP_POSITIVE_REGULATION_OF_CELL_AGING
Gene Count
-log10(pvalue)
Gene Ration

Supplementary Figure S15. Single-cell RNA sequencing reveals hypoxia-, NF-κB-, and senescence-associated transcriptional programs in castration plus testosterone-induced BPH.

(A) UMAP plot showing the distribution of epithelial and non-epithelial cell types identified in castration plus testosterone-induced BPH. Cells were classified into T/NK cells, B cells, myeloid cells, epithelial cells, mast cells, myofibroblasts, fibroblasts, smooth muscle cells, and endothelial cells.

(B) Pie chart showing the proportions of identified cell types. Epithelial cells accounted for the largest proportion, including Luminal cells, Basal cells, and rare epithelial cells, followed by fibroblasts, myeloid cells, and smaller contributions from other cell types.

(C) Dot plot showing expression of representative marker genes across the nine cell lineages. Immune-related markers such as *Il2rb* and *Nkg7* were enriched in T/NK cells, whereas *Cd19* and *Pax5* marked B cells. *Mpeg1* and *Clec4a2* highlighted myeloid cells, and epithelial markers such as *Krt18*, *Krt8*, and *Krt5* revealed heterogeneity within the epithelial compartment. Stromal markers such as *Col1a1* and vascular markers such as *Cdh5* confirmed the identities of fibroblasts and endothelial cells, respectively. Additional marker-expression analyses supporting the reannotation of rare epithelial cells are shown in Fig. S14A–C.

(D) UMAP plot showing GSVA scores for the GROSS_HYPOXIA_VIA_HIF1A_UP gene set across different cell types, with prominent hypoxia pathway activation observed in epithelial and stromal cells.

(E) UMAP plot of epithelial cells stratified into HIF-1α pathway-high and HIF-1α pathway-low groups based on GSVA scores.

(F) Volcano plot showing DEGs between HIF-1α pathway-high and HIF-1α pathway-low epithelial cells. Representative genes related to hypoxia/HIF signaling, NF-κB-associated inflammatory responses, and senescence/cell-cycle regulation are highlighted, including *Aldoa*, *Pgk1*, *Ldha*, *Ddit4*, *Nfkbia*, *Cdkn1b*, *Jun*, *Junb*, and *Fos*.

(G) GSEA plots showing pathways enriched in HIF-1α pathway-high epithelial cells.

Representative enriched pathways include ELVIDGE_HYPOXIA_UP, GOBP_REGULATION_OF_CELL_CYCLE, and GOBP_CELL_AGING, reflecting hypoxia adaptation, cell-cycle regulation, and aging-associated transcriptional programs.

(H) Bubble plot of enriched pathways in HIF-1α pathway-high epithelial cells, highlighting REACTOME_CELLULAR_RESPONSE_TO_HYPOXIA, FRIDMAN_SENESCENCE_UP, and HALLMARK_TNFA_SIGNALING_VIA_NFKB.

(I) UMAP projection of epithelial subtypes identified in the mouse prostate scRNA-seq dataset, including Luminal cells, Basal cells, and rare epithelial cells.

(J) Pie chart showing the proportion of epithelial subtypes with above-mean GSVA scores for the GROSS_HYPOXIA_VIA_HIF1A_UP pathway. Luminal cells comprised the majority, reflecting their dominant contribution to hypoxia-associated pathway activity in the epithelial compartment.

(K) UMAP plot of Luminal cells stratified into HIF-1α pathway-high and HIF-1α pathway-low groups based on GSVA scores.

(L) Volcano plot showing DEGs between HIF-1α pathway-high and HIF-1α pathway-low Luminal cells, highlighting representative significant genes, including *Cdkn1b* and *Ddit4*.

(M) GSEA plots for Luminal cells, showing enrichment of hypoxia-responsive pathways, including ELVIDGE_HYPOXIA_UP; cell-cycle regulation, including GOBP_REGULATION_OF_CELL_CYCLE; and aging-related processes, including GOBP_AGING.

(N) Bubble plot of enriched pathways in HIF-1α pathway-high Luminal cells, highlighting hypoxia response, represented by REACTOME_CELLULAR_RESPONSE_TO_HYPOXIA; NF-κB signaling, represented by HALLMARK_TNFA_SIGNALING_VIA_NFKB; and senescence, represented by FRIDMAN_SENESCENCE_UP.
